# Novel Adenine–Hydrazone Hybrids Against Human Lung Adenocarcinoma (A549): Design, Synthesis, Cellular Mechanistic Investigation and Molecular Docking Studies

**DOI:** 10.3390/ph19030474

**Published:** 2026-03-13

**Authors:** Emre Menteşe, Nedime Çalışkan, Didem Aksu, Mustafa Emirik, Adem Güner, Fatih Yılmaz

**Affiliations:** 1 Department of Chemistry, Faculty of Art and Sciences, Recep Tayyip Erdogan University, 53100 Rize, Turkey; emre.mentese@erdogan.edu.tr (E.M.); nedime_caliskan19@erdogan.edu.tr (N.Ç.); mustafa.emirik@erdogan.edu.tr (M.E.); 2 Central Research Testing and Analysis Laboratory Research and Application Center, Ege University, 35100 Izmir, Turkey; didem.eroglu@ege.edu.tr; 3 Department of Occupational Health and Safety, Faculty of Health Sciences, Sinop University, 57000 Sinop, Turkey; ademguner@sinop.edu.tr; 4 Department of Chemistry and Chemical Process Technology, Vocational School of Technical Sciences, Recep Tayyip Erdogan University, 53100 Rize, Turkey

**Keywords:** adenine, lung cancer, hydrazone, molecular hybridization, cell cycle, LDH assay, molecular docking

## Abstract

**Background/Objectives**: Adenine derivatives are promising anticancer scaffolds, but their cellular mechanisms remain unclear. This study aimed to synthesize adenine–hydrazone hybrids and evaluate their cytotoxic effects in human lung adenocarcinoma (A549) cells. **Methods**: A series of adenine–hydrazone compounds (**3a**–**r**) was synthesized and tested for cytotoxicity in A549 and MRC-5 cells. Selected compounds were further analyzed for LDH release, oxidative stress markers, ROS production, mitochondrial membrane potential, cell-cycle distribution, apoptosis, and in silico docking against VEGFR2, ALK5, and EGFR. **Results**: Compounds with electron-withdrawing or donor–acceptor substituents showed the highest cytotoxicity, while halogenated and methoxy analogs were moderately active. Among the synthesized derivatives, 4F-substituted derivatives (**3c**) showed more activity than 2F- and 3F-substituted ones (**3a** and **3b**). 4F- and 3Br-substituted derivatives (**3f**) showed more activity than only 4F-substituted ones (**3c**). 4-Nitro-substituted derivative (**3i**) showed more activity than 4F- (**3c**), 4Cl- (**3d**) and 4OMe- (**3h**) derivatives. Trimethoxy-substituted derivative (**3l**) showed more activity than di- and mono-substituted methoxy derivatives (**3g**, **3h**, **3j** and **3k**). Among the salicyl aldehydederivatives (**3m**–**r**), 4-N(et)_2_-substituted derivative (**3r**) showed more activity than non-substituted (**3m**), 5Br-(**3n**), 5Cl-(**3o**), 5Me (**3p**) and 3OCH_3_ (**3q**) derivatives. Treatment induced oxidative stress, mitochondrial depolarization, Sub-G1 cell-cycle accumulation, and apoptosis. Docking studies indicated strong binding to VEGFR2 and ALK5, suggesting dual inhibition as a potential mechanism. **Conclusions**: Adenine–hydrazone derivatives exert substituent-dependent anticancer effects by inducing redox imbalance-associated mitochondrial dysfunction and regulated cell death. These results highlight their potential as lead structures for lung cancer therapy.

## 1. Introduction

Cancer is one of the leading causes of mortality worldwide. In 2022, approximately 20 million new cancer cases were diagnosed, and 9.74 million deaths were attributed to cancer globally. According to estimates from the World Health Organization (WHO) and the International Agency for Research on Cancer (IARC), the global cancer burden is projected to reach 35 million cases by 2050, representing an increase of nearly 50% compared to 2022 [1,2]. A similar rise is expected in cancer-related mortality. Currently, cancer ranks as the second most common cause of death worldwide. In response to this growing health challenge, extensive efforts have been devoted to the discovery of novel bioactive compounds, many of which have been approved by the Food and Drug Administration (FDA) for clinical use. Despite these advances, the effectiveness of existing chemotherapy regimens is often limited by drug resistance and severe adverse side effects. Consequently, there remains a need to identify novel small-molecule scaffolds and to better understand their cellular mechanisms of action [3,4,5].

Lung cancer remains one of the most significant public health challenges worldwide. The primary risk factor for lung cancer remains tobacco smoking, which is estimated to be responsible for roughly 85–90% of lung cancer cases. Exposure to secondhand smoke, air pollution, occupational carcinogens (such as asbestos and radon), and genetic susceptibility also contribute significantly [6,7]. Despite progress in cancer screening and treatment, its high incidence and mortality make it a persistent concern for health systems. Lung cancer accounts for more than 12% of all new cancer diagnoses and nearly 18–19% of cancer-related deaths globally. In 2022, approximately 2.5 million individuals were diagnosed with lung cancer and 1.8 million deaths were recorded. In Türkiye, around 30,000 new lung cancer cases and 23,000 deaths occur annually. Only a small proportion of cases (approximately 17–18%) are detected at an early localized stage, whereas the majority are diagnosed after regional or distant spread. Tumor progression involves processes such as angiogenesis, which enables tumor growth and dissemination by providing a sufficient blood supply [8,9]. Angiogenesis has been identified as a contributing factor to the development of numerous diseases, including cancer. This is a significant mechanism employed by tumors to facilitate growth and dissemination. It has been demonstrated that cancerous cells can promote angiogenesis to secure a blood supply that supports their growth and allows them to survive, especially as they increase in size [10].

Despite significant advances in anticancer drug discovery, several challenges remain, including limited effectiveness, systemic toxicity, and drug resistance [11,12]. As a result, one of the most important areas of current cancer research is the development of new, potent anticancer agents with great selectivity. The development of effective anticancer compounds is primarily driven by the need to overcome critical limitations associated with currently available cancer therapies, particularly severe systemic toxicity, limited selectivity, and the emergence of multidrug resistance. These challenges often lead to reduced therapeutic efficacy and treatment failure, highlighting the necessity for innovative drug design strategies that can improve clinical outcomes while minimizing adverse effects [13,14]. In this context, molecular hybridization has emerged as a powerful and well-established medicinal chemistry approach for the rational design of novel bioactive molecules. This strategy involves the covalent integration of two or more distinct pharmacophoric moieties, each possessing known biological activity, into a single hybrid scaffold. The resulting hybrid compounds often exhibit enhanced biological potency, improved target selectivity, and synergistic or multifunctional pharmacological effects compared to their parent molecules [2,15,16,17,18,19].

The purine scaffold represents a fundamental structural motif present in numerous biologically active molecules derived from both natural and synthetic sources. Owing to its structural versatility and biological relevance, purine has attracted considerable attention in medicinal chemistry and drug discovery, and purine-based compounds exhibit diverse pharmacological activities [20,21,22,23]. Adenine is a fundamental purine nucleobase that plays a central role in numerous biological processes. As a key structural component of DNA and RNA, adenine is directly involved in genetic information storage and transfer. Beyond its role in nucleic acids, adenine is also an essential constituent of biologically important molecules such as ATP, NAD^+^, FAD, and coenzyme A, which are critical for cellular energy metabolism and redox balance. Due to its structural compatibility with endogenous nucleotides, adenine-based derivatives can interact with enzymes involved in nucleic acid synthesis, kinases, and other regulatory proteins associated with cell proliferation. Consequently, purine and adenine analogs have been extensively investigated for their anticancer, antiviral, and antimicrobial activities. In particular, the ability of adenine derivatives to interfere with DNA replication and cell cycle progression makes them attractive scaffolds for the development of novel anticancer agents [24,25,26].

**Figure 1:**
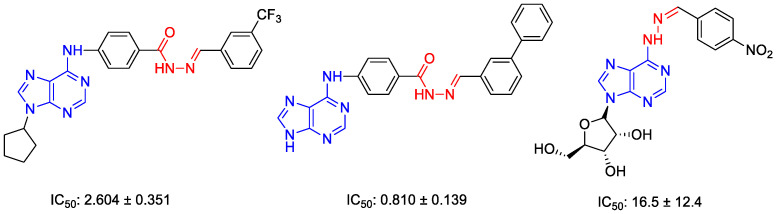
Some reported purine-based hydrazones that are active for lung cancer (A549) cell lines.

Hydrazones, characterized by an azomethine group linked to a carbonyl moiety, exhibit remarkable chemical versatility that enhances both their pharmacological potential and their value as intermediates in coordination chemistry and heterocyclic synthesis. The structural features of hydrazones play a pivotal role in determining their chemical and physical properties primarily attributed to the nitrogen atoms, which are of different natures, and the conjugated C=N containing a lone pair of electrons on the terminal nitrogen atom. In recent years, hydrazone derivatives have attracted significant attention as promising anticancer agents, owing to their ability to interact with multiple cellular targets involved in cancer progression. Numerous studies have demonstrated hydrazones and their metal complexes exhibit potent anticancer activity against a variety of cancer cell lines, including breast, lung, colon, prostate, and leukemia cells [27,28,29,30,31]. The anticancer mechanisms of hydrazones are multifaceted and include the induction of apoptosis, cell cycle arrest, inhibition of angiogenesis, and suppression of tumor cell proliferation [30,32]. In Figure 1, some reported purine-based hydrazones that are effective against lung cancer are given [23,33].

In contrast to conventional hydrazone derivatives, purine–hydrazones combine a purine pharmacophore with a hydrazone linkage, thereby generating a hybrid system with enhanced hydrogen-bonding potential, extended conjugation, and increased structural rigidity [34,35,36,37]. The incorporation of the adenine core introduces multiple donor/acceptor sites and π-interaction capability, potentially enabling biomimetic interactions with nucleotide-binding proteins and enzymes. These features distinguish purine–hydrazones mechanistically from simple imine-based hydrazone systems. Although numerous hydrazone derivatives have been reported with diverse biological activities, most are derived from simple aromatic aldehyde–hydrazide frameworks lacking a biologically privileged heterocyclic scaffold such as purine [38,39]. Furthermore, despite the high global burden of lung cancer, particularly non-small-cell lung cancer (NSCLC), studies evaluating adenine-based hydrazone derivatives against lung cancer cell lines remain limited. Therefore, the present study explores a structurally differentiated purine–hydrazone scaffold and its activity in lung cancer models, contributing to an underexplored area of anticancer research.

Building on our extensive experience in the design and synthesis of novel hybrid molecules [2,9,40,41,42,43,44,45,46,47], the present study focuses on the development of new bioactive adenine hydrazones, with the aim of identifying potential anticancer agents exhibiting enhanced efficacy and reduced side effects. Despite the broad biological potential adenine containing molecules, the cellular mechanisms underlying the cytotoxic effects of adenine-based hydrazone hybrids remain poorly understood, particularly in lung cancer models. Lung cancer is the most common seen cancer type and deadly malignancies worldwide. The current therapies for lung cancer are often limited by toxicity and drug resistance. Therefore, the development of new anticancer agents is urgently needed. Adenine can interfere with nucleic acid metabolism in rapidly proliferating cancer cells. Most previous studies have primarily focused on reporting cytotoxicity, while mechanistic investigations addressing oxidative stress, mitochondrial dysfunction, and regulated cell death processes are limited. Therefore, the present study was designed not only to synthesize and characterize a series of adenine–hydrazone derivatives but also to investigate their biological behavior in A549 lung adenocarcinoma cells. In addition to evaluating cytotoxicity, we aimed to elucidate whether the observed cellular effects are associated with redox imbalance, mitochondrial membrane depolarization, cell cycle alterations, and Annexin V–detectable cell death responses. This approach allows a more comprehensive understanding of the structure–activity relationships and the cellular responses induced by this scaffold.

## 2. Results and Discussion

### 2.1. Chemistry

**Scheme 1:**
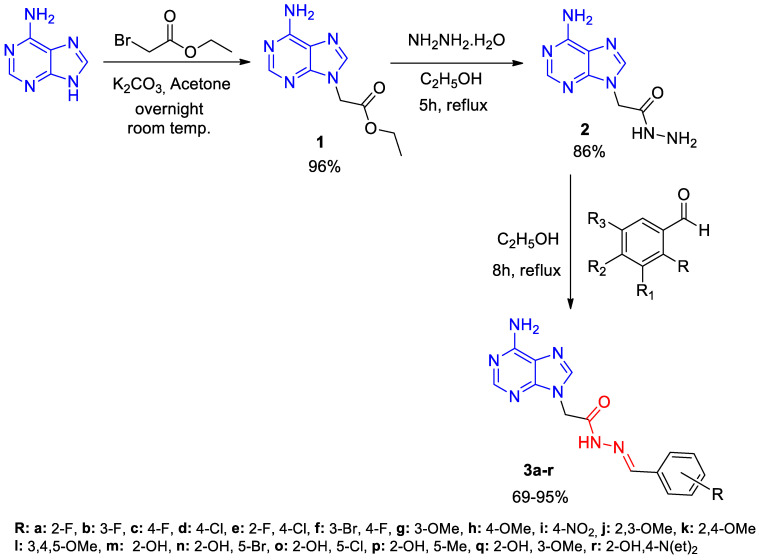
Synthetic route for compounds **3a**–**r**.

Firstly, ethyl 2-(6-amino-9*H*-purin-9-yl)acetate (**1**) compound was synthesized from the reaction of adenine and ethyl bromoacetate in acetone [48]. Then, this compound was reacted with hydrazine monohydrade to obtain 2-(6-amino-9*H*-purin-9-yl)acetohydrazide (**2**) [49]. Lastly, compound **2** was reacted with 18 different aldehyde derivatives in ethanol to obtain novel adenine Schiff bases (**3a**–**r**) (Scheme 1).

All synthesized compounds were tested with TLC for their purity (more than %95) before structural and biological evaluation. Spectral investigation of newly synthesized compounds (**3a**–**r**) are suitable with the proposed structures. When ^1^H NMR spectra of compounds **3a**–**r** were examined, NH_2_ protons of Adenine cycle were observed at about 7.20 ppm. NH and imine CH signals were shown at about 11.80 and 8.20 ppm, respectively. NCH_2_ signals in these compounds were resonant as two sets of signals at about 5.40 and 5.00 ppm. When the ^13^C NMR spectra of compounds **3a**–**r** were examined, it was seen that C=O signal was seen at about 168 ppm. C=N signal was seen at about 156–160 ppm. N=CH carbon signals (Adenin C_2_ and C_6_) were shown at about 142 and 152 ppm, respectively. Imine CH was shown at about 140 ppm. NCH_2_ carbon atom of compounds **3a**–**r** was resonated at about 44 ppm.

**Scheme 2:**
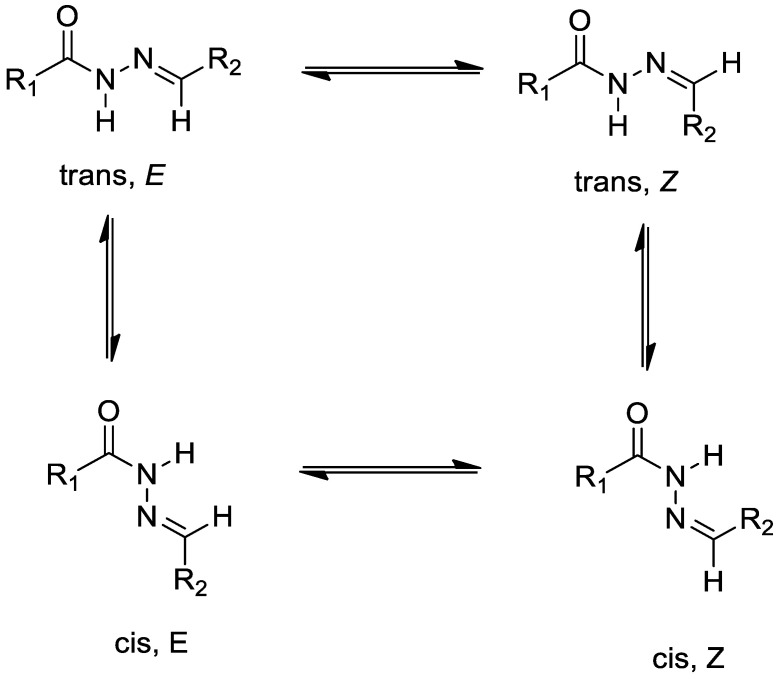
*E*/*Z* geometrical isomers and cis/trans amide conformer of compounds **3a**–**r**.

**Figure 2:**
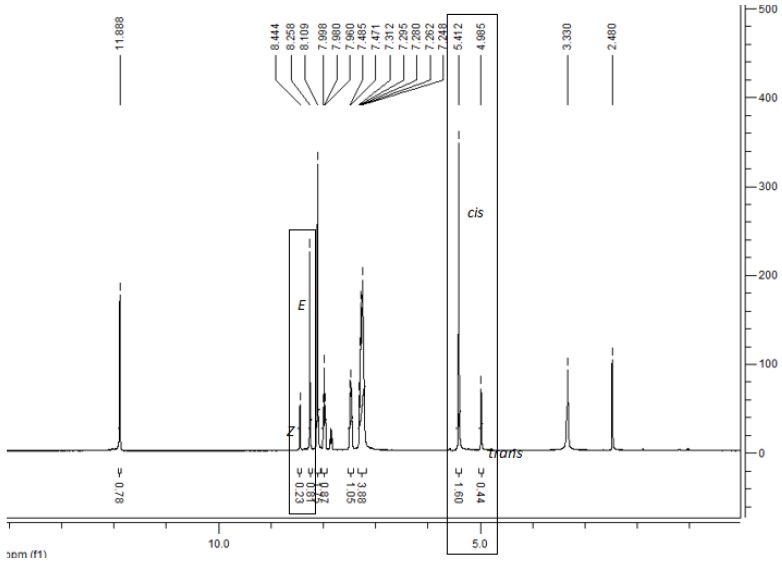
^1^H-NMR spectra of compound **3a**.

In the ^1^H-NMR of compounds **3a**–**r**, it was seen that some of the protons of these compounds have two sets of signals at different ppm. This is coming from the compounds, which have an arylene-hydrazide structure, exist as *E*/*Z* geometrical isomers of the C=N double bond and *cis*/*trans* amide conformers at the CO–NH single bond. According to the literature, compounds that have a C=N double bond prefer the *E* geometrical isomers in DMSO-*d*_6_, and *Z* isomers can be preferred in less polar solvents. N―CH_2_ and N―H signals were observed in two sets of signals due to *cis*/*trans* conformers (Scheme 2) [50,51,52,53,54,55,56,57]. The ratio in each case was calculated by using ^1^H-NMR data. *E*/*Z* and *cis*/*trans* geometrical isomer of compounds **3a**–**r** are shown in Scheme 2. The estimated margin of error in these ratio calculations is approximately ±2–4%, based on signal resolution and reproducibility. The predominance of the *E* isomer in DMSO-*d*_6_ is consistent with previously reported hydrazone derivatives in polar solvents. The duplication of NCH_2_ and NH signals is therefore interpreted as arising from a combination of geometrical isomerism and slow amide bond rotation on the NMR timescale. Also, the HMBC (Heteronuclear Multiple Bond Correlation) spectrum exhibited two sets of long-range correlations corresponding to the *E* and *Z* geometrical isomers. Among them, the E isomer was identified as the major component, based on the stronger correlation signals and higher proton signal intensity in the NMR spectra (see Supplementary File, Figure S39). ^1^H-NMR spectra of compound **3a** were given as examples in Figure 2. In addition, all compounds showed suitable elemental analysis results.

### 2.2. Cell Viability

Among the series, compound **3i** exhibited the highest cytotoxic potency (IC_50_ = 51.1 ± 1.9 µM), followed by **3r** (55.9 ± 2.0 µM) and **3f** (58.8 ± 2.4 µM). Compounds **3c** and **3l** also showed notable activity (IC_50_ = 68.3 ± 2.7 µM and 65.8 ± 2.6 µM, respectively), whereas derivatives such as **3a** and **3h** displayed comparatively weak antiproliferative effects (IC_50_ ≈ 96 µM) (Table 1). A clearer structure–activity relationship was observed when the derivatives were evaluated according to their substitution patterns. Within the fluoro-substituted group (**3a**–**c**), the positional effect was evident, with the para-fluoro analog (**3c**) displaying higher potency than the meta (**3b**) and ortho (**3a**) derivatives. Introduction of additional halogen substituents further improved activity; the fluoro–bromo derivative **3f** showed greater potency than mono-halogenated analogs (**3a**–**d**), and the mixed halogen compound **3e** also exhibited moderate activity.

Methoxy-substituted derivatives showed position- and number-dependent effects. Mono-methoxy analogs (**3g** and **3h**) demonstrated relatively weak cytotoxicity, whereas dimethoxy compounds (**3j** and **3k**) displayed improved activity. The trimethoxy derivative **3l** maintained moderate potency, suggesting that increasing substitution density partially restores activity within this group.

In the salicylaldehyde-containing series (**3m**–**r**), substitution at the aromatic ring markedly influenced biological response. The parent hydroxy derivative **3m** showed moderate activity, while halogenated derivatives (**3n** and **3o**) and the methyl analog (**3p**) displayed reduced potency. In contrast, incorporation of a para-diethylamino group (**3r**) significantly enhanced cytotoxic activity, indicating that strong donor–acceptor electronic character contributes to biological effectiveness.

Overall, these findings demonstrate that the antiproliferative activity of the adenine–hydrazone derivatives were closely associated with the electronic nature and spatial arrangement of aromatic substituents (Table 1).

### 2.3. Selection of Compounds for Mechanistic Studies

Compounds selected for further biochemical and mechanistic investigations were prioritized based on their cytotoxic potency against A549 lung adenocarcinoma cells and their ability to represent clear SAR trends observed within the synthesized adenine–hydrazone series. Molecular docking analyses were used as a supportive tool to provide mechanistic insight into the experimental findings, rather than as a primary selection criterion.

Accordingly, compound **3i** was selected as the most potent derivative in the series, representing the positive impact of strong electron-withdrawing groups on cytotoxic activity in A549 cells. Compound **3c** (para-fluoro) was included as a key SAR reference compound, clearly demonstrating the positional effect of halogen substitution on cytotoxic potency when compared with its ortho- and meta-fluoro analogs. To evaluate the influence of multi-halogen substitution, compound **3f** (fluoro–bromo) was selected due to its enhanced cytotoxic activity and strong predicted interactions with EGFR in docking analyses. In addition, compound **3l**, containing a 3,4,5-trimethoxyphenyl moiety, was chosen as the most representative member of the methoxy-substituted series, reflecting the effect of substituent number and distribution on cytotoxicity and SAR trends in A549 cells.

For mechanistic interpretation, compound **3r** was additionally evaluated due to its high cytotoxic potency and its strong predicted binding affinity toward VEGFR2 and ALK5, providing further insight into potential intracellular targets associated with the observed biological effects. Collectively, this selection strategy enabled a focused and representative evaluation of oxidative stress, membrane integrity, and redox imbalance using LDH release, total oxidant status (TOS), and intracellular GSH/GSSG ratio assays.

### 2.4. Membrane Damage and Oxidative Stress-Associated Redox Imbalance

To determine whether the loss of cell viability was associated with biochemical cellular injury, LDH release, total oxidant status (TOS), and intracellular glutathione redox balance (GSH/GSSG) were evaluated in A549 cells following 48 h treatment with selected compounds at their IC_50_ concentrations (Table 2).

Treatment with all compounds significantly increased LDH release compared with control cells (*p* < 0.05), indicating membrane damage. The strongest LDH leakage was observed for compound **3i** (163 ± 9%, 1.65-fold vs. control; *p* < 0.001), followed by **3r** (159 ± 8%, 1.60-fold; *p* < 0.001) and **3f** (147 ± 8%, 1.50-fold; *p* < 0.01). Compounds **3c** and **3l** produced smaller but still significant increases (138 ± 7% and 135 ± 7%, respectively; *p* < 0.05). The extent of LDH release paralleled the cytotoxic potency observed in the MTT assay, confirming that the reduction in viability reflects genuine cellular injury rather than metabolic interference.

To examine whether oxidative stress contributes to this damage, intracellular oxidant levels were assessed by TOS measurement. Basal TOS in untreated cells was 12.0 ± 1.0 µM H_2_O_2_ Eq/L, and all compounds significantly elevated oxidant levels (*p* < 0.05). The largest increase occurred with **3r** (26.5 ± 1.8 µM; 2.21-fold; *p* < 0.001), followed by **3i** (25.0 ± 1.6 µM; 2.08-fold; *p* < 0.001) and **3f** (23.5 ± 1.5 µM; *p* < 0.01), while **3c** and **3l** caused moderate increases. The parallel elevation of LDH and oxidant status suggests that membrane damage is associated with oxidative processes.

Because glutathione represents the principal intracellular antioxidant system, the GSH/GSSG ratio was measured to evaluate redox homeostasis. Control cells showed a ratio of 10.0 ± 0.7, whereas treatment with all compounds significantly reduced this value (*p* < 0.05). The most prominent reductions were observed with **3r** (5.4 ± 0.4; *p* < 0.001) and **3i** (5.8 ± 0.4; *p* < 0.001), followed by **3f** (6.1 ± 0.4; *p* < 0.01), while **3c** and **3l** produced smaller decreases.

The GSH/GSSG ratio reflects intracellular redox status rather than absolute glutathione content; therefore, the present data indicate redox imbalance but do not allow direct quantification of total glutathione depletion. Accordingly, the findings should be interpreted as evidence of altered redox homeostasis accompanying cytotoxicity.

Taken together, the coordinated increase in membrane damage, elevation of intracellular oxidants, and disruption of redox balance demonstrate that the cytotoxic effects of the adenine–hydrazone derivatives are associated with oxidative stress-related cellular injury. These observations further support the involvement of mitochondrial dysfunction and regulated cell-death responses, which were investigated in subsequent experiments.

### 2.5. Intracellular Reactive Oxygen Species (ROS) Generation

**Figure 3:**
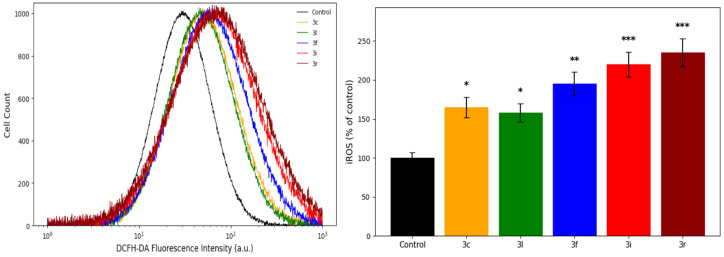
Effects of selected compounds on intracellular reactive oxygen species (iROS) levels in A549 cells. Intracellular ROS production was assessed by DCFH-DA staining followed by flow cytometric analysis after 48 h treatment at IC_50_ concentrations. Data are expressed as percentage of control and presented as mean ± SD of three independent experiments (n = 3). Statistical analysis was performed using one-way ANOVA followed by Tukey’s post hoc test. * *p* < 0.05, ** *p* < 0.01, *** *p* < 0.001 compared with control.

To further clarify whether oxidative stress originates from intracellular processes, reactive oxygen species (ROS) production was measured using DCFH-DA staining following 48 h treatment with the selected compounds (Figure 3).

Exposure to all compounds significantly increased intracellular ROS levels (*p* < 0.05). The highest ROS production was observed in cells treated with compound **3r**, which increased fluorescence intensity to 235 ± 18% of control (*p* < 0.001). Compound **3i** similarly elevated ROS levels to 220 ± 16% (*p* < 0.001), whereas **3f** produced a lower but still significant increase (195 ± 15%; *p* < 0.01). Compounds **3c** and **3l** caused moderate yet significant elevations in ROS generation (165 ± 13% and 158 ± 12%, respectively; *p* < 0.05).

Notably, the pattern of ROS production was consistent with the TOS increase and glutathione depletion observed in the previous assays, supporting that the observed redox imbalance is accompanied by increased intracellular oxidant generation. Collectively, these findings indicate that oxidative stress is a prominent component of the cellular response to compound exposure and may contribute to downstream mitochondrial alterations and regulated cell-death-associated processes.

### 2.6. Mitochondrial Membrane Potential (ΔΨm)

**Figure 4:**
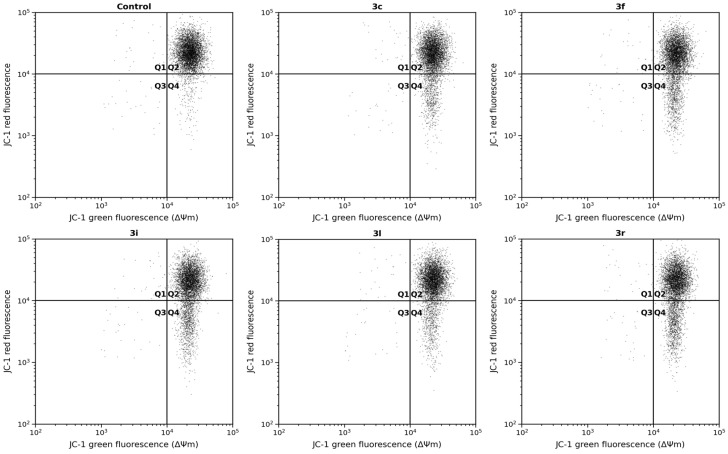
Representative JC-1 flow cytometry dot plots of ΔΨm in A549 cells following 48 h exposure to IC_50_ concentrations of compounds **3c**, **3l**, **3f**, **3i** and **3r**. Treated cells show a shift from polarized to depolarized mitochondrial populations compared with control.

To determine whether intracellular oxidative stress affected mitochondrial integrity, mitochondrial membrane potential (ΔΨm) was evaluated after 48 h exposure to the selected compounds (Figure 4). Treatment with compounds **3c** and **3l** caused moderate decreases in ΔΨm (81.7 ± 5.8% and 84.6 ± 5.2% of control, respectively), whereas compound **3f** produced a more pronounced reduction (73.9 ± 5.7%). The strongest depolarization was observed with **3i** and **3r**, which reduced mitochondrial membrane potential to 65.8 ± 5.1% and 61.9 ± 5.0% of control, respectively.

Importantly, the magnitude of mitochondrial depolarization closely paralleled intracellular ROS production, oxidant accumulation, and glutathione depletion observed in previous assays. Compounds producing the highest ROS levels (**3i** and **3r**) also induced the greatest ΔΨm loss, whereas compounds associated with moderate oxidative changes (**3c** and **3l**) caused only limited depolarization. This graded relationship indicates that mitochondrial dysfunction is not an isolated effect but is functionally linked to intracellular redox imbalance.

Mitochondrial membrane potential is essential for cellular bioenergetic balance, and its reduction reflects impairment of mitochondrial function. However, since ROS and ΔΨm were evaluated at a single time point (48 h), the present data do not establish the temporal order of these events. Therefore, mitochondrial depolarization cannot be interpreted as either a primary trigger or a secondary consequence of cellular injury. Instead, the results demonstrate that mitochondrial dysfunction accompanies oxidative stress-associated cytotoxicity induced by the adenine–hydrazone derivatives.

### 2.7. Cell Cycle Analysis

**Figure 5:**
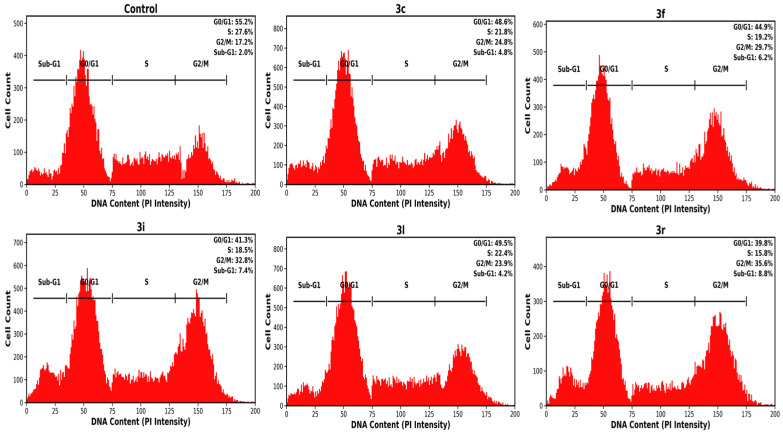
Cell-cycle distribution of A549 cells treated with adenine–hydrazone derivatives. A549 cells were exposed to compounds **3c**, **3l**, **3f**, **3i**, and **3r** at their respective IC_50_ concentrations for 48 h. Cells were stained with propidium iodide (PI) and analyzed by flow cytometry. Representative DNA content histograms showing Sub-G1, G0/G1, S, and G2/M populations are presented.

Cell-cycle distribution of A549 cells following treatment was evaluated by propidium iodide (PI) staining and flow-cytometric DNA content analysis. Representative histograms are presented in Figure 5, and the percentage distribution of cells in Sub-G1, G0/G1, S, and G2/M phases was quantified.

In untreated control cells, most of the population was in the G0/G1 phase (55.2%), followed by the S phase (27.6%) and G2/M phase (17.2%), while only a small fraction of cells appeared in the Sub-G1 region (2.0%), consistent with a predominantly viable and proliferating cell population.

Treatment with compound **3c** produced a moderate redistribution of the cell-cycle profile, characterized by a reduction in the G0/G1 population (48.6%) together with an increase in the G2/M phase (24.8%) and Sub-G1 fraction (4.8%). A similar pattern was observed for compound **3l**, showing a slight decrease in G0/G1 cells (49.5%) accompanied by increases in the G2/M (23.9%) and Sub-G1 (4.2%) populations.

More pronounced alterations were detected following exposure to compounds **3f**, **3i**, and **3r**. Cells treated with **3f** showed a decrease in the G0/G1 fraction (44.9%) and an increase in the G2/M population (29.7%) together with a higher Sub-G1 fraction (6.2%). Treatment with **3i** further accentuated these changes, resulting in accumulation in the G2/M phase (32.8%) and an increased Sub-G1 population (7.4%), accompanied by reductions in both G0/G1 (41.3%) and S phase (18.5%) fractions. The most evident redistribution was observed with **3r**, where G2/M phase cells increased to 35.6% and the Sub-G1 fraction reached 8.8%, with a concomitant decrease in G0/G1 cells (39.8%).

Overall, treatment with the selected adenine–hydrazone derivatives was associated with cell-cycle perturbation characterized by partial G2/M phase accumulation and an increased Sub-G1 population. The rise in the Sub-G1 fraction, which reflects cells with reduced DNA content, is compatible with DNA fragmentation occurring during regulated cell death. Importantly, these findings are consistent with the Annexin V/PI apoptosis analysis and mitochondrial depolarization results and therefore support, rather than independently establish, the apoptosis-associated cytotoxic response observed in A549 cells.

### 2.8. Apoptosis Analysis (Annexin V–FITC/PI Staining)

**Figure 6:**
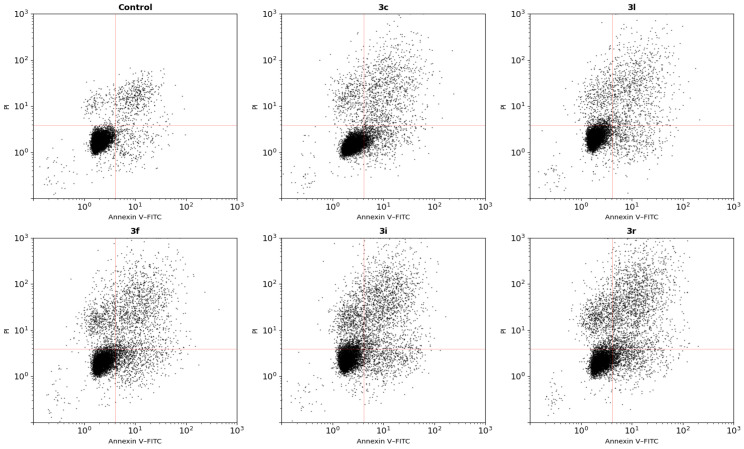
Representative Annexin V–FITC/propidium iodide (PI) flow cytometry dot plots showing the distribution of viable and dead cells after treatment with the tested compounds. Cells were classified into four quadrants: viable cells (Annexin V^−^/PI^−^, lower-left), early apoptotic cells (Annexin V^+^/PI^−^, lower-right), late apoptotic cells (Annexin V^+^/PI^+^, upper-right), and necrotic cells (Annexin V^−^/PI^+^, upper-left). Compared with the control group, treated samples exhibited a shift in the cell population from the viable quadrant toward Annexin V-positive quadrants, particularly the late apoptotic region.

To determine the mode of cell death induced by the compounds, Annexin V–FITC/PI double staining was performed following 48 h treatment (Figure 6). In untreated control cells, most of the population was in the viable quadrant (Annexin V^−^/PI^−^, 88.3%), whereas early apoptotic, late apoptotic, and necrotic populations accounted for 4.7%, 5.6%, and 1.4%, respectively.

Treatment with compound **3c** reduced the viable population to 73.8% and increased early apoptotic (9.2%) and late apoptotic (13.7%) fractions, with only a small necrotic component (3.3%). A similar distribution was observed for **3l**, showing 75.9% viable cells and increases in early apoptotic (8.4%) and late apoptotic (12.9%) cells.

More pronounced effects were detected with **3f**, where viable cells decreased to 65.7%, accompanied by marked elevations in early (11.8%) and late (17.6%) apoptotic populations. Treatment with **3i** further shifted the population toward apoptotic quadrants, reducing viable cells to 59.8% and increasing late apoptosis to 21.1%. The strongest effect was observed with **3r**, which exhibited the lowest viability (57.4%) and the highest late apoptotic fraction (23.6%), while necrotic cells remained comparatively limited (6.2%).

Notably, the increase in Annexin V-positive cells occurred predominantly in the Annexin V-positive quadrants rather than the PI-only quadrant. This distribution is consistent with a regulated cell-death pattern involving phosphatidylserine externalization rather than acute membrane disruption. Furthermore, the magnitude of Annexin V positivity paralleled mitochondrial membrane depolarization and intracellular oxidant elevation, with compounds producing stronger oxidative stress (**3i** and **3r**) also inducing greater shifts toward Annexin V-positive populations.

Taken together, these findings indicate that the loss of viability induced by the adenine–hydrazone derivatives is associated with apoptosis-like regulated cell death linked to intracellular oxidative imbalance and mitochondrial dysfunction, although the precise downstream signaling pathway cannot be conclusively determined from the present data alone.

### 2.9. Molecular Docking Studies

To elucidate the potential mechanism of action at the molecular level and to rationalize the observed in vitro cytotoxic activities, molecular docking simulations were performed. Based on the signaling pathways critical for A549 cell proliferation and metastasis, three key enzymatic targets were selected: Vascular Endothelial Growth Factor Receptor 2 (VEGFR2), Epidermal Growth Factor Receptor (EGFR), and TGF-beta Receptor Type 1 (ALK5). The binding affinities (kcal/mol) are presented in Table 3.

Angiogenesis is a pivotal process in tumor growth, primarily regulated by VEGFR2. The docking results indicated that the synthesized scaffold possesses a high affinity for the VEGFR2 active site, with binding energies generally surpassing −11.0 kcal/mol. Notably, compound **3l**, which exhibited the highest selectivity index in biological assays, demonstrated one of the strongest binding affinities towards VEGFR2 with a score of −12.128 kcal/mol. This strong interaction suggests that the selective cytotoxicity of **3l** may be mediated through the inhibition of angiogenesis pathways in the tumor microenvironment.

**Figure 7:**
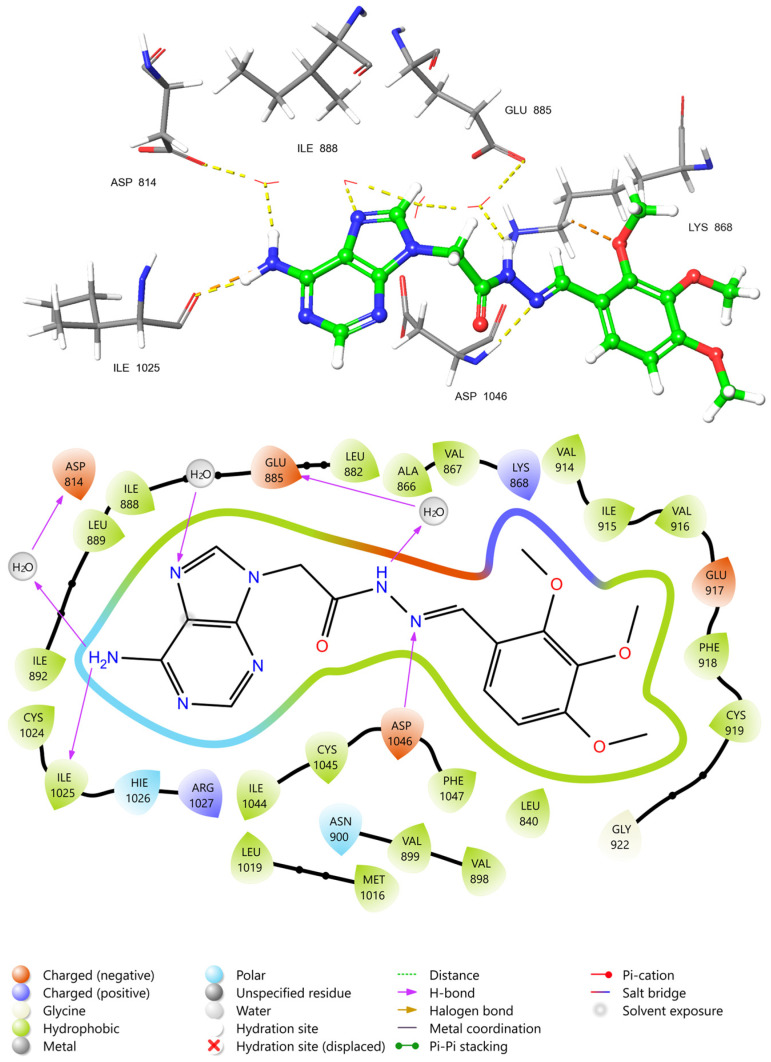
The 2D and 3D interaction diagram of VEGFR2–compound **3l** complex.

To elucidate the molecular basis for the superior selectivity (SI = 1.71) and high binding affinity (−12.128 kcal/mol) of compound **3l**, its binding mode within the active site of VEGFR2 (PDB ID: 4ASD) was analyzed in detail. The 2D ligand-interaction diagram (Figure 7) reveals that compound **3l** occupies the ATP-binding pocket of the kinase domain, establishing a network of crucial hydrogen bonds and hydrophobic contacts that stabilize the complex. The adenine scaffold of **3l** acts as a key anchor, mimicking the adenosine moiety of ATP. A significant hydrogen bond interaction is observed between the exocyclic amino group (NH2) of the adenine ring and the backbone of Ile1025, located in the hinge region of the kinase. This interaction is pivotal for orienting the ligand within the catalytic cleft. Furthermore, a water-bridged hydrogen bond network is established involving the imidazole nitrogen of the adenine core and the conserved Glu885 residue, as well as Asp814. The involvement of these solvent-bridged interactions contributes to the ligand’s conformational stability and specificity. A critical feature of the binding mode is the direct hydrogen bond between the linker hydrazone nitrogen and the side chain of Asp1046. Asp1046 is a component of the conserved DFG (Asp-Phe-Gly) motif, which regulates the activation state of the kinase. The ability of compound **3l** to interact with the DFG-aspartate suggests it may stabilize the kinase in an inactive conformation, a mechanism often associated with potent VEGFR2 inhibitors such as sorafenib. In addition to electrostatic interactions, the binding pose is reinforced by extensive hydrophobic contacts. The trimethoxy-phenyl moiety is deeply embedded in a hydrophobic pocket lined by Val916, Phe918, Cys919, and Gly922. Similarly, the adenine core is flanked by non-polar residues including Leu889, Ile892, Val899, and Leu1019. The proximity of the electron-rich trimethoxy groups to the hydrophobic surface suggests favorable van der Waals interactions, which likely account for the enhanced binding energy observed for **3l** compared to mono-methoxy analogs. Collectively, these structural insights demonstrate that compound **3l** effectively engages the key pharmacophoric features of the VEGFR2 active site, specifically the hinge region (Ile1025) and the DFG motif (Asp1046), providing a rational explanation for its potent in silico score and favorable in vitro profile.

The TGF-beta signaling pathway, mediated by the ALK5 receptor, is known to promote metastasis and epithelial–mesenchymal transition (EMT) in lung cancer. The most potent antiproliferative agent in the series, compound **3i**, exhibited a remarkable binding affinity of −10.985 kcal/mol against ALK5. Furthermore, other active compounds such as **3m** and **3n** showed significant binding energies of −11.959 kcal/mol and −11.707 kcal/mol, respectively. These results suggest that the high potency of **3i** observed in vitro is likely correlated with its ability to effectively block the ATP-binding pocket of the ALK5 kinase domain.

**Figure 8:**
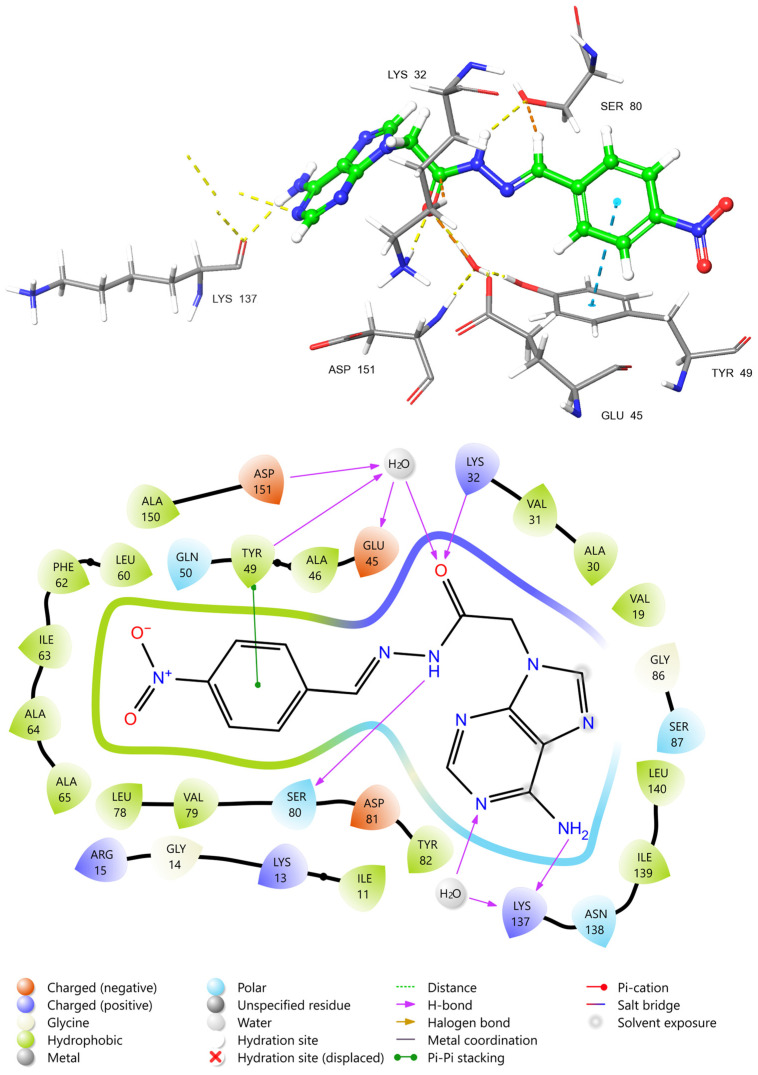
The 2D and 3D interaction diagram of ALK5 receptor–compound **3i** complex.

To rationalize the potent cytotoxic activity of compound **3i** and its high binding affinity towards the TGF-beta Receptor Type 1 (ALK5), the protein-ligand interactions were analyzed using the crystal structure PDB ID: 3HMM. The 2D interaction diagram (Figure 8) reveals that compound **3i** fits well within the ATP-binding pocket, stabilized by a combination of hydrogen bonding networks and hydrophobic enclosure. A critical anchoring interaction is established by the carbonyl oxygen of the amide linker, which acts as a hydrogen bond acceptor for the side chain of the catalytic residue Lys32. This interaction is of particular significance as Lys32 is essential for the phosphotransfer reaction of the kinase; its blockade is a hallmark of effective type I kinase inhibition. Furthermore, this carbonyl group participates in a water-mediated hydrogen bond network involving Asp151 and Glu45, further anchoring the ligand within the catalytic cleft. The hydrazone nitrogen forms a direct hydrogen bond with the hydroxyl group of Ser80, contributing to the stabilization of the linker conformation. On the adenine side of the scaffold, the exocyclic amine and the ring nitrogen engage in hydrogen bonding interactions with Lys137, mediated partly by a structural water molecule. Beyond electrostatic forces, the stability of the complex is significantly enhanced by hydrophobic and pi-stacking interactions. The electron-deficient p-nitrophenyl moiety participates in a strong pi-pi stacking interaction with the aromatic ring of Tyr49. This stacking not only restricts the conformational flexibility of the ligand but also positions the nitro group within a hydrophobic pocket lined by Val19, Ala46, Leu60, and Ile63. The adenine core is similarly flanked by non-polar residues, including Ile11, Val31, and Leu140, creating a favorable van der Waals environment that complements the high binding score observed in silico. In conclusion, compound **3i** effectively mimics the binding mode of known ALK5 inhibitors by targeting the catalytic Lys32 and exploiting the hydrophobic nature of the gatekeeper region via Tyr49 stacking. These specific molecular contacts provide a structural explanation for the superior biological potency of **3i** compared to other derivatives in the series.

EGFR overexpression is frequently associated with non-small-cell lung cancer (NSCLC) progression. While the binding energies for EGFR were generally lower compared to VEGFR2 and ALK5, specific derivatives showed promising poses. Compound **3f**, which displayed a favorable bioactivity profile (IC_50_ = 58.8 µM; SI = 1.48), exhibited a distinct binding score of −10.36 kcal/mol against EGFR, significantly higher than the series average. This suggests that **3f** may act as a dual-target inhibitor and utilize other pathways besides EGFR inhibition to exert its cytotoxic effect.

**Figure 9:**
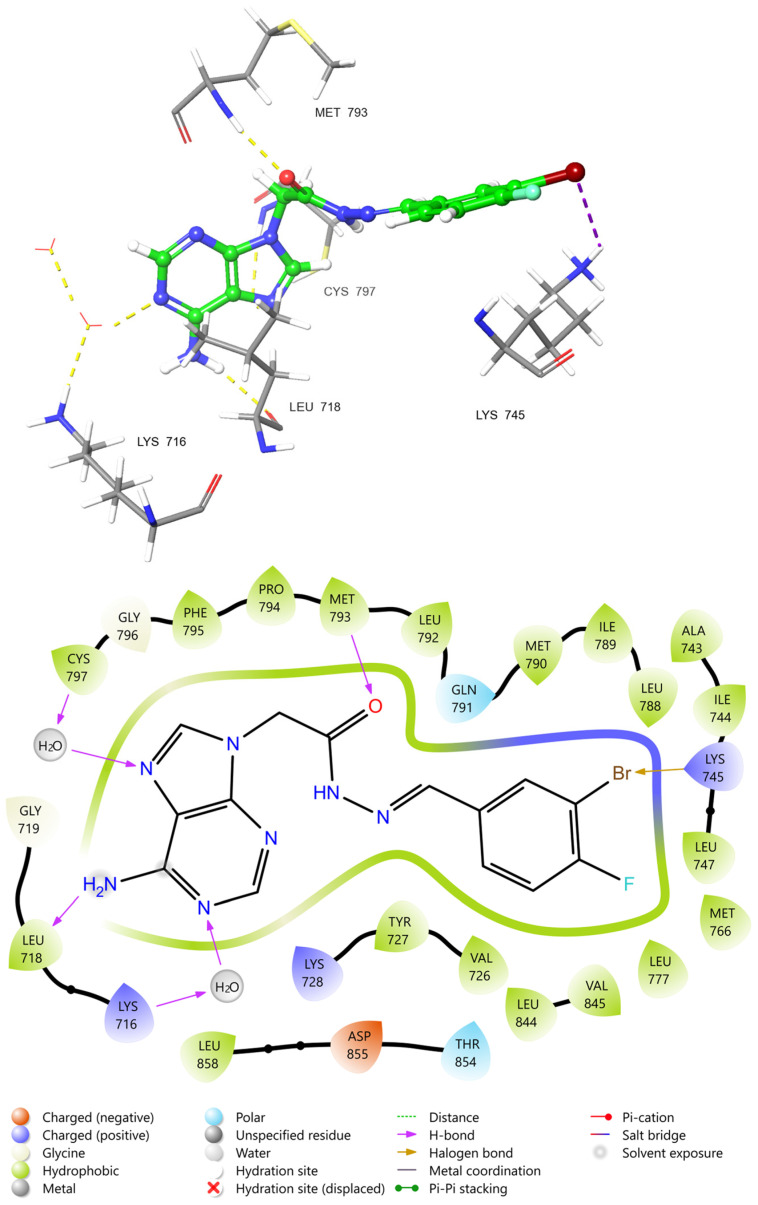
The 2D and 3D interaction diagram of EGFR–compound **3f** complex.

To investigate the molecular determinants behind the significant antiproliferative activity of the halogenated derivative **3f** and its favorable docking score, its binding pose within the active site of EGFR was analyzed using the crystal structure PDB ID: 6V6O. The 2D ligand-interaction diagram (Figure 9) elucidates a distinct binding mode characterized by key hydrogen bonds and a specific halogen interaction. The adenine scaffold of compound **3f** anchors firmly into the hinge region of the kinase. Specifically, the exocyclic amino group forms a direct hydrogen bond with the backbone carbonyl of Met793, a critical residue for adenine-mimetic inhibitors. Additionally, a dual hydrogen bonding interaction is observed involving Leu718, one direct interaction with the adenine amine and a water-mediated bridge involving the ring nitrogen and the side chain of Lys716. Another crucial water-bridged hydrogen bond connects the imidazole nitrogen of the scaffold to Cys797, a residue often targeted by covalent inhibitors, suggesting that **3f** effectively occupies this strategic pocket. A distinguishing feature of the **3f**-EGFR complex is the presence of a halogen bond. The diagram highlights a specific interaction between the bromine atom on the phenyl ring and the cationic side chain of the catalytic residue Lys745. This halogen bond likely contributes significantly to the stability of the complex and explains the enhanced potency of the halogenated derivative compared to its unsubstituted counterparts. Furthermore, the ligand is stabilized by extensive hydrophobic contacts. The aromatic core and the substituted phenyl ring are surrounded by a lipophilic cage formed by Leu718, Gly719, Val726, Ala743, Leu747, Met790, and Phe795. Notably, the interaction with Met790 residue indicates that compound **3f** is capable of accommodating the sterically demanding T790M mutant pocket, which is clinically relevant for drug-resistant non-small-cell lung cancer (NSCLC). Thus, the binding mode of compound **3f** is driven by a combination of classical hinge-binding hydrogen bonds and a unique halogen bond with Lys745, providing a structural rationale for its selective cytotoxic profile and high affinity for the EGFR kinase domain.

### 2.10. Structure–Activity Relationship (SAR) Analysis

The structure–activity relationship (SAR) of the synthesized adenine Schiff base derivatives (**3a**–**r**) was evaluated based on their cytotoxic activities against A549 lung cancer cells and their selectivity toward cancer cells over MR5 lung fibroblasts.

**Figure 10:**
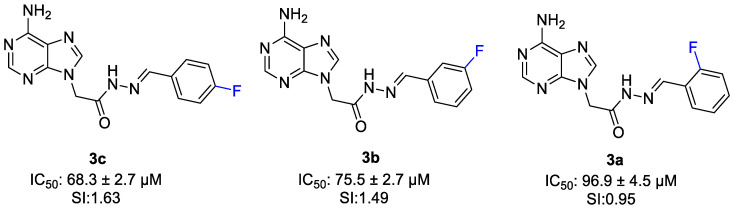
The cytotoxic activity and selectivity index value of compounds **3a**–**c**.

Compounds which have one fluorine substituent on the aromatic ring (**3a**–**c**) exhibited cytotoxic activity with IC_50_ values of 96.9 ± 4.5 µM, 75.5 ± 2.7 µM and 68.3 ± 2.7 µM and showed selectivity index value of 0.95, 1.49 and 1.63. Among the compounds **3a**–**c**, the positional effect of the fluorine substituent on the aromatic ring was seen. The para-fluoro-substituted compound (**3c**) showed the highest cytotoxic activity and selectivity (IC_50_ = 68.3 ± 2.7µM, SI = 1.63), followed by the meta-fluoro analog (**3b**), while the ortho-fluoro derivative (**3a**) exhibited the weakest activity. This trend indicates that para substitution is the most favorable position (Figure 10).

**Figure 11:**
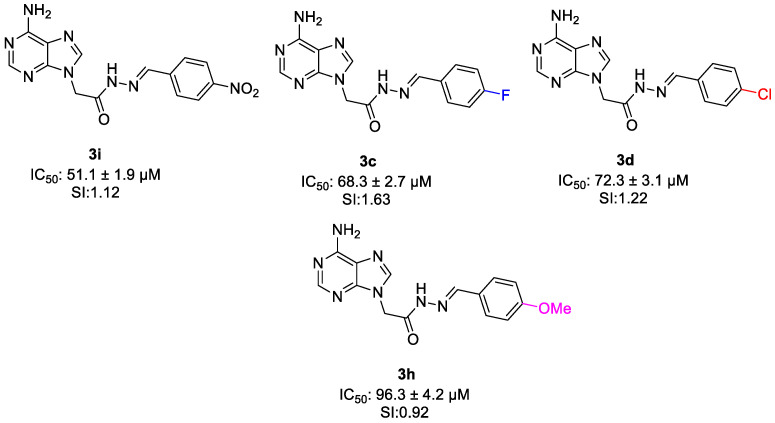
The cytotoxic activity and selectivity index value of compounds **3c**, **3d**, **3h** and **3i**.

When the para-substituted compounds were compared, it was seen that para-nitro derivative (**3i**) exhibited the highest cytotoxic potency (IC_50_ = 51.1 ± 1.9 µM), para-fluoro compound (**3c**) showed moderate activity (IC_50_ = 68.3 ± 2.7 µM), while the para-chloro (**3d**) and para-methoxy (**3h**) derivatives displayed slightly reduced potency with IC_50_ value of 72.3 ± 3.1 µM and 96.3 ± 4.2 µM, respectively. Also, selectivity index order of these compounds is similar with cytotoxic potency. Compound **3c** (para-flouoro-substituted derivative) showed the highest selectivity with 1.63 value and the compound **3h** (para-methoxy-substituted derivative) showed lowest selectivity with 0.92 value. This situation indicates that electron-withdrawing para substituents positively modulate anticancer activity, whereas electron-donating groups reduce potency (Figure 11).

**Figure 12:**
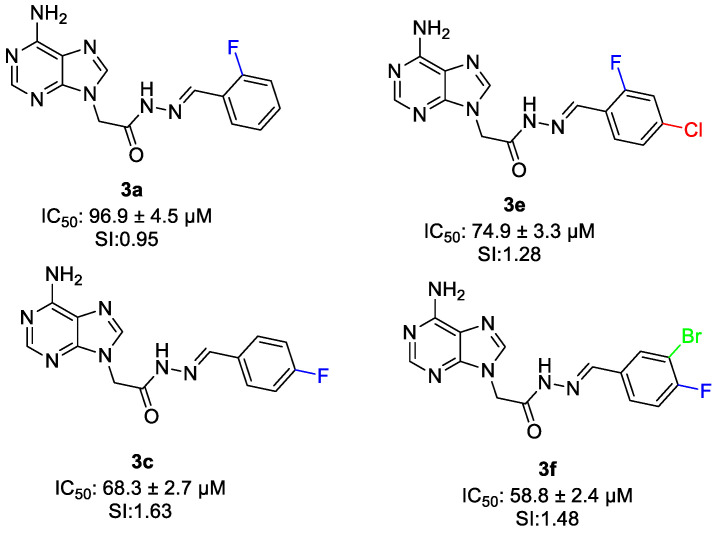
Comparison of the cytotoxic activity and selectivity index value of compounds **3a**–**3e** and **3c**–**3f**.

Comparison of compounds **3a** and **3e** highlights the effect of halogen substitution on anticancer activity. The orto-fluoro-substituted derivative (**3a**) exhibited weak cytotoxic activity and low selectivity (IC_50_ = 96.9 ± 4.5 µM, SI = 0.95), whereas the di-halogen-substituted derivative (orto-fluoro and para-chloro) (**3e**) showed better potency and selectivity (IC_50_ = 74.9 ± 3.3µM, SI = 1.28). A similar trend was seen when comparing compounds **3c** and **3f**. The para-fluoro-substituted compound **3c** displayed the activity 68.3 ± 2.7µM with 1.63 selectivity value, while the para-fluoro and meta-bromo-substituted derivative (**3f**) exhibited enhanced cytotoxic potency (IC_50_ = 58.8 µM) with comparable selectivity (SI = 1.48) (Figure 12). These results show that multi-halogen substitution enhances the anticancer activity.

**Figure 13:**
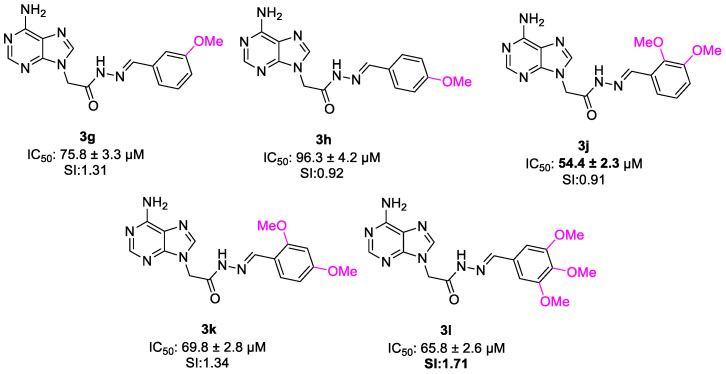
The cytotoxic activity and selectivity index value of compounds **3g**, **3h**, **3j**, **3k** and **3l**.

When the activities of methoxy-substituted derivatives (**3g**, **3h**, **3j**, **3k** and **3l**) were investigated, a clear relationship between the number and positional distribution of methoxy groups and anticancer activity. The mono-methoxy-substituted compounds **3g** (m-OMe) and **3h** (p-OMe) showed relatively low activity (75.8 ± 3.3 µM, SI: 1.31 and 96.3 ± 4.2 µM, SI:0.92, respectively). In contrast, di-methoxy-substituted derivatives **3j** (2,3-OMe) and **3k** (3,4-OMe) exhibited improved potency (54.4 ± 2.3 µM, SI: 0.91 and 69.8 ± 2.8 µM, SI: 1.34, respectively). Notably, the trimethoxy-substituted compound (3,4,5-OMe) **3l** demonstrated the favorable activity and selectivity index value (65.8 ± 2.6 µM, SI: 1.71). These results clearly indicate that the number of methoxy substitutions positively influences the anticancer activity. Also, comparison of the mono-methoxy derivatives **3g** and **3h** indicates that positional variation of a single methoxy group influences cytotoxic activity. The meta substitution resulted in slightly better activity than the para substitution. A similar positional effect was also observed between the di-methoxy derivatives **3j** (2,3-dimethoxy) and **3k** (2,4-dimethoxy). Compound **3j** showed improved cytotoxic activity compared to **3k** (Figure 13). Overall, these comparisons demonstrate that the position of methoxy substituents plays a critical role for biological activity, even when the number of substituents remains constant.

**Figure 14:**
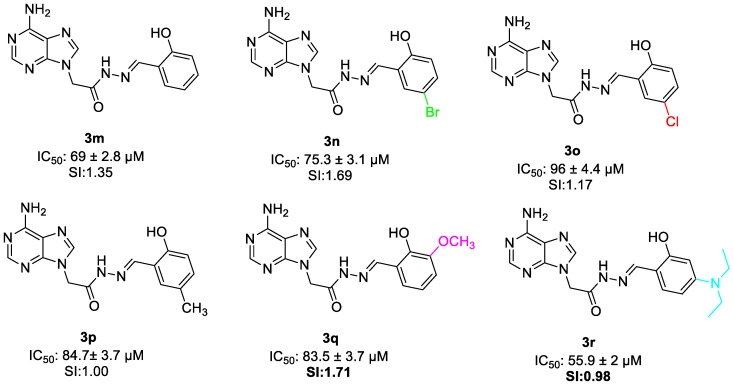
The cytotoxic activity and selectivity index value of compounds **3m**–**r**.

Evaluation of the anticancer activity of compounds **3m**–**r** showed that incorporation of the salicylaldehyde moiety into the adenine scaffold effected the anticancer activity. It has been seen that compound **3m** showed IC_50_ value of 69 ± 2.8 µM and 1.35 selectivity. Addition of bromine (**3n**), chlorine (**3o**), methyl (**3p**) on 5th position of phenyl ring and substitution of methoxy (**3q**) on 3rd position phenyl ring resulted in decreased activity. Although electron-withdrawing substituents generally enhanced cytotoxic activity within this series, compound **3r** demonstrates that strong donor groups capable of inducing donor–acceptor electronic interactions and improving physicochemical properties can also significantly contribute to biological potency (IC_50_ value 55. 9 ± 2 µM) (Figure 14).

## 3. Material and Methods

The ^1^H and ^13^C-NMR spectra were a Varian Mercury 400 MHz spectrometer (Varian, Inc., Palo Alto, CA, USA); the melting points were measured by Stuart SMP30 melting point apparatus (Bibby Scientific Ltd., Stafford, UK); the thin-layer chromatography (TLC) was carried out with Meck silica gel plates (60, F_254_, 0,2 mm) (Merck Millipore, Darmstadt, Germany). All the reagents and solvents were purchased from Merck and Sigma companies (Merck Millipore, Darmstadt, Germany) (Sigma-Aldrich, St. Louis, MO, USA) which were used directly without further purification.

### 3.1. Chemistry

#### 3.1.1. Ethyl 2-(6-Amino-9*H*-purin-9-yl)acetate (**1**)

Adenine (1.35 g, 10 mmol) and K_2_CO_3_ (3.45 g, 25 mmol) were added to dry acetone (30 mL) and stirred for 15 min at room temperature. Then ethyl bromoacetate (1.25 mL, 11 mmol) was added to the mixture and it was stirred overnight at room temperature. Following the addition of water (100 mL) to the final mixture, the precipitate was filtered off. The final product was thoroughly washed with water, after which it was purified by crystallization in ethanol/water (4:1) and dried in a desiccator.

Yield: 2.12 g (96%), m.p.: 225–227 °C, 226–228 °C [48].

#### 3.1.2. 2-(6-Amino-9*H*-purin-9-yl)acetohydrazide (**2**)

Compound 1 (2.21 g, 10 mmol) was dissolved in ethanol (25 mL), and hydrazine monohydrate (25 mmol) was added to the mixture. Then, the mixture was then boiled for 5 h under reflux condition. After the completion of the reaction, the mixture was cooled, and the precipitate was filtered off. The product was purified from ethanol/water (4:1) and dried in a vacuum desiccator.

Yield: (86%), m.p.: 300–301 °C, 296 °C [49], ^1^H-NMR (400 MHz, DMSO-*d*_6_), δ, ppm: 4.58 (s, 2H, NH_2_), 4.77 (s, 2H, NCH_2_), 7.21 (s, 2H, NH_2_), 8.08 (s, 1H, ArH), 8.09 (s, 1H, Ar-H), 9.44 (s, 1H, NH). ^13^C-NMR (100 MHz, DMSO-*d*_6_), δ, ppm: 44.2 (NCH_2_), 118.7 (Ar-C), 142.1 (N=CH), 150.1 (Ar-C), 152.8 (N=CH), 156.3 (C=N), 166.3 (C=O). Elemental analysis: Calculated for C_7_H_9_N_7_O, C, 40.58, H, 4.38, N, 47.32; found: C, 40.51, H, 4.30 N, 47.26.

#### 3.1.3. Synthesis of Compounds **3a**–**r**

Compound 2 (2.07 g, 10 mmol) and ethanol (20 mL) were meticulously transferred into a round-bottom flask, whereupon the mixture was subjected to rigorous stirring for a period of 15 min at ambient temperature. Subsequently, the mixture was treated with 1.1 mmol of the corresponding aldehyde and 2 drops of acetic acid, which functioned as a catalyst. The mixture was subjected to boiling under reflux conditions for a period of 8 h. The final mixture was then allowed to reach room temperature, after which it was immersed in cold water to precipitate the products. The precipitated substance was filtered and then purified by washing with ethanol. The substance was desiccated using CaCl_2_ in a vacuum desiccator.

2-(6-Amino-9*H*-purin-9-yl)-N′-(2-fluorobenzylidene)acetohydrazide (**3a**): Yield: 2.81 g (89%), m.p.: 276–278 °C, ^1^H-NMR (400 MHz, DMSO-*d*_6_), δ, ppm: 5.41 + 4.98 (s, 2H, NCH_2_, cis/trans amid conformer, %88/22), 7.28 (m, 4H, NH_2_ + ArH), 7.31 (m, 1H, ArH), 7.90 (m, 1H, ArH), 8.10 (s, 2H, ArH), 8.25 + 8.44 (s, 1H, N=CH, E/Z amid conformer, %73.5/21.5), 11.88 (s, 1H, NH). ^13^C-NMR (100 MHz, DMSO-*d*_6_), δ, ppm: 44.4 (NCH_2_), 116.4, 118.8, 121.8, 121.9, 125.3, 126.8, 132.4 (Ar-C), 137.6 (N=CH), 150.4 (C=N), 152.9 (N=CH), 156.4 (C=N), 159.9 + 162.4 (C_F_, J = 248.9 Hz), 168.7 (C=O). Elemental analysis: Calculated for C_14_H_12_FN_7_O, C, 52.99, H, 3.81, N, 30.90; found: C, 52.86, H, 3.77, N, 30.82.

2-(6-Amino-9*H*-purin-9-yl)-N′-(3-fluorobenzylidene)acetohydrazide (**3b**): Yield: 2.95 g (93%), m.p.: 300–302 °C, ^1^H-NMR (400 MHz, DMSO-*d*_6_), δ, ppm: 5.42 + 4.98 (s, 2H, NCH_2_, cis/trans amid conformer, %72/28), 7.22 (m, 4H, NH_2_ + ArH), 7.50 (m, 2H, ArH), 7.60 (m, 3H, ArH), 8.04 (s, 1H, ArH), 8.10 + 8.23 (s, 1H, N=CH, E/Z amid conformer, %75/25), 11.87 (s, 1H, NH). ^13^C-NMR (100 MHz, DMSO-*d*_6_), δ, ppm: 44.5 (NCH_2_), 113.2, 117.2, 118.8, 124.0, 131.4, 136.9 (Ar-C), 143.4 (N=CH), 150.4 (C=N), 152.9 (N=CH), 156.3 (C=N), 161.7 + 164.0 (C_F_, J = 228 Hz), 168.2 (C=O). Elemental analysis: Calculated for C_14_H_12_FN_7_O, C, 52.99, H, 3.81, N, 30.90; found: C, 52.85, H, 3.79, N, 30.86.

2-(6-Amino-9*H*-purin-9-yl)-N′-(4-fluorobenzylidene)acetohydrazide (**3c**): Yield: 3.02 g (95%), m.p.: 302–304 °C, ^1^H-NMR (400 MHz, DMSO-*d*_6_), δ, ppm: 5.39 + 4.96 (s, 2H, NCH_2_, cis/trans amid conformer, %77/23), 7.28 (m, 4H, NH_2_ + ArH), 7.28 (m, 2H, ArH), 8.04 (s, 2H, Ar-H), 8.09 (s, 1H, ArH), 8.10 + 8.22 (s, 1H, N=CH, E/Z amid conformer, %75/25), 11.78 (s, 1H, NH). ^13^C-NMR (100 MHz, DMSO-*d*_6_), δ, ppm: 44.4 (NCH_2_), 116.4, 118.8, 129.6, 129.7, 131.0, 130.9 (Ar-C), 143.6 (N=CH), 150.4 (C=N), 152.8 (N=CH), 156.4 (C=N), 162.3 (C_F_), 168.6 (C=O). Elemental analysis: Calculated for C_14_H_12_FN_7_O, C, 52.99, H, 3.81, N, 30.90; found: C, 52.88, H, 3.77, N, 30.84.

2-(6-Amino-9*H*-purin-9-yl)-N′-(4-chlorobenzylidene)acetohydrazide (**3d**): Yield: 2.90 g (87%), m.p.: 320–322 °C, ^1^H-NMR (400 MHz, DMSO-*d*_6_), δ, ppm: 5.40 + 4.97 (s, 2H, NCH_2_, cis/trans amid conformer, %75/25), 7.28 (m, 2H, NH_2_), 7.52 (m, 2H, ArH), 7.72 (s, 2H, Ar-H), 7.78 (s, 2H, Ar-H), 8.03 (s, 1H, ArH), 8.11 + 8.21 (s, 1H, N=CH, E/Z amid conformer, %77/23), 11.83 (s, 1H, NH). ^13^C-NMR (100 MHz, DMSO-*d*_6_), δ, ppm: 44.4 (NCH_2_), 118.8, 129.1, 129.2, 129.4, 129.4, 133.3, 134.9 (Ar-C), 143.5 (N=CH), 150.4 (C=N), 152.9 (N=CH), 156.4 (C=N), 168.8 (C=O). Elemental analysis: Calculated for C_14_H_12_ClN_7_O, C, 49.79, H, 3.58, N, 29.05; found: C, 49.72, H, 3.53, N, 29.01.

2-(6-Amino-9*H*-purin-9-yl)-N′-(2-fluoro-4-chlorobenzylidene)acetohydrazide (**3e**): Yield: 2.85 g (81%), m.p.: 303–305 °C, ^1^H-NMR (400 MHz, DMSO-*d*_6_), δ, ppm: 5.40 + 4.97 (s, 2H, NCH_2_, cis/trans amid conformer, %80/20), 7.21(s, 2H, NH_2_), 7.38 (m, 1H, Ar-H), 7.57 (s, 1H, Ar-H), 7.98 (s, 1H, Ar-H), 8.09 (s, 2H, ArH), 8.19 + 8.38 (s, 1H, N=CH, E/Z amid conformer, %78/22), 11.92 (s, 1H, NH). ^13^C-NMR (100 MHz, DMSO-*d*_6_), δ, ppm: 44.4 (NCH_2_), 117.3, 118.7, 121.0, 121.2, 125.9, 128.2, 135.8 (Ar-C), 136.8 (N=CH), 150.4 (C=N), 152.9 (N=CH), 156.4 (C=N), 162.1 + 159.6 (C_F_, J = 273 Hz) 168.8 (C=O). Elemental analysis: Calculated for C_14_H_11_FClN_7_O, C, 47.99, H, 3.17, N, 27.98; found: C, 47.92, H, 3.11, N, 27.93.

2-(6-Amino-9*H*-purin-9-yl)-N′-(3-bromo-4-fluorobenzylidene)acetohydrazide (**3f**): Yield: 3.40 g (86%), m.p.: 292–294 °C, ^1^H-NMR (400 MHz, DMSO-*d*_6_), δ, ppm: 5.40 + 4.97 (s, 2H, NCH_2_, cis/trans amid conformer, %80/20), 7.21(s, 2H, NH_2_), 7.46 (m, 1H, Ar-H), 7.80 (s, 1H, Ar-H), 8.01 (s, 1H, Ar-H), 8.09 (s, 2H, ArH), 8.20 + 8.13 (s, 1H, N=CH, E/Z amid conformer, %76/24), 11.87 (s, 1H, NH). ^13^C-NMR (100 MHz, DMSO-*d*_6_), δ, ppm: 44.5 (NCH_2_), 109.2, 117.5, 117.6, 118.8, 129.0, 131.9, 132.5, 132.7 (Ar-C), 142.2 (N=CH), 150.4 (C=N), 152.9 (C=N), 156.9 (N=CH), 160.7 + 158.2 (C_F_, J = 247 Hz) 168.8 (C=O). Elemental analysis: Calculated for C_14_H_11_FBrN_7_O, C, 42.78, H, 2.82, N, 24.95; found: C, 42.72, H, 2.75, N, 24.90.

2-(6-Amino-9*H*-purin-9-yl)-N′-(3-methoxybenzylidene)acetohydrazide (**3g**): Yield: 3.00 g (85%), m.p.: 290–292 °C, ^1^H-NMR (400 MHz, DMSO-*d*_6_), δ, ppm: 5.40 + 4.97 (s, 2H, NCH_2_, cis/trans amid conformer, %73/27), 6.98 (s, 1H, Ar-H), 7.21(s, 2H, NH_2_), 7.28 (m, 2H, Ar-H), 7.35 (m, 3H, Ar-H), 8.01 (s, 1H, ArH), 8.19 + 8.10 (s, 1H, N=CH, E/Z amid conformer, %79/21), 11.78 (s, 1H, NH).^13^C-NMR (100 MHz, DMSO-*d*_6_), δ, ppm: 44.4 (NCH_2_), 55.6 (OCH_3_), 111.9, 116.5, 118.8, 120.1, 130.4, 135.7 (Ar-C), 144.6 (N=CH), 150.4 (Ar-C), 152.8, 156.4 (N=CH), 160.0 (C=N) 168.6 (C=O). Elemental analysis: Calculated for C_15_H_15_N_7_O_2_, C, 53.09, H, 4.46, N, 28.90; found: C, 53.02, H, 4.41, N, 28.85.

2-(6-Amino-9*H*-purin-9-yl)-N′-(4-methoxybenzylidene)acetohydrazide (**3h**): Yield: 3.08 g (88%), m.p.: 291–293 °C, ^1^H-NMR (400 MHz, DMSO-*d*_6_), δ, ppm: 5.37 + 4.94 (s, 2H, NCH_2_, cis/trans amid conformer, %76/24), 6.98 (s, 2H, Ar-H), 7.19(s, 2H, NH_2_), 7.28 (m, 2H, Ar-H), 7.69 (m, 2H, Ar-H), 7.99 (s, 1H, ArH), 8.16 + 8.09 (s, 1H, N=CH, E/Z amid conformer, %69/31), 11.64 (s, 1H, NH).^13^C-NMR (100 MHz, DMSO-*d*_6_), δ, ppm: 44.4 (NCH_2_), 55.8 (OCH_3_), 114.7, 114.8, 118.7, 126.6, 129.0 (Ar-C), 144.7 (N=CH), 150.4 (N=CH), 152.8 (N=CH), 156.4 (C=N), 161.3 (C=N) 168.3 (C=O). Elemental analysis: Calculated for C_15_H_15_N_7_O_2_, C, 53.09, H, 4.46, N, 28.90; found: C, 53.05, H, 4.44, N, 28.84.

2-(6-Amino-9*H*-purin-9-yl)-N′-(4-nitrobenzylidene)acetohydrazide (**3i**): Yield: 2.58 g (76%), m.p.: 311–313 °C, ^1^H-NMR (400 MHz, DMSO-*d*_6_), δ, ppm: 5.45 + 5.02 (s, 2H, NCH_2_, cis/trans amid conformer, %77/23), 7.24 (s, 2H, NH_2_), 7.99 (d, J = 8.0 Hz,2H, Ar-H), 8.14 (d, J = 8.0 Hz,2H, Ar-H), 8.25 m (2H, Ar-H), 8.27+8.37 (s, 1H, N=CH, E/Z amid conformer, %65/35), 12.07 (s, 1H, NH). ^13^C-NMR (100 MHz, DMSO-*d*_6_), δ, ppm: 44.3 (NCH_2_), 111.9, 124.5, 128.4, 140.6 (Ar-C), 142.5 (N=CH), 148.3, 150.4 (Ar-C), 152.9 (N=CH), 156.4, 169.1 (C=O). Elemental analysis: Calculated for C_14_H_12_N_8_O_3_, C, 49.41; H, 3.55; N, 32.93; found: C, 49.34; H, 3.50; N, 32.86.

2-(6-Amino-9*H*-purin-9-yl)-N′-(2,3-dimethoxybenzylidene)acetohydrazide (**3j**): Yield: 3.05 g (86%), m.p.: 299–301 °C, ^1^H-NMR (400 MHz, DMSO-*d*_6_), δ, ppm: 3.80 (s, 3H, OCH_3_), 3.86 (s, 3H, OCH_3_), 5.38 + 4.95 (s, 2H, NCH_2_, cis/trans amid conformer, %78/22), 7.12(s, 2H, NH_2_), 7.32 (m, 2H, Ar-H), 7.50 (m, 1H, Ar-H), 8.11 (s, 2H, ArH), 8.33 + 8.48 (s, 1H, N=CH, E/Z amid conformer, %77/23), 11.74 (s, 1H, NH). ^13^C-NMR (100 MHz, DMSO-*d*_6_), δ, ppm: 44.4 (NCH_2_), 55.2, 61.66 (OCH_3_), 114.7, 117.4, 118.8, 124.8, 127.7 (Ar-C), 140.6 (2N=CH), 148.4 (Ar-C), 150.4 (Ar-C), 152.9 (N=CH), 153.1 (Ar-C), 156.4 (Ar-C), 161.2 (C=N) 168.5 (C=O). Elemental analysis: Calculated for C_16_H_17_N_7_O_3_, C, 54.08, H, 4.82, N, 27.59; found: C, 54.02, H, 4.77, N, 27.52.

2-(6-Amino-9*H*-purin-9-yl)-N′-(2,4-dimethoxybenzylidene)acetohydrazide (**3k**): Yield: 2.87 g (80%), m.p.: 299–301 °C, ^1^H-NMR (400 MHz, DMSO-*d*_6_), δ, ppm: 3.80 (s, 3H, OCH_3_), 3.84 (s, 3H, OCH_3_), 5.35 + 4.91 (s, 2H, NCH_2_, cis/trans amid conformer, %79/21), 6.61 (s, 2H, Ar-H), 7.22 (m, 2H, NH_2_), 8.09 (s, 1H, Ar-H), 8.10 (s, 2H, ArH), 8.29 + 8.46 (s, 1H, N=CH, E/Z amid conformer, %78/22), 11.57 (s, 1H, NH). ^13^C-NMR (100 MHz, DMSO-*d*_6_), δ, ppm: 44.4 (NCH_2_), 55.9, 56.2 (OCH_3_), 98.7, 107.0, 115.2, 118.8, 127.7 (Ar-C), 140.6 143.5 (2N=CH), 150.4 (Ar-C), 152.8 (N=CH), 153.3, 159.6 (Ar-C), 163.1 (C=N) 168.2 (C=O). Elemental analysis: Calculated for C_16_H_17_N_7_O_3_, C, 54.08, H, 4.82, N, 27.59; found: C, 54.00, H, 4.71, N, 27.50.

2-(6-Amino-9H-purin-9-yl)-N′-(3,4,5-trimethoxybenzylidene)acetohydrazide (**3l**): Yield: 2.85 g (84%), m.p.: 295–298 °C, ^1^H-NMR (400 MHz, DMSO-*d*_6_), δ, ppm: 3.68 (s, 3H, OCH_3_), 3.82 (s, 6H, 2OCH_3_), 5.41 + 4.96 (s, 2H, NCH_2_, cis/trans amid conformer, %76/24), 6.99 (s, 1H, Ar-H), 7.05 (s, 1H, Ar-H), 7.21 (m, 2H, NH_2_), 7.96 (s, 1H, Ar-H), 8.09 (s, 1H, ArH), 8.10 + 8.14 (s, 1H, N=CH, E/Z amid conformer, %78/22), 11.78 (s, 1H, NH). ^13^C-NMR (100 MHz, DMSO-*d*_6_), δ, ppm: 44.6 (NCH_2_), 56.4 (2OCH_3_), 60.6 (OCH_3_), 104.8, 118.8, 129.4, 139.6 (Ar-C), 144.6 (2N=CH), 150.5 (Ar-C), 152.8 (Ar-C), 153.8 (N=CH), 156.4 (C=N) 168.6 (C=O). Elemental analysis: Calculated for C_17_H_19_N_7_O_4_, C, 52.98, H, 4.97, N, 24.44; found: C, 52.90, H, 4.94, N, 24.38.

2-(6-Amino-7*H*-purin-7-yl)-N′-(2-hydroxybenzylidene)acetohydrazide (**3m**): Yield: 2.30 g (74%), m.p.: 302–304 °C, ^1^H-NMR (400 MHz, DMSO-*d*_6_), δ, ppm: 5.37 + 4.98 (s, 2H, NCH_2_, cis/trans amid conformer, %62.5/37.5), 6.68 (m, 2H, Ar-H), 7.28 (m, 3H, Ar-H+NH_2_), 7.77 (s, 1H, Ar-H), 8.09 (s, 2H, Ar-H), 8.35 + 8.43 (s, 1H, N=CH, E/Z amid conformer, %73/27), 10.88+10.06 (s, 1H,OH), 11.69+12.06 (s, 1H, NH). ^13^C-NMR (100 MHz, DMSO-*d*_6_), δ, ppm: 44.4 (NCH_2_), 116.8, 118.7, 119.9, 120.5, 126.5, 129.5, 131.8 (Ar-C), 142.0, 148.0 (N=CH), 150.4 (Ar-C), 152.8 (N=CH), 156.9, 156.4 (Ar-C), 156.9 (C=N), 168.3 (C=O). Elemental analysis: Calculated for C_14_H_13_N_7_O_2_, C, 54.02, H, 4.21, N, 31.50; found: C, 53.94, H, 4.14, N, 31.43.

2-(6-Amino-7*H*-purin-7-yl)-N′-(5-bromo-2-hydroxybenzylidene)acetohydrazide (**3n**): Yield: 2.70 g (69%), m.p.: 317–319 °C, ^1^H-NMR (400 MHz, DMSO-*d*_6_), δ, ppm: 5.40 + 4.99 (s, 2H, NCH_2_, cis/trans amid conformer, %64/36), 6.85 (s, 1H, Ar-H), 7.22 (m, 2H, NH_2_), 7.37 (s, 1H, Ar-H), 7.90 (s, 1H, Ar-H), 8.08 (s, 2H, Ar-H), 8.28 + 8.40 (s, 1H, N=CH, E/Z amid conformer, %65/35), 10.40 (s, 1H, OH), 11.76 (s, 1H, NH). ^13^C-NMR (100 MHz, DMSO-*d*_6_), δ, ppm: 44.5 (NCH_2_), 111.3, 118.8, 118.9, 119.1, 123.0, 128.1, 134.2 (Ar-C), 140.0, 145.5, 152.8 (N=CH), 156.4 (C=N), 168.6 + 163.7 (C=O). Elemental analysis: Calculated for C_14_H_12_BrN_7_O_2_, C, 43.09, H, 3.10, N, 25.13; found: C, 43.02, H, 3.01, N, 25.05.

2-(6-Amino-7*H*-purin-7-yl)-N′-(5-chloro-2-hydroxybenzylidene)acetohydrazide (**3o**): Yield: 2.52 g (73%), m.p.: 306–308 °C, ^1^H-NMR (400 MHz, DMSO-*d*_6_), δ, ppm: 5.97 + 4.98 (s, 2H, NCH_2_, cis/trans amid conformer, %68/32), 6.90 (s, 1H, Ar-H), 7.22 (m, 3H, NH_2_ +Ar-H), 7.62 (s, 1H, Ar-H), 8.00 (s, 2H, Ar-H), 8.26 + 8.40 (s, 1H, N=CH, E/Z amid conformer, %65/35), 10.37 (s, 1H, OH), 11.76 (s, 1H, NH). ^13^C-NMR (100 MHz, DMSO-*d*_6_), δ, ppm: 44.5 (NCH_2_), 118.4, 122.4, 123.8, 125.2, 131.2 (Ar-C), 140.0, 145.7 (N=CH), 150.4 (Ar-C), 152.8 (N=CH), 155.8 (Ar-C), 158.3 (C=N), 168.6 (C=O). Elemental analysis: Calculated for C_14_H_12_ClN_7_O_2_, C, 48.63, H, 3.50, N, 28.36; found: C, 48.57, H, 3.44, N, 28.32.

2-(6-Amino-9*H*-purin-9-yl)-N′-(2-hydroxy-5-methylbenzylidene)acetohydrazide (**3p**): Yield: 2.40 g (74%), m.p.: 300–302 °C, ^1^H-NMR (400 MHz, DMSO-*d*_6_), δ, ppm: 3.80 (s, 3H, CH_3_), 5.37 + 4.99 (s, 2H, NCH_2_, cis/trans amid conformer, %68/32), 6.80 (s, 1H, Ar-H), 7.00 (s, 1H, Ar-H), 7.17 (m, 2H, NH_2_), 7.38 (s, 1H, Ar-H), 8.10 (s, 2H, Ar-H), 8.39 + 8.45 (s, 1H, N=CH, E/Z amid conformer, %58/42), 9.36 + 10.35 (s, 1H, OH), 11.71+12.07 (s, 1H, NH). ^13^C-NMR (100 MHz, DMSO-*d*_6_), δ, ppm: 20.5 (CH_3_), 44.4 (NCH_2_), 116.5, 118.7, 120.1, 126.4, 128.4, 129.4 (Ar-C), 132.7, 142.1, 148.0 (N=CH), 150.4 (Ar-C), 156.4 (C=N), 168.3 + 163.4 (C=O). Elemental analysis: Calculated for C_15_H_15_N_7_O_2_, C, 55.38; H, 4.65; N, 30.14; found: C, 55.31; H, 4.60; N, 30.09.

2-(6-Amino-9*H*-purin-9-yl)-N′-(2-hydroxy-3-methoxybenzylidene)acetohydrazide (**3q**): Yield: 2.54 g (74%), m.p.: 302–304 °C, ^1^H-NMR (400 MHz, DMSO-*d*_6_), δ, ppm: 3.80 (s, 3H, OCH_3_), 5.37 + 4.99 (s, 2H, NCH_2_, cis/trans amid conformer, %77/23), 6.81 (s, 1H, Ar-H), 6.98 (a, 1H, Ar-H), 7.23 (m, 2H, NH_2_), 7.38 (s, 2H, Ar-H), 8.11 (s, 2H, Ar-H), 8.39 + 8.45 (s, 1H, N=CH, E/Z amid conformer, %65/35), 9.36+10.54 (s, 1H, OH), 11.71 + 12.05 (s, 1H, NH). ^13^C-NMR (100 MHz, DMSO-*d*_6_), δ, ppm: 44.5 (NCH_2_), 55.7 (OCH_3_), 111.9, 116.6, 118.8, 120.14, 130.4 (N=CH), 135.7 (Ar-C), 144.6 (N=CH), 150.4 (Ar-C), 152.9 (N=CH), 156.4 (Ar-C), 160.0 (C=N), 168.7 (C=O). Elemental analysis: Calculated for C_15_H_15_N_7_O_3_, C, 52.78; H, 4.43; N, 28.73; found: C, 52.71; H, 4.36; N, 28.65.

2-(6-Amino-9*H*-purin-9-yl)-N′-(4-(diethylamino)-2-hydroxybenzylidene)acetohydrazide (**3r**): Yield: 3.17 g (83%), m.p.: 305–307 °C, ^1^H-NMR (400 MHz, DMSO-*d*_6_), δ, ppm: 1.06 (t, J = 6.0 Hz, 6H, 2CH_3_), 3.32 (m, 4H, 2CH_2_), 5.30 + 4.94 (s, 2H, NCH_2_, cis/trans amid conformer, %45/55), 6.06 (s, 1H, Ar-H), 6.24 (s,1H, Ar-H), 7.22 (s, 2H, NH_2_), 7.45 (m, 1H, Ar-H), 8.10 (m, 2H, Ar-H), 8.15+8.21 (s, 1H, N=CH, E/Z amid conformer, %55/45), 9.78+11.05 (OH), 11.40 + 11.77 (s, 1H, NH). ^13^C-NMR (100 MHz, DMSO-*d*_6_), δ, ppm: 13.0 (2CH_3_), 44.2 (2CH_2_), 44.3 (NCH_2_), 97.8, 104.1, 106.6, 107.6, 118.7, 131.9 (Ar-C), 144.3 (N=CH), 149.9 (Ar-C), 150.7 152.8 (N=CH), 156.4, 158.7 + 160.0 (C=N), 162.6 + 167.3 (C=O). Elemental analysis: Calculated for C_18_H_22_N_8_O_2_, C, 56.53; H, 5.80; N, 29.30; found: C, 56.46; H, 5.73; N, 29.25.

### 3.2. Cytotoxicity

#### 3.2.1. Cell Viability

Cell viability assays were performed using the human lung adenocarcinoma cell line A549 (ATCC^®^ CCL-185™) and the normal human lung fibroblast cell line MRC-5 (ATCC^®^ CCL-171™). Both cell lines were obtained from the American Type Culture Collection (ATCC, Manassas, VA, USA). The cells were maintained in Dulbec-co’s modified Eagle’s medium (DMEM), supplemented with 10% fetal bovine serum (FBS), 2 mM L-glutamine, 100 U/mL penicillin, and 100 mg/L streptomycin (Biochrom AG, Berlin, Germany) at 37 °C in a humidified atmosphere of 5% CO_2_.

The cytotoxic effect was determined by the MTT (3-(4,5-dimethyl-2-thiazolyl)-2,5-diphenyl-2H-tetrazolium bromide) cell viability as-say. Cells were seeded at an initial concentration of 1 × 10^5^ cells/mL in 96-well micro-plates. Cells were then treated with different concentrations (0.5, 5, 10, 25, 50, 100 and 250 µM) of the novel synthesized compounds for 48 h. After the incubation period, the formed formazan crystals were dissolved in dimethyl sulfoxide (DMSO) and the opti-cal density (OD) of compounds was measured at 570 nm using a spectrophotometer (BMG Labtech, Ortenberg, Germany). The compounds were initially prepared as stock solutions in DMSO and subsequently diluted with culture medium before treatment. The final DMSO concentration in all wells did not exceed 0.1% (*v*/*v*). A vehicle control containing the same concentration of DMSO without compounds was included in each experiment, and this concentration showed no significant effect on cell viability. Cytotoxicity was expressed as an increase in the mean percentage of cytotoxicity relative to the unexposed control ± standard deviation (SD). Doxorubicin (0.5, 5 and 50 µM) was used as a positive control. Control values were set at 0% cytotoxicity. IC50 was calculated by fitting the data to a sigmoidal curve and using a four-parameter logistic model and presented as an average of three independent measurements. The IC_50_ value was reported at a 95% confidence interval and the calculation was performed using GraphPad Prism software 9.5.1 (San Diego, CA, USA). The values of the blank wells were subtracted from each well of treated and control cells and inhibition of growth of 50% was calculated compared to untreated controls. Briefly, IC_50_ values were calculated from full dose–response curves obtained using multiple concentrations, and these IC_50_ concentrations were subsequently used for all mechanistic assays. Doxorubicin was used as a positive control. Each sample was tested in triplicate.

#### 3.2.2. LDH Assay

LDH leakage assay was determined using the LDH Assay Kit (Cat no. ab102526, Abcam, Cambridge, UK) on the culture medium of a new set (at a density of 1 × 10^5^ cells/well) of A549 cells exposed to calculated IC50 values of the compounds for 48 h. 100 μL of culture medium was transferred to a new 96 well plate. 100 μL of LDH reaction solution was added to each well, and absorbance was measured at 490 nm using an ELISA plate reader (BMG Labtech, Ortenberg, Germany) after 30 min.

#### 3.2.3. TOS Levels

TOS were evaluated using a commercially available kit (Rel Assay Diagnostics^®^, Gaziantep, Turkey) according to the manufacturer’s instructions. A549 cells exposed to calculated IC50 values of the compounds for 48 h. The TOS assay quantifies oxidizing agents based on their ability to convert an iron (II) ion chelator complex to iron (III) ions. The results were calibrated against hydrogen peroxide (H_2_O_2_, 250 µM) as a positive control for 48 h and expressed as µmol H_2_O_2_ equivalents per liter. The analysis was performed in three independent biological replicates (n = 3).

#### 3.2.4. Determination of Intracellular GSH/GSSG Ratio

Intracellular reduced (GSH) and oxidized (GSSG) glutathione levels were measured using the GSH/GSSG Ratio Detection Assay Kit (Cat. No: 703002, Cayman Chemical, Ann Arbor, MI, USA) according to the manufacturer’s protocol. Briefly, A549 cells were seeded in 6-well plates at a density of 1 × 10^5^ cells/well and treated with the IC_50_ concentrations of test compounds for 48 h. After treatment, cells were washed with cold PBS and lysed with MES buffer provided in the kit.

To prevent oxidation of GSH to GSSG during processing, samples were deproteinized using the supplied metaphosphoric acid solution and neutralized accordingly. Total glutathione and GSSG levels were measured separately by adding the appropriate buffers and reagents to the wells of a 96-well plate, followed by the addition of DTNB recycling reagent and glutathione reductase. Absorbance was read at 405 nm using a microplate reader (BMG Labtech, Ortenberg, Germany). The amount of GSH was calculated by subtracting 2×GSSG from the total glutathione, and the GSH/GSSG ratio was used to assess intracellular redox status.

#### 3.2.5. Intracellular Reactive Oxygen Species (iROS)

Intracellular ROS generation was assessed using 2′,7′-dichlorodihydrofluorescein diacetate (DCFH-DA). Cells were seeded in 6-well plates (1 × 10^5^ cells/well) and allowed to attach for 24 h. Cells were then treated with the IC_50_ concentrations of the test compounds for 48 h. After treatment, cells were washed twice with warm PBS and incubated with 10 µM DCFH-DA in serum-free medium for 30 min at 37 °C in the dark. Following dye loading, cells were washed, gently detached using enzyme-free dissociation buffer (Gibco, Thermo Fisher Scientific, Waltham, MA, USA), resuspended in ice-cold PBS, and immediately analyzed by flow cytometry (488 nm excitation; 530/30 nm emission, FITC channel). At least 10,000 events were acquired per sample after debris exclusion and singlet gating. ROS levels were quantified as median fluorescence intensity (MFI) and expressed as fold change relative to the control group (control = 1.0). Experiments were performed in three independent biological replicates (n = 3)

#### 3.2.6. Measurement of Mitochondrial Membrane Potential (MMP, ΔΨm)

Mitochondrial membrane potential (ΔΨm) was evaluated using the JC-1 dye (Cat. No. HY-K0601, MedChemExpress, Monmouth Junction, NJ, USA) according to the manufacturer’s instructions. Cells were seeded in 96-well plates (1 × 10^5^ cells/well) and treated with IC_50_ concentrations of the test compounds for 48 h. After treatment, cells were incubated with JC-1 staining solution at 37 °C for 20 min in the dark, washed with assay buffer, and fluorescence was measured using a microplate reader. Changes in ΔΨm were assessed by calculating the red/green fluorescence ratio (JC-1 aggregates/monomers). All experiments were conducted in triplicate biological repeats (n = 3)

#### 3.2.7. Cell Cycle Analysis

Cell cycle distribution was analyzed by propidium iodide (PI) staining and flow cytometry. A549 cells were seeded in 6-well plates at a density of 1 × 10^5^ cells per well and allowed to attach overnight. Cells were then treated with the IC_50_ concentrations of the selected compounds for 48 h. After treatment, both adherent and floating cells were collected, washed twice with cold phosphate-buffered saline (PBS, Gibco, Thermo Fisher Scientific, Waltham, MA, USA), and fixed overnight in 70% ice-cold ethanol at 4 °C. Following fixation, cells were centrifuged and resuspended in 500 µL PI/RNase staining solution containing 50 µg/mL propidium iodide and 100 µg/mL RNase A (Sigma-Aldrich, St. Louis, MO, USA). Samples were incubated in the dark for 30 min at 37 °C. Stained cells were analyzed using a Muse™ Cell Analyzer (Merck Millipore, Darmstadt, Germany). For each sample, at least 10,000 events were recorded. DNA content histograms were generated using the Muse™ Cell Cycle Analysis software module, and cell populations were quantified as percentages of G0/G1, S, and G2/M phases. All experiments were performed in three independent biological replicates (n = 3), and data were expressed as mean ± SD.

#### 3.2.8. Apoptosis Analysis (Annexin V–FITC/PI Flow Cytometry)

Apoptotic cell death was evaluated using the Muse™ Annexin V & Dead Cell Kit (Cat. No. MCH100105, Merck Millipore, Burlington, MA, USA) according to the manufacturer’s protocol. A549 cells were seeded in 6-well plates at a density of 4 × 10^5^ cells/well and allowed to attach overnight. Cells were then exposed to the IC_50_ concentrations of the selected compounds for 48 h.

Following treatment, both floating and adherent cells were collected, centrifuged (1000× *g*, 10 min, 4 °C), and washed with phosphate-buffered saline (PBS). Cell pellets were resuspended in culture medium, and 100 µL of the cell suspension was mixed with 100 µL of Annexin V reagent. Samples were incubated for 20 min at room temperature in the dark and analyzed using a Muse™ Cell Analyzer (Merck Millipore, Billerica, MA, USA).

Prior to Annexin V/PI analysis, cell debris and doublets were excluded based on FSC/SSC and FSC-A/FSC-H gating. For each sample, at least 10,000 events were recorded. Cell populations were automatically classified as viable (Annexin V^−^/PI^−^), early apoptotic (Annexin V^+^/PI^−^), late apoptotic (Annexin V^+^/PI^+^), and necrotic (Annexin V^−^/PI^+^). Results were expressed as mean ± standard deviation (SD) of three independent experiments (n = 3).

#### 3.2.9. Statistical Analysis

Statistical analysis was performed using SPSS 20.0 (SPSS, Chicago, IL, USA). Differences between groups were assessed using one-way analysis of variance (ANOVA) followed by Tukey post hoc test for multiple comparisons. All experiments were performed in three independent biological replicates conducted on different days using separately prepared cell cultures. Data are expressed as mean ± standard deviation (SD) obtained from these independent experiments. A *p*-value less than 0.05 (≤0.05) was considered statistically significant.

### 3.3. The Protocol of Molecular Docking Studies

Molecular docking simulations were performed to investigate the binding modes and interaction patterns of the synthesized adenine–hydrazone derivatives (**3a**–**3r**) within the active sites of VEGFR2, ALK5 (TGF-betaR1), and EGFR. All computational calculations were carried out using the Schrödinger Maestro molecular modeling suite (Release [2018-4], Schrödinger, LLC, New York, NY, USA).

#### 3.3.1. Protein Preparation

The high-resolution crystal structures of the target proteins were retrieved from the RCSB Protein Data Bank (PDB). The following PDB entries were selected based on their resolution and relevance to the study: VEGFR2 (PDB ID: 4ASD), ALK5 (PDB ID: 3HMM), and EGFR (PDB ID: 6V6O) [58,59,60]. The PDB files were processed using the Protein Preparation Wizard tool. Initially, all crystallographic water molecules (beyond 5 Å from the hetero group), co-factors, and metal ions not essential for catalytic activity were removed. Bond orders were assigned, and missing hydrogen atoms were added. The protonation states of ionizable residues were predicted using Epik at a physiological pH. Finally, a restrained energy minimization was performed using the OPLS4 force field to relieve steric clashes, with the convergence criterion set to a root-mean-square deviation (RMSD) of 0.30 Å for heavy atoms.

#### 3.3.2. Ligand Preparation

The 2D structures of the synthesized compounds (**3a**–**r**) were sketched using the 2D Sketcher tool in Maestro and converted to 3D geometries. The LigPrep module was employed to prepare the ligands for docking. The ionization states and tautomeric forms were generated at physiological pH using Epik, and the lowest energy conformers were determined. The geometry of each ligand was minimized using the OPLS4 force field to obtain the energetically most stable conformations.

#### 3.3.3. Induced Fit Docking (IFD) Protocol

To account for the conformational flexibility of the protein active site and to capture potential structural rearrangements upon ligand binding, the Induced Fit Docking (IFD) protocol combined with Glide XP was employed. This protocol combines the docking capabilities of Glide with the protein structure prediction features of Prime to generate an ensemble of diverse receptor conformations. The 2D and 3D ligand-protein interaction diagrams were generated and analyzed using the Ligand Interaction Diagram tool in Maestro. The active site for each target was defined by a cubic box centered on the centroid of the co-crystallized native inhibitor within the respective PDB structure (Sorafenib for 4ASD, the co-crystal ligand for 3HMM, and the inhibitor for 6V6O). The scaling factor for van der Waals radii was set to 1.0 with a partial charge cutoff of 0.25 to accommodate the ligands within the binding pocket.

#### 3.3.4. Validation of Docking Protocol

The docking protocol was validated by redocking the co-crystallized ligand into the active site of the receptor. The superposition of the best-docked pose with the experimental structure resulted in an RMSD value of less than 2.0 Å, confirming the robustness and reproducibility of the docking parameters.

## 4. Conclusions

In this study, a series of adenine–hydrazone hybrid derivatives (**3a**–**r**) were synthesized and structurally characterized, and their biological effects were investigated in A549 human lung adenocarcinoma cells together with non-malignant MRC-5 lung fibroblasts.

Biological evaluation showed that several derivatives produced reproducible cytotoxic responses in A549 cells, with IC_50_ values ranging from 51.1 ± 1.9 to 96.9 ± 4.5 µM. Compounds bearing electron-withdrawing substituents, particularly the para-nitro derivative **3i** and the multi-halogenated analog **3f**, exhibited relatively higher activity within the synthesized series. Structure–activity relationship analysis indicated that the electronic properties and positional distribution of aromatic substituents influence the observed cellular responses, with electron-withdrawing substituents generally associated with stronger activity and electron-donating groups with weaker effects.

In addition, methoxy substitution patterns significantly influence biological performance. While mono-methoxy derivatives showed limited activity, di- and trimethoxy-substituted compounds, particularly **3l**, displayed improved cytotoxicity and selectivity, highlighting the positive contribution of increased methoxy substitution. Furthermore, incorporation of salicylaldehyde-derived moieties affected biological activity, with compound **3r** exhibiting enhanced potency compared to other salicylaldehyde analogs.

A general trend toward enhanced activity was observed for compounds containing electron-withdrawing substituents; however, the activity of compound **3r** demonstrates that strong donor substituents capable of inducing donor–acceptor interactions and improving physicochemical properties can also substantially contribute to biological efficacy.

Biochemical and cellular analyses demonstrated that the reduction in cell viability was accompanied by membrane damage, increased intracellular oxidant levels, depletion of glutathione, and elevated reactive oxygen species production. Mitochondrial membrane potential measurements revealed significant mitochondrial depolarization following compound treatment. The extent of mitochondrial dysfunction closely paralleled oxidative stress parameters, suggesting a functional relationship between intracellular redox imbalance and mitochondrial impairment. Flow cytometric analysis further showed a marked increase in Annexin V-positive cell populations, indicating that the predominant mode of cell death was regulated rather than necrotic. Collectively, the findings indicate that the cytotoxic activity of the synthesized adenine–hydrazone derivatives is associated with oxidative stress-related mitochondrial dysfunction and apoptosis-like cell death in A549 cells. The results further demonstrate that substituent-dependent electronic properties of the aromatic ring influence not only cytotoxic potency but also the extent of oxidative and mitochondrial responses. These observations provide a mechanistic framework for understanding the biological activity of this compound class and may guide the rational design of future derivatives with improved biological profiles.

The molecular docking simulations provide compelling structural evidence that the synthesized adenine–hydrazone derivatives exert their antiproliferative effects through a multi-target mechanism, primarily involving the simultaneous inhibition of EGFR, VEGFR2 and ALK5 kinase domains. The in silico analyses successfully rationalize the experimental findings, revealing that the superior potency of compound **3i** is driven by its strong affinity for the ALK5 active site, while the selectivity of compound **3l** is attributed to its distinct binding mode within the VEGFR2 pocket. Collectively, these results identify the adenine–hydrazone scaffold as a promising lead structure for the development of novel multi-kinase inhibitors targeting non-small-cell lung cancer.

Overall, the results demonstrate that adenine–hydrazone hybridization represents a promising strategy for the development of novel anticancer agents. Although the synthesized compounds were less potent than the reference drug doxorubicin, several derivatives showed improved selectivity toward cancer cells, suggesting reduced toxicity to normal cells. These findings provide a strong foundation for further structural optimization and mechanistic studies, and the most promising compounds may serve as valuable lead candidates for the development of new anticancer therapeutics targeting lung cancer.

## Figures and Tables

**Table 1:** IC_50_ (µM) values and selective index (SI) of the synthesized compounds on A549 Lung Cancer cells and MR5 Lung fibroplast cells.

Compound	A549 Lung Cancer IC_50_ Values (µM ± SD)	MR5 Lung Fibroplast IC_50_ Values (µM ± SD)	SI = IC_50_ (MR5)/IC_50_ (A549)
**3a**	96.9 ± 4.5	92.2 ± 3.9	0.95
**3b**	75.5 ± 2.7	112.6 ± 4.2	1.49
**3c**	68.3 ± 2.7	111.3 ± 4.9	1.63
**3d**	72.3 ± 3.1	87.9 ± 3.7	1.22
**3e**	74.9 ± 3.3	95.7 ± 4.6	1.28
**3f**	58.8 ± 2.4	87.2 ± 3.8	1.48
**3g**	75.8 ± 3.3	99.2 ± 4.1	1.31
**3h**	96.3 ± 4.2	88.9 ± 3.8	0.92
**3i**	51.1 ± 1.9	57.1 ± 2	1.12
**3j**	54.4 ± 2.3	49.5 ± 1.4	0.91
**3k**	69.8 ± 2.8	93.2 ± 3.9	1.34
**3l**	65.8 ± 2.6	112.8 ± 4.9	1.71
**3m**	69 ± 2.8	93 ± 3.9	1.35
**3n**	75.3 ± 3.1	127.5 ± 5.1	1.69
**3o**	96 ± 4.4	112.6 ± 4.8	1.17
**3p**	84.7 ± 3.7	85.1 ± 3.5	1.00
**3q**	83.5 ± 3.7	118.3 ± 4.8	1.42
**3r**	55. 9 ± 2	54.8 ± 2.1	0.98
**Doxorubicin**	8.6 ± 1.2	7.4 ± 0.9	0.86

**Table 2:** Effects of selected compounds on LDH release, TOS levels, and intracellular GSH/GSSG ratio in A549 cells (48 h, IC_50_).

Treatment	LDH (% of Control)	TOS (µM H_2_O_2_ Eq/L)	GSH/GSSG
Control	100 ± 5	12.0 ± 1.0	10.0 ± 0.7
**3c**	138 ± 7 *	20.0 ± 1.3 *	7.0 ± 0.5 *
**3l**	135 ± 7 *	18.8 ± 1.2 *	7.4 ± 0.5 *
**3f**	147 ± 8 **	23.5 ± 1.5 **	6.1 ± 0.4 **
**3i**	163 ± 9 ***	25.0 ± 1.6 ***	5.8 ± 0.4 ***
**3r**	159 ± 8 ***	26.5 ± 1.8 ***	5.4 ± 0.4 ***

Values are expressed as mean ± SD (n = 3). * *p* < 0.05, ** *p* < 0.01, *** *p* < 0.001 vs. control (one-way ANOVA followed by Tukey’s post hoc test).

**Table 3:** The docking scores of synthesized compounds against selected target in kcal/mol.

Compound	VEGFR2	EGFR	ALK5
**3a**	−11.879	−8.922	−11.301
**3b**	−10.559	−8.598	−9.88
**3c**	−12.32	−9.15	−11.463
**3d**	−11.957	−9.764	−11.836
**3e**	−11.934	−9.842	−11.045
**3f**	−11.753	−10.36	−10.687
**3g**	−11.48	−8.524	−10.406
**3h**	−12.104	−8.518	−10.777
**3i**	−11.305	−8.135	−10.985
**3j**	−11.583	−9.364	−9.236
**3k**	−12.092	−7.597	−10.384
**3l**	−12.128	−8.33	−8.304
**3m**	−12.486	−9.072	−11.959
**3n**	−11.927	−9.524	−11.707
**3o**	−11.397	−9.959	−10.63
**3p**	−11.005	−8.241	−10.353
**3q**	−11.383	−10.259	−11.286
**3r**	−11.828	−8.999	−11.284
**Ref.**	−12.065 ^a^	−10.633 ^b^	−11.611 ^c^

The superscripts ^a^ Sorafenib, ^b^ LN2380, and ^c^ GW855857 (TGFBR1-IN-2) represent the reference co-crystallized ligands for VEGFR2, EGFR, and ALK5, respectively.

## Data Availability

The original contributions presented in this study are included in the article/Supplementary Material. Further inquiries can be directed to the corresponding author.

## Supplementary Materials

The following supporting information can be downloaded at: https://www.mdpi.com/article/10.3390/ph19030474/s1, Figure S1. ^1^H-NMR spectra of compound 2 (DMSO-*d*_6_); Figure S2. ^13^C-NMR spectra of compound 2 (DMSO-*d*_6_); Figure S3. ^1^H-NMR spectra of compound **3a** (DMSO-*d*_6_); Figure S4. ^13^C-NMR spectra of compound **3a** (DMSO-*d*_6_); Figure S5. ^1^H-NMR spectra of compound **3b** (DMSO-*d*_6_); Figure S6. ^13^C-NMR spectra of compound **3b** (DMSO-*d*_6_); Figure S7. ^1^H-NMR spectra of compound **3c** (DMSO-*d*_6_); Figure S8. ^13^C-NMR spectra of compound **3c** (DMSO-*d*_6_); Figure S9. ^1^H-NMR spectra of compound **3d** (DMSO-*d*_6_); Figure S10. ^13^C-NMR spectra of compound **3d** (DMSO-*d*_6_); Figure S11. ^1^H-NMR spectra of compound **3e** (DMSO-*d*_6_); Figure S12. ^13^C-NMR spectra of compound **3e** (DMSO-*d*_6_); Figure S13. ^1^H-NMR spectra of compound **3f** (DMSO-*d*_6_); Figure S14. ^13^C-NMR spectra of compound **3f** (DMSO-*d*_6_); Figure S15. ^1^H-NMR spectra of compound **3g** (DMSO-*d*_6_); Figure S16. ^13^C-NMR spectra of compound **3g** (DMSO-*d*_6_); Figure S17. ^1^H-NMR spectra of compound **3h** (DMSO-*d*_6_); Figure S18. ^13^C-NMR spectra of compound **3h** (DMSO-*d*_6_); Figure S19. ^1^H-NMR spectra of compound **3i** (DMSO-*d*_6_); Figure S20. ^13^C-NMR spectra of compound **3i** (DMSO-*d*_6_); Figure S21. ^1^H-NMR spectra of compound **3j** (DMSO-*d*_6_); Figure S22. ^13^C-NMR spectra of compound **3j** (DMSO-*d*_6_); Figure S23. ^1^H-NMR spectra of compound **3k** (DMSO-*d*_6_); Figure S24. ^13^C-NMR spectra of compound **3k** (DMSO-*d*_6_); Figure S25. ^1^H-NMR spectra of compound **3l** (DMSO-*d*_6_); Figure S26. ^13^C-NMR spectra of compound **3l** (DMSO-*d*_6_); Figure S27. ^1^H-NMR spectra of compound **3m** (DMSO-*d*_6_); Figure S28. ^13^C-NMR spectra of compound **3m** (DMSO-*d*_6_); Figure S29. ^1^H-NMR spectra of compound **3n** (DMSO-*d*_6_); Figure S30. 13C-NMR spectra of compound **3n** (DMSO-d6); Figure S31. ^1^H-NMR spectra of compound **3o** (DMSO-*d*_6_); Figure S32. ^13^C-NMR spectra of compound **3o** (DMSO-*d*_6_); Figure S33. ^1^H-NMR spectra of compound **3p** (DMSO-*d*_6_); Figure S34. ^13^C-NMR spectra of compound **3p** (DMSO-*d*_6_); Figure S35. ^1^H-NMR spectra of compound **3q** (DMSO-*d*_6_); Figure S36. ^13^C-NMR spectra of compound **3q** (DMSO-*d*_6_); Figure S37. ^1^H-NMR spectra of compound **3r** (DMSO-*d*_6_); Figure S38. ^13^C-NMR spectra of compound **3r** (DMSO-*d*_6_). Figure S39. 2D-NMR (HMBC) spectra of compound **2a**.
