# Supplementary material for: Novel Adenine–Hydrazone Hybrids Against Human Lung Adenocarcinoma (A549): Design, Synthesis, Cellular Mechanistic Investigation and Molecular Docking Studies
Source: Pharmaceuticals (Basel). 2026 Mar 13;19(3):474. doi: 10.3390/ph19030474 (PMC13028652; doi:10.3390/ph19030474)
Supplement: Supplementary file 1 [file pharmaceuticals-19-00474-s001.zip › pharmaceuticals-4181702-supplementary.pdf]

*Article*

# Novel Adenine–Hydrazone Hybrids Against Human Lung Adenocarcinoma (A549): Design, Synthesis, Cellular Mechanistic Investigation and Molecular Docking Studies

**Emre Menteşe [1], Nedime Çalışkan [1], Didem Aksu [2], Mustafa Emirik [1], Adem Güner [3] and Fatih Yılmaz [4,*]**

[1] Department of Chemistry, Faculty of Art and Sciences, Recep Tayyip Erdogan University,
53100 Rize, Turkey; emre.mentese@erdogan.edu.tr (E.M.);
nedime_caliskan19@erdogan.edu.tr (N.Ç.);
mustafa.emirik@erdogan.edu.tr (M.E.)
[2] Central Research Testing and Analysis Laboratory Research and Application Center, Ege University,35100 Izmir, Turkey; didem.eroglu@ege.edu.tr
[3] Department of Occupational Health and Safety, Faculty of Health Sciences, Sinop University,
57000 Sinop, Turkey; ademguner@sinop.edu.tr
[4] Department of Chemistry and Chemical Process Technology, Vocational School of Technical Sciences,
Recep Tayyip Erdogan University, 53100 Rize, Turkey
* Correspondence: fyilmaz@erdogan.edu.tr

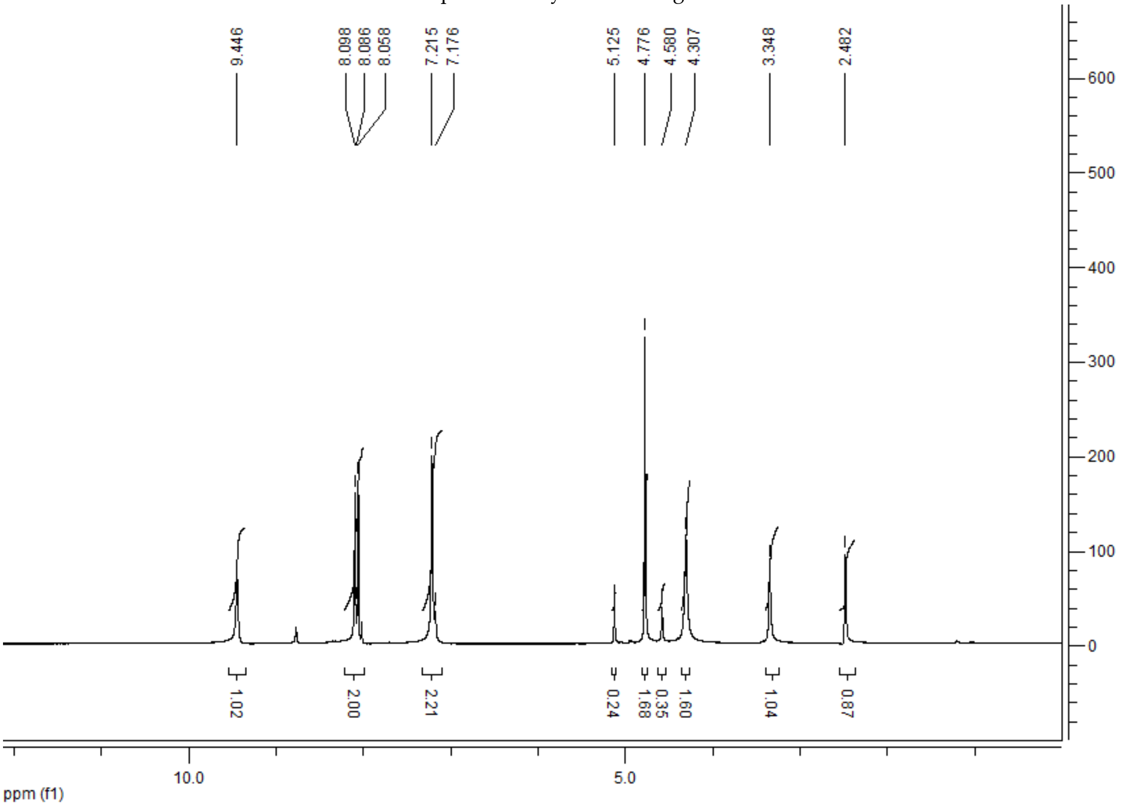


**Figure S1.** $^{1}$H-NMR spectra of compound **2** (DMSO-*d*$_6$)

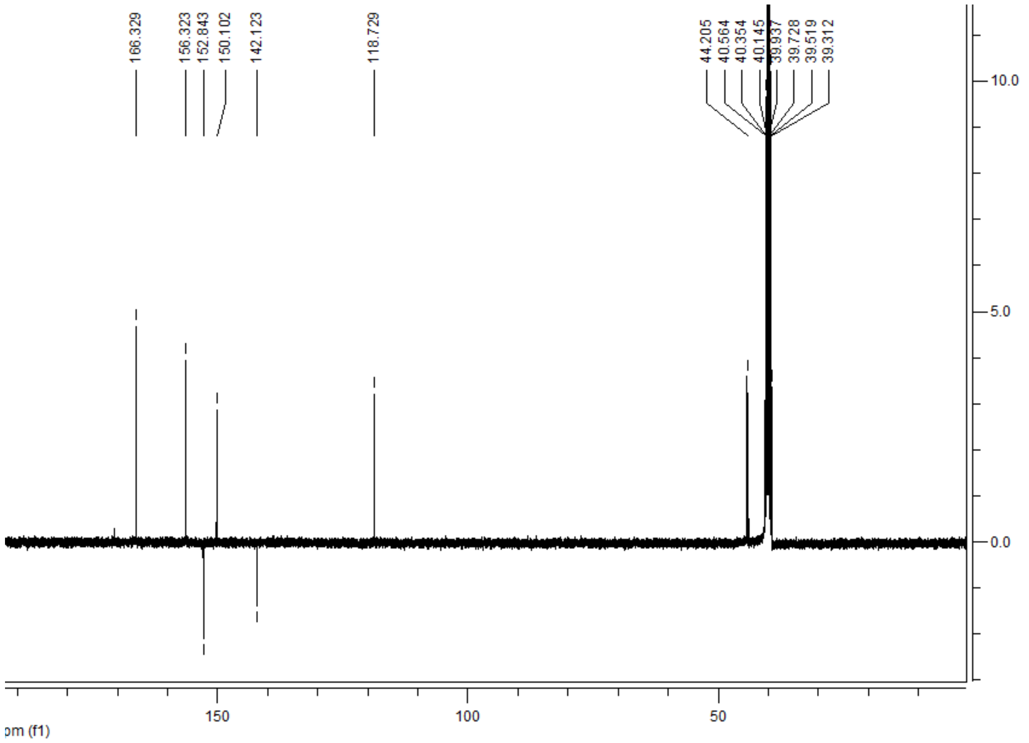


**Figure S2.** $^{13}$C-NMR spectra of compound **2** (DMSO-$d_6$).

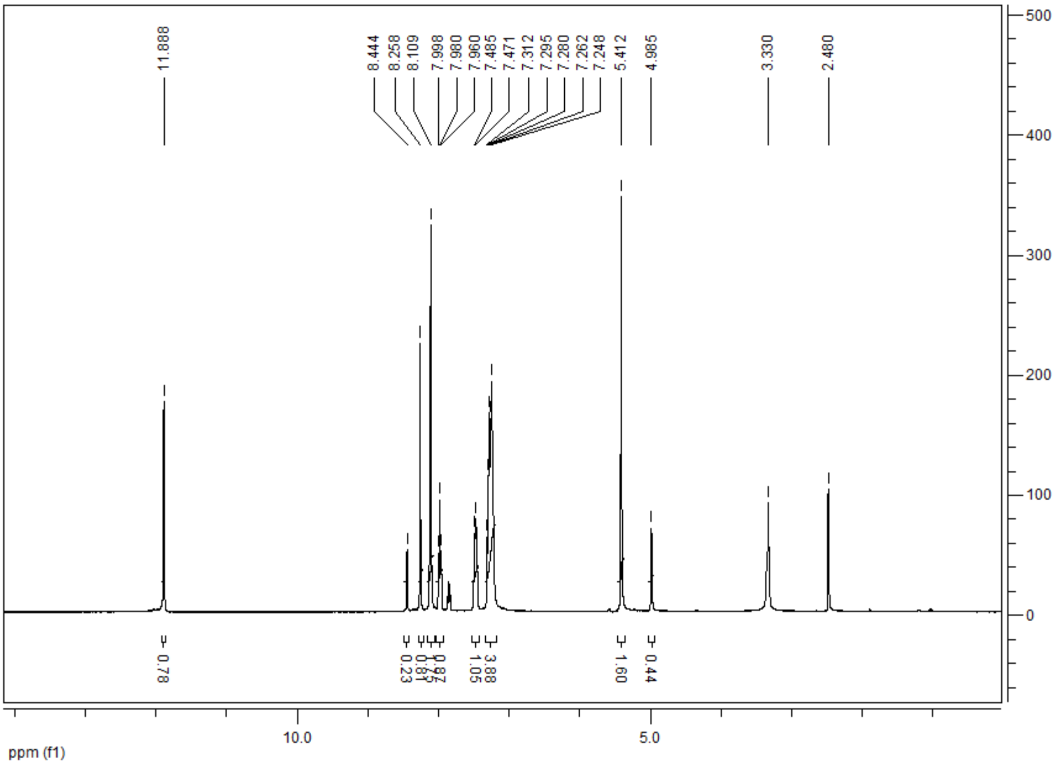


**Figure S3.** $^{1}$H-NMR spectra of compound **3a** (DMSO-$d_6$).

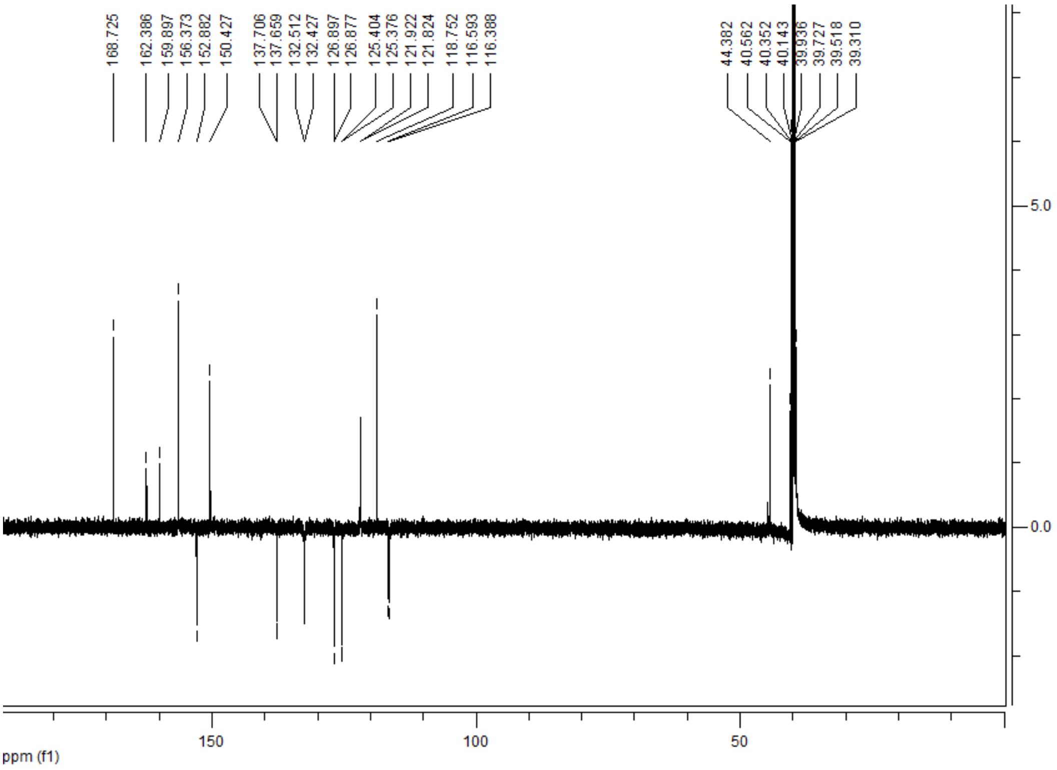


**Figure S4.** $^{13}$C-NMR spectra of compound **3a** (DMSO-$d_6$).

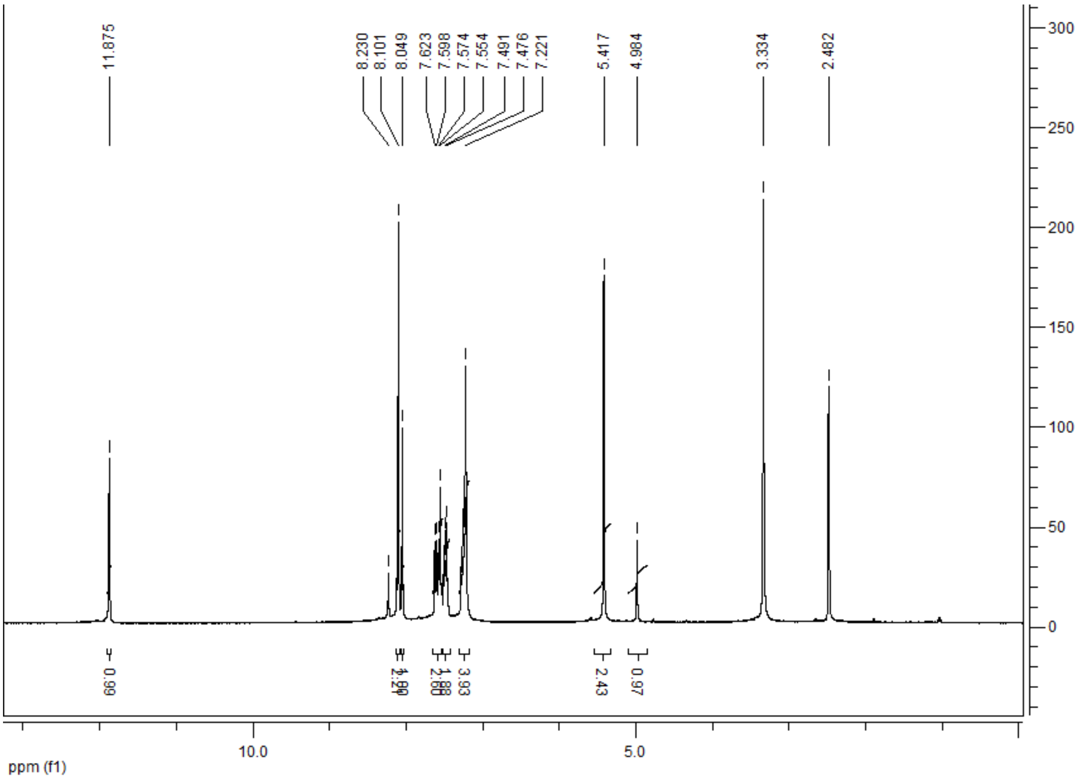


**Figure S5.** $^{1}$H-NMR spectra of compound **3b** (DMSO-$d_6$).

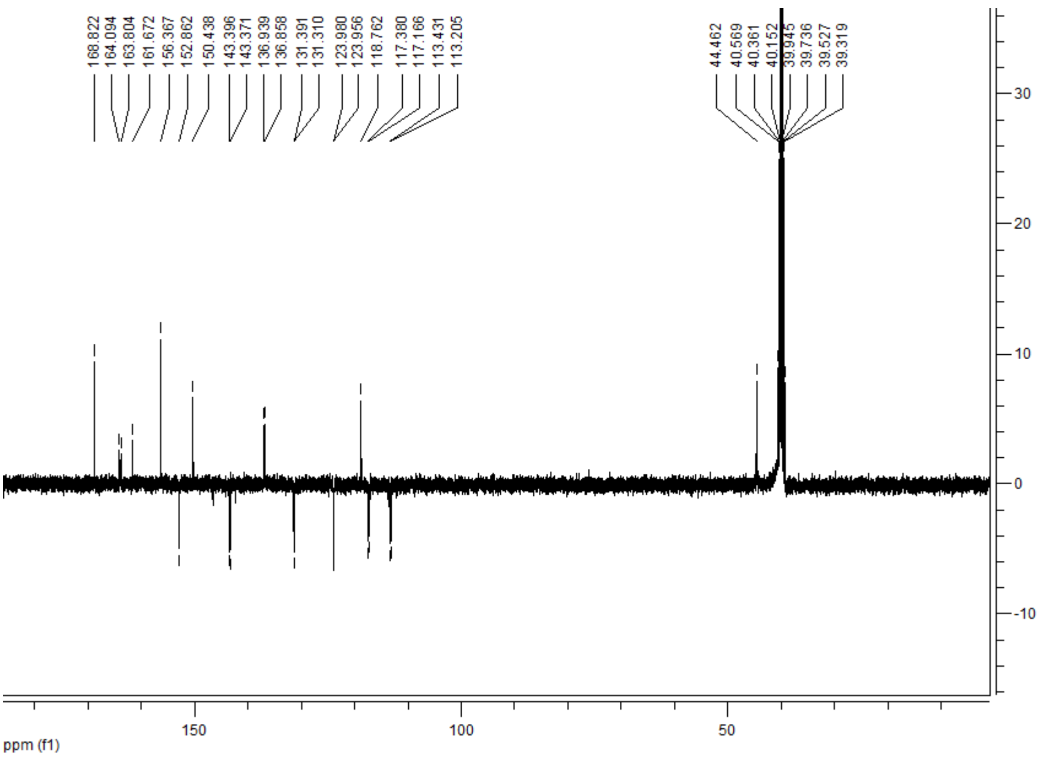

**Figure S6.** $^{13}$C-NMR spectra of compound **3b** (DMSO-$d_6$).

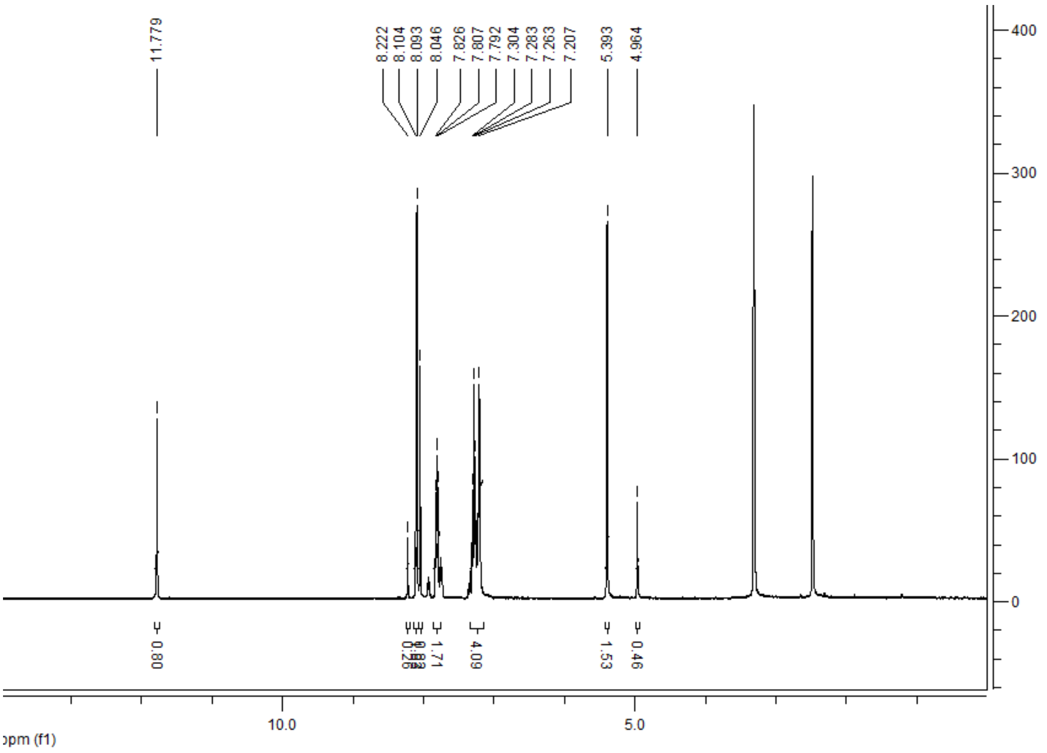

**Figure S7.** $^{1}$H-NMR spectra of compound **3c** (DMSO-$d_6$).

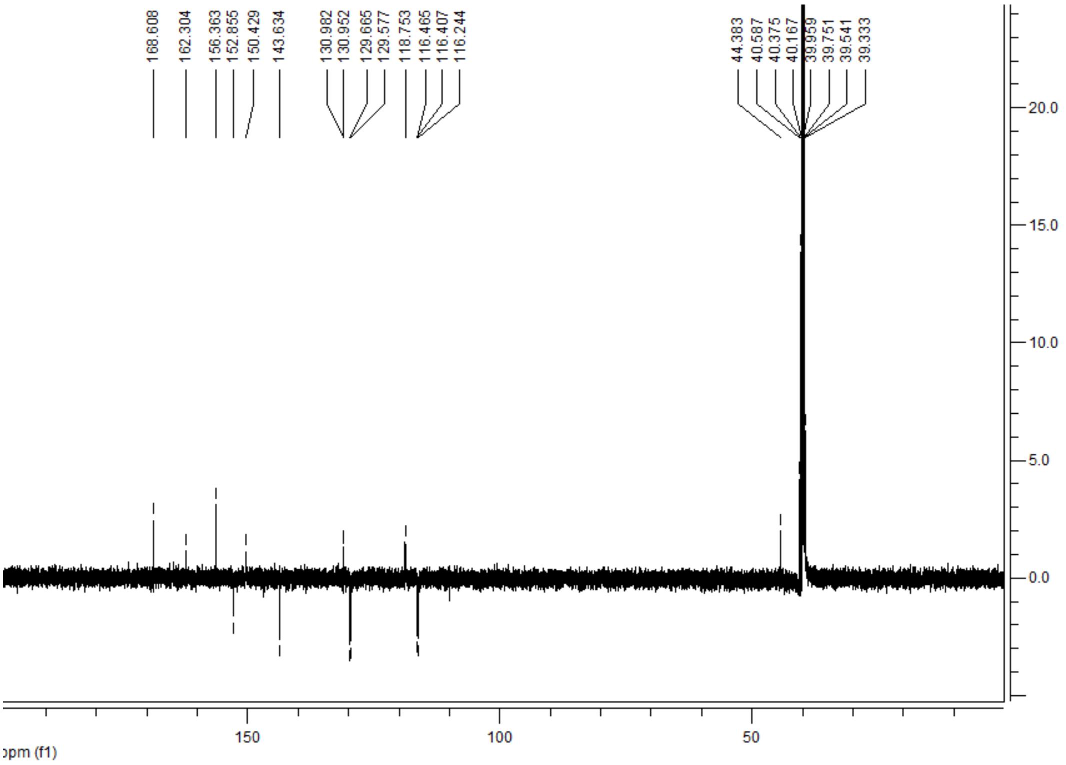


**Figure S8.** $^{13}$C-NMR spectra of compound **3c** (DMSO-$d_6$).

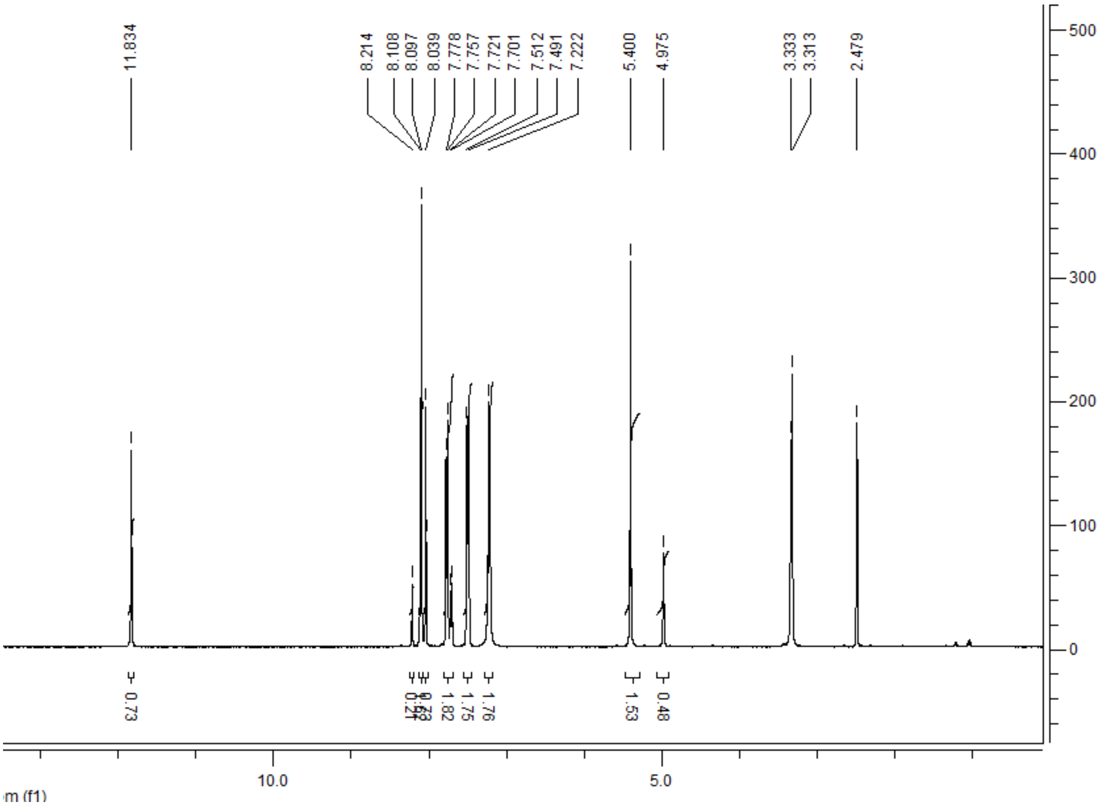


**Figure S9.** $^{1}$H-NMR spectra of compound **3d** (DMSO-$d_6$).

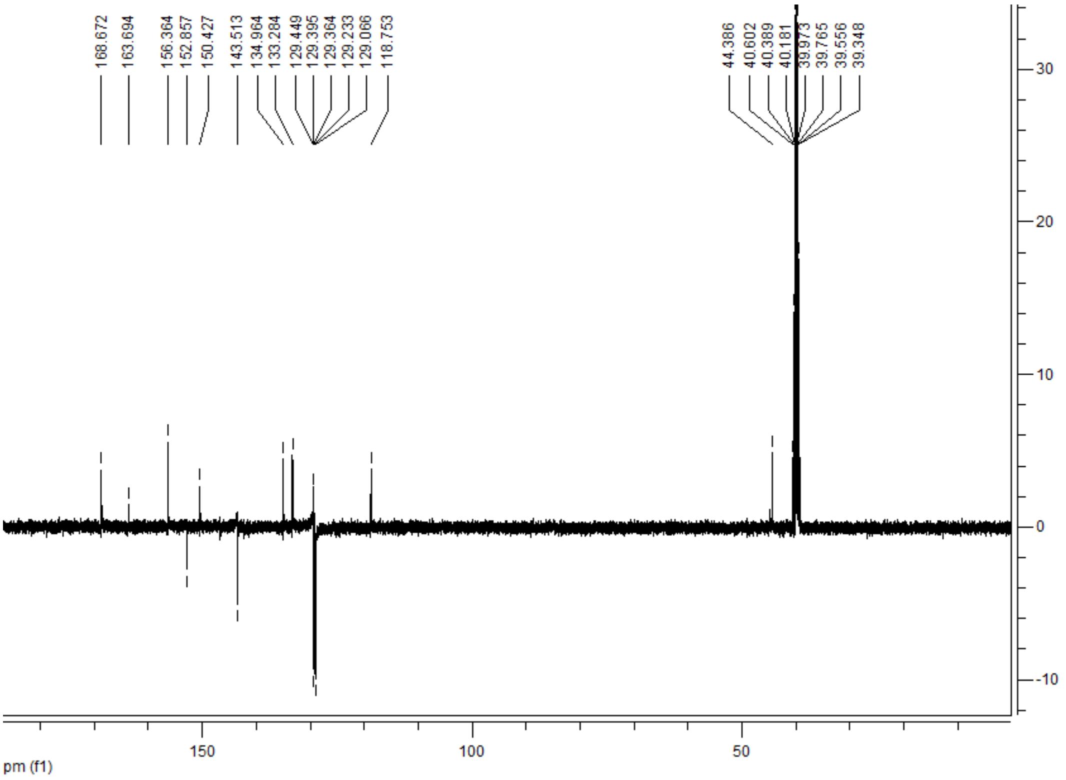


**Figure S10.** $^{13}$C-NMR spectra of compound **3d** (DMSO-$d_6$).

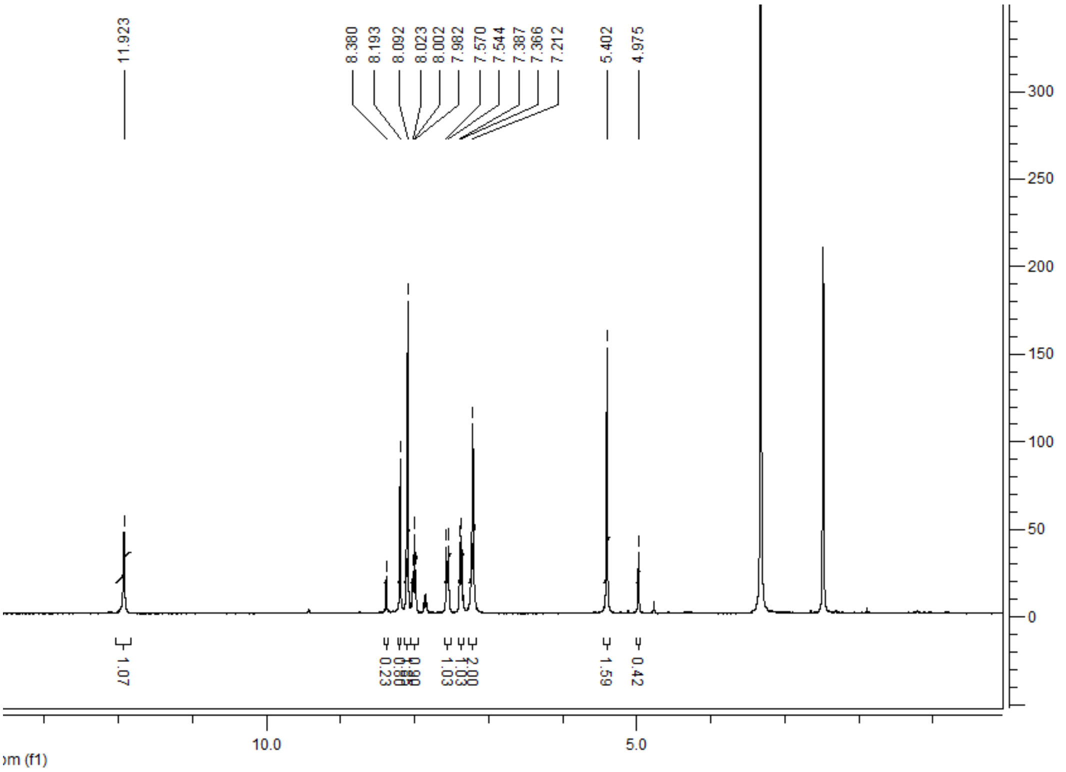


**Figure S11.** $^{1}$H-NMR spectra of compound **3e** (DMSO-$d_6$).

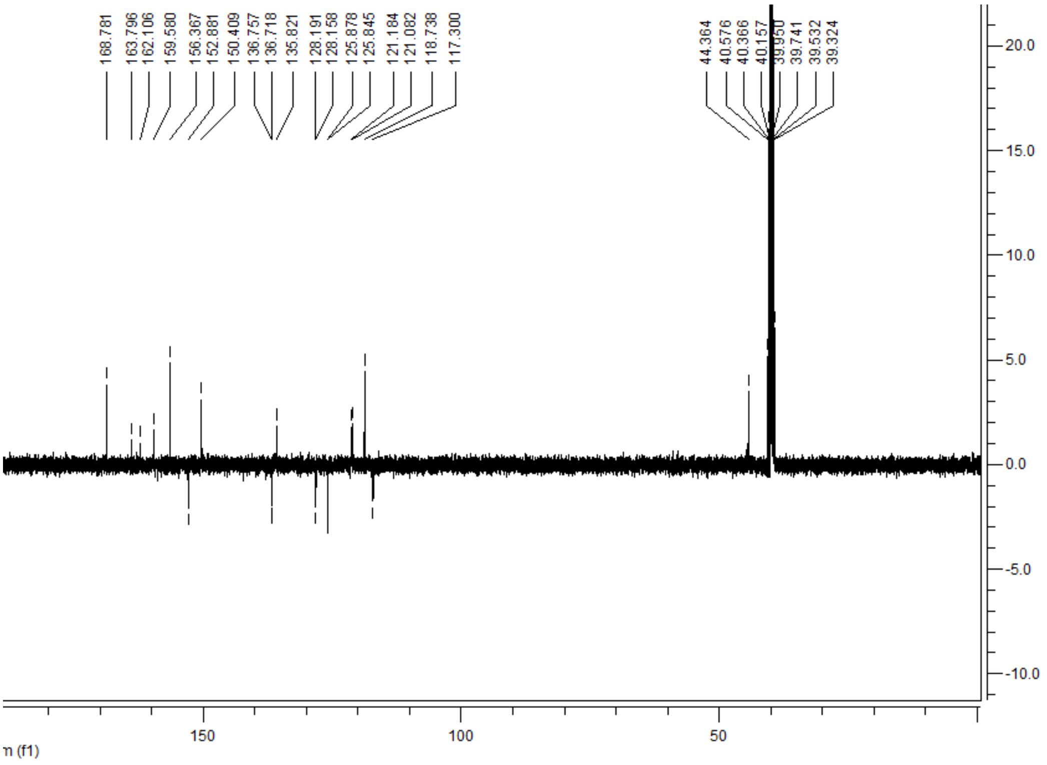


**Figure S12.** $^{13}$C-NMR spectra of compound **3e** (DMSO-$d_6$).

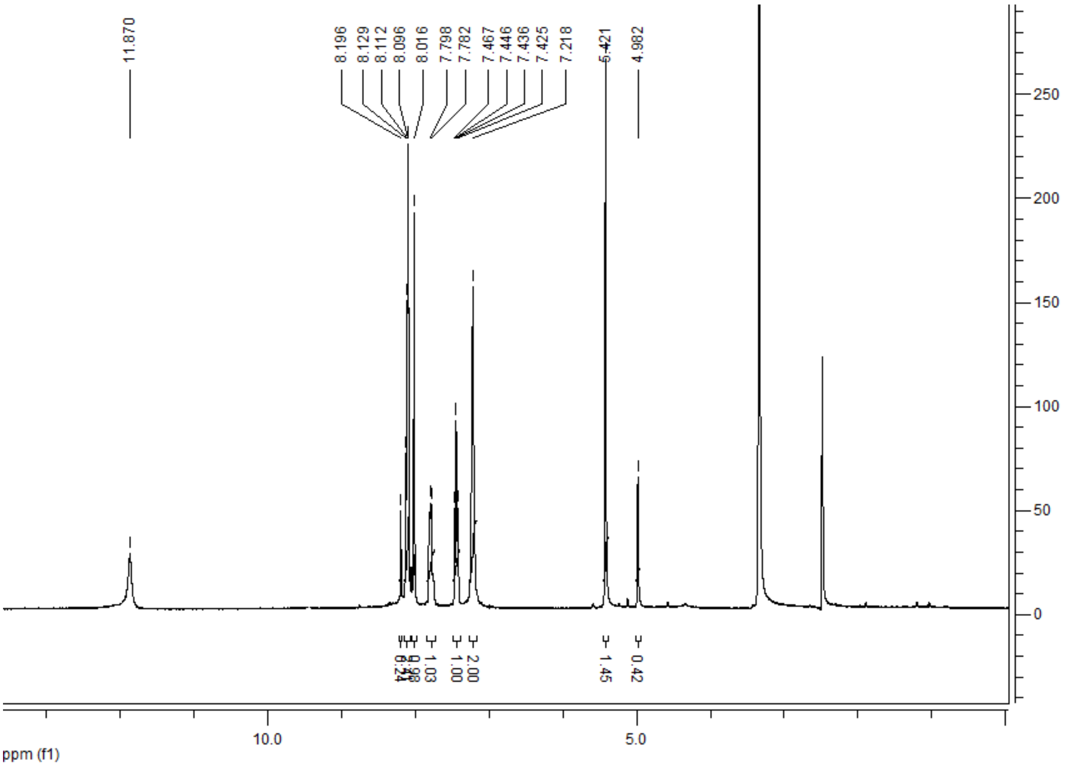


**Figure S13.** $^{1}$H-NMR spectra of compound **3f** (DMSO-$d_6$).

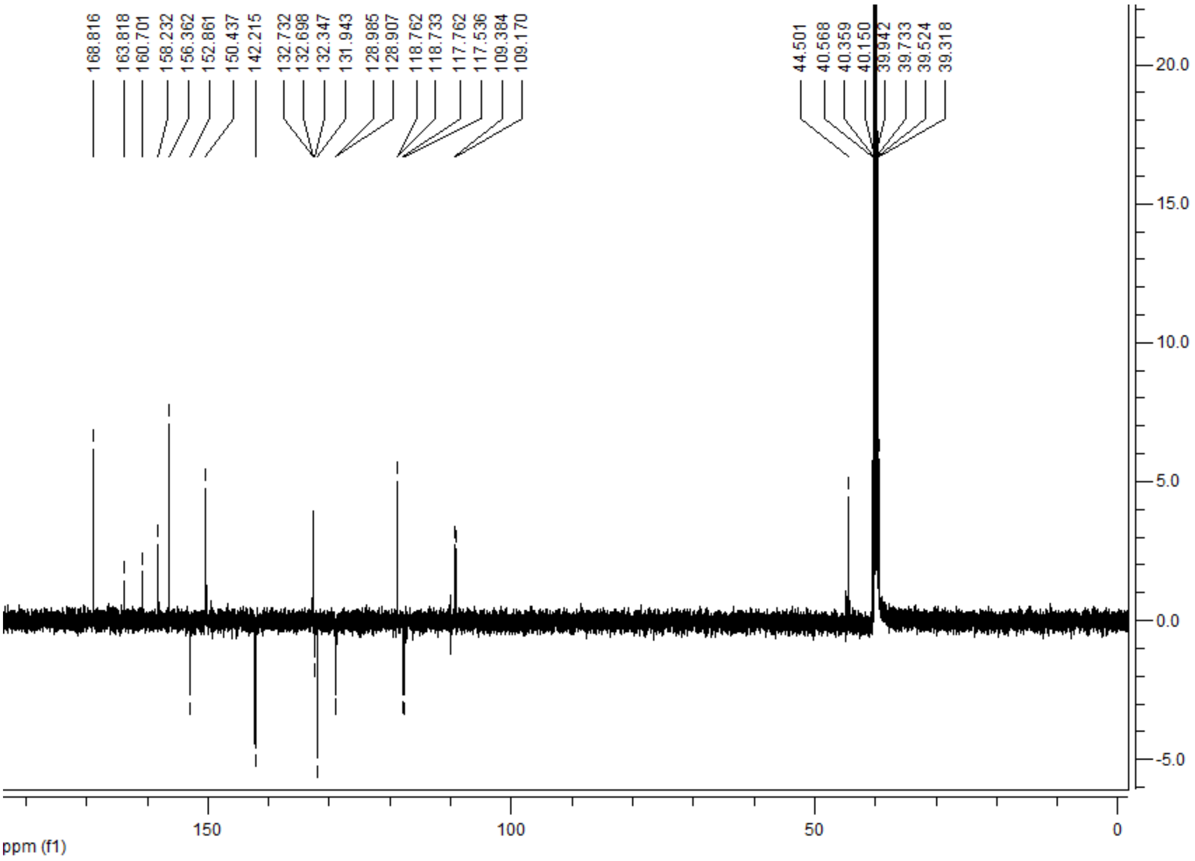


**Figure S14.** $^{13}$C-NMR spectra of compound **3f** (DMSO-$d_6$).

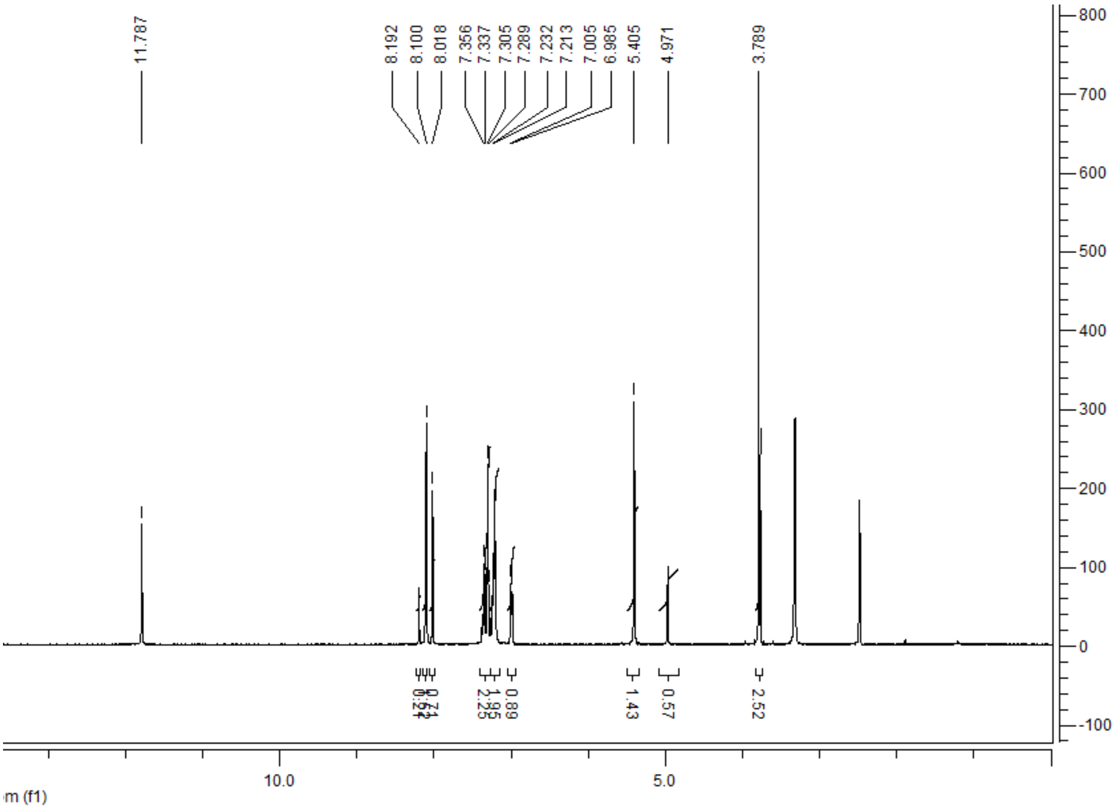


**Figure S15.** $^{1}$H-NMR spectra of compound **3g** (DMSO-$d_6$).

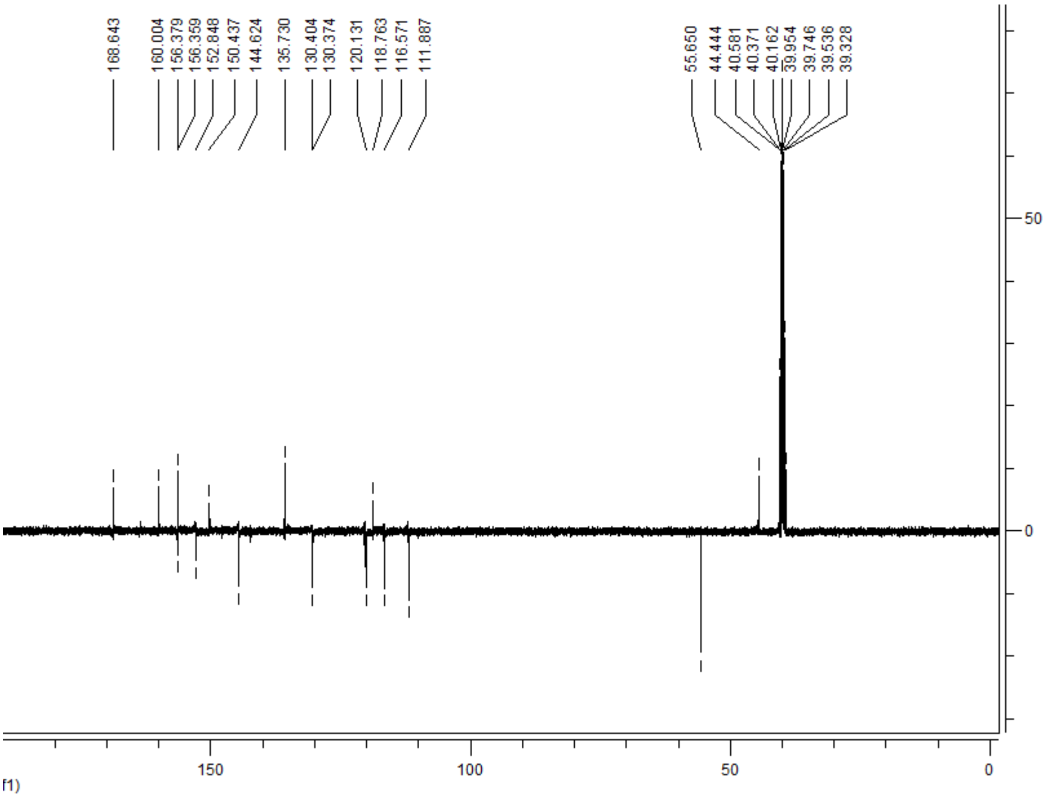


**Figure S16.** $^{13}$C-NMR spectra of compound **3g** (DMSO-$d_6$).

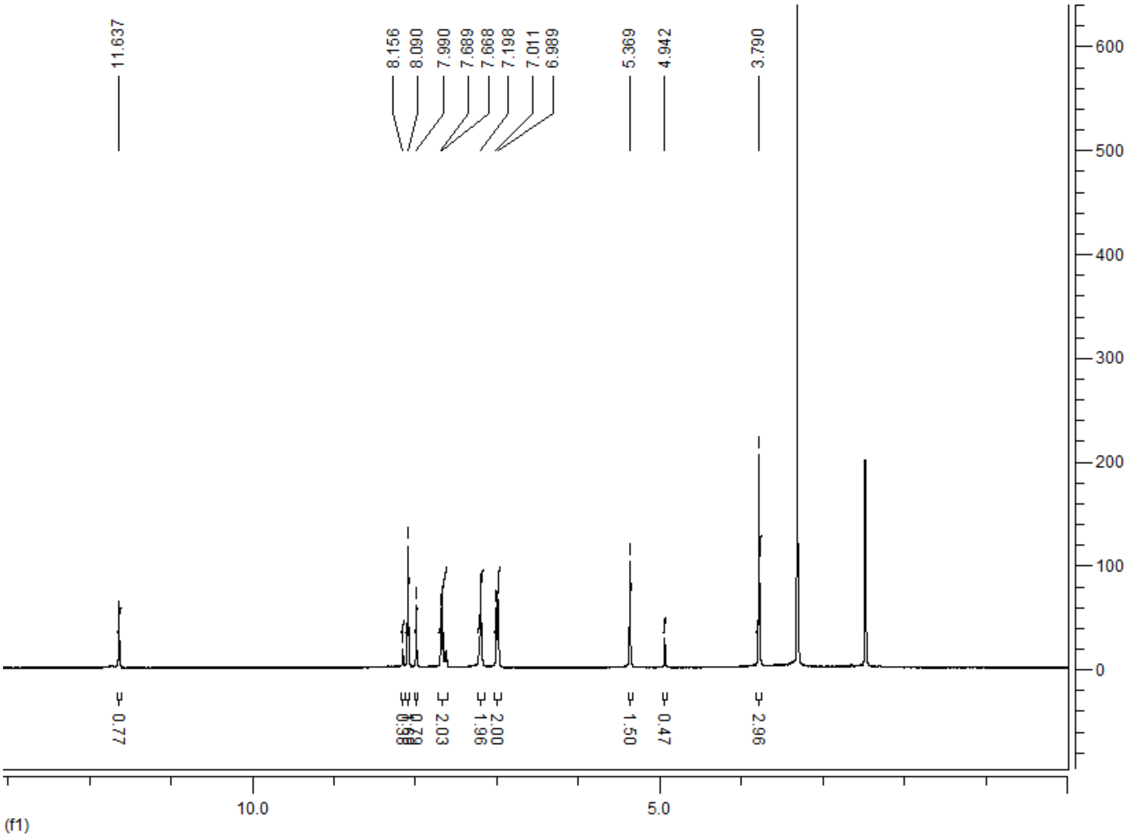


**Figure S17.** $^{1}$H-NMR spectra of compound **3h** (DMSO-$d_6$).

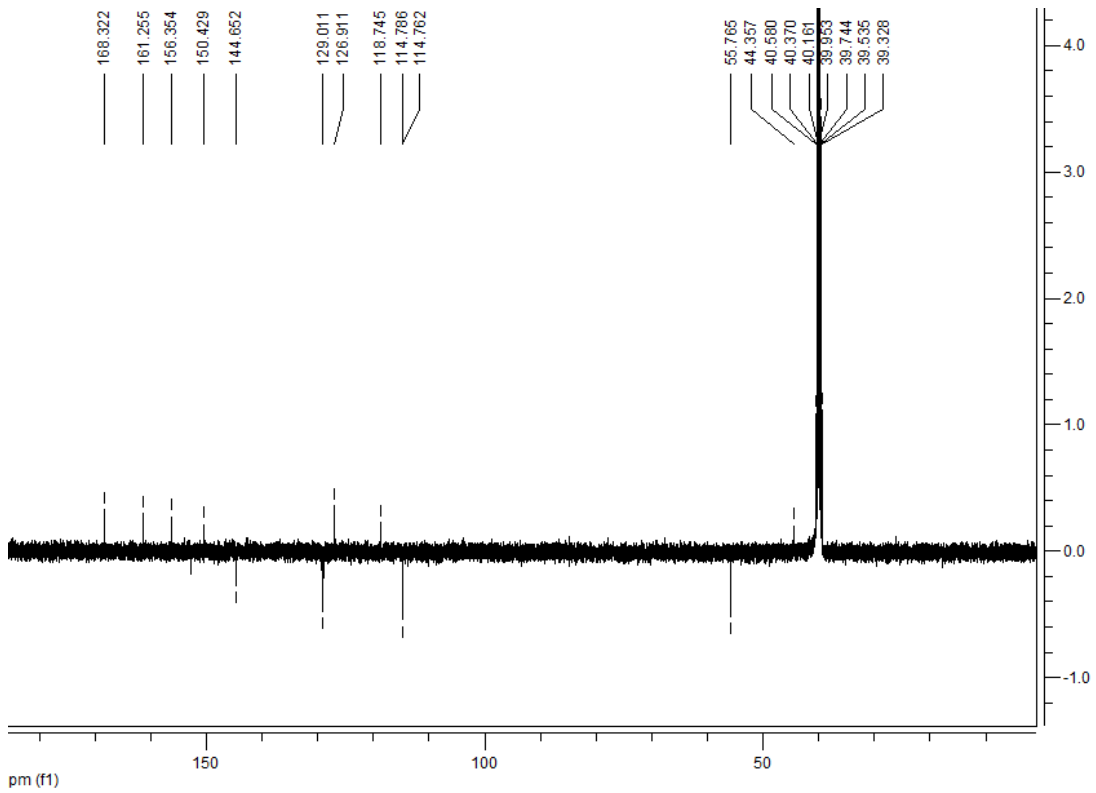


**Figure S18.** $^{13}$C-NMR spectra of compound **3h** (DMSO-$d_6$).

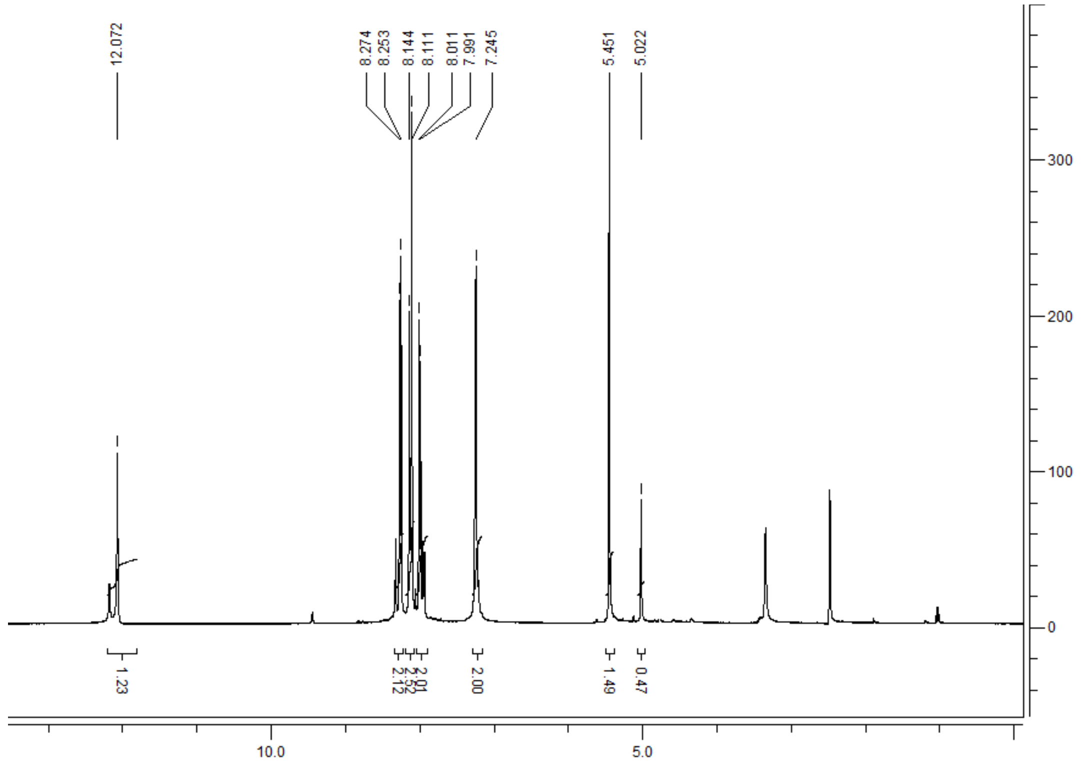


**Figure S19.** $^{1}$H-NMR spectra of compound **3i** (DMSO-$d_6$).

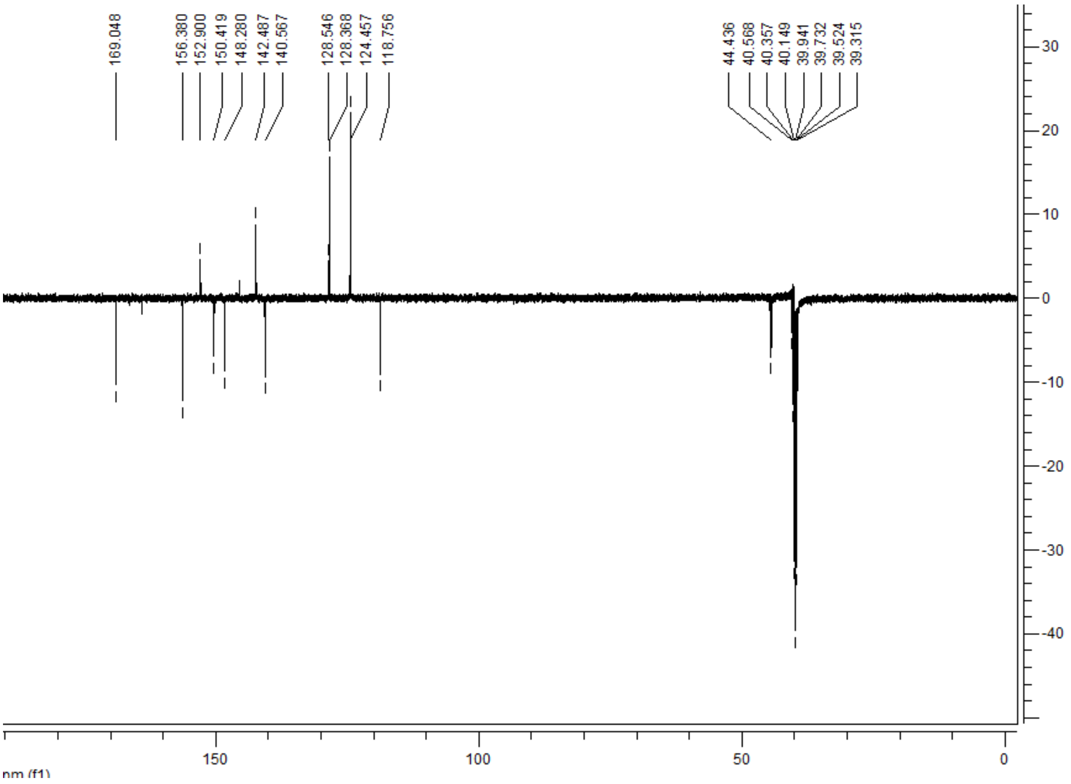


**Figure S20.** $^{13}$C-NMR spectra of compound **3i** (DMSO-$d_6$).

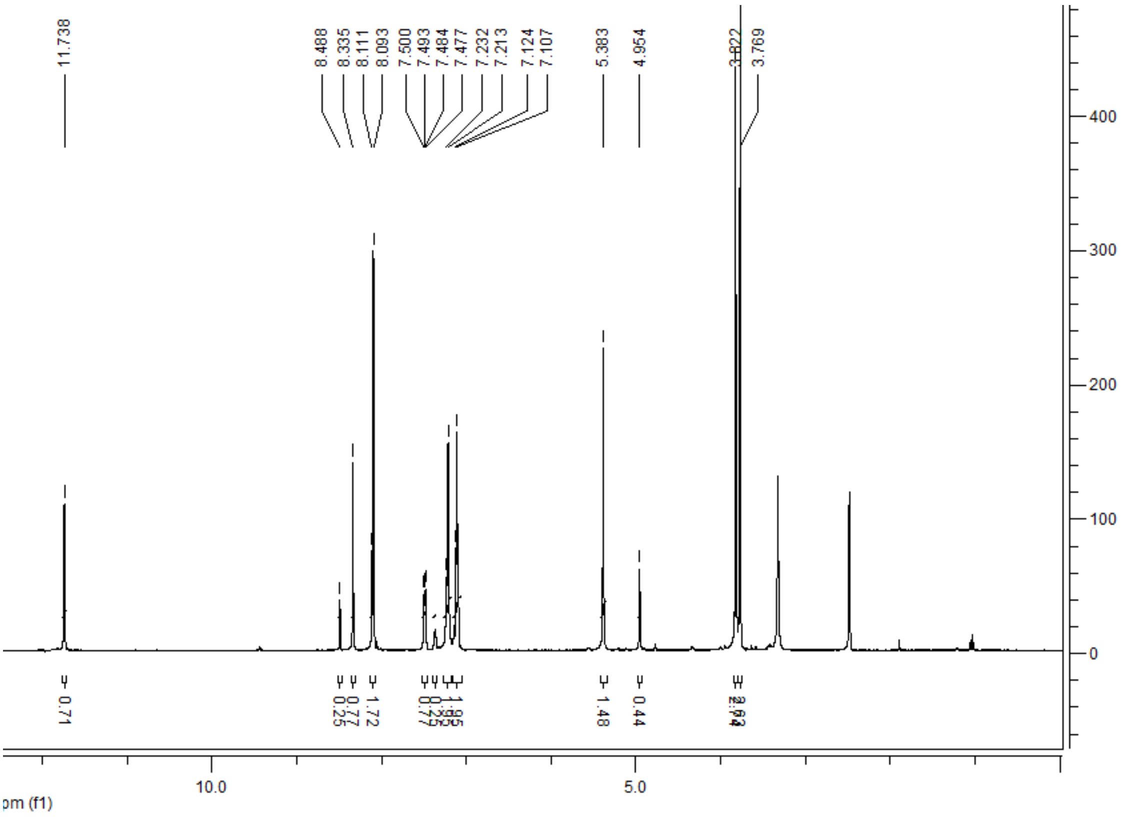


**Figure S21.** $^{1}$H-NMR spectra of compound **3j** (DMSO-$d_6$).

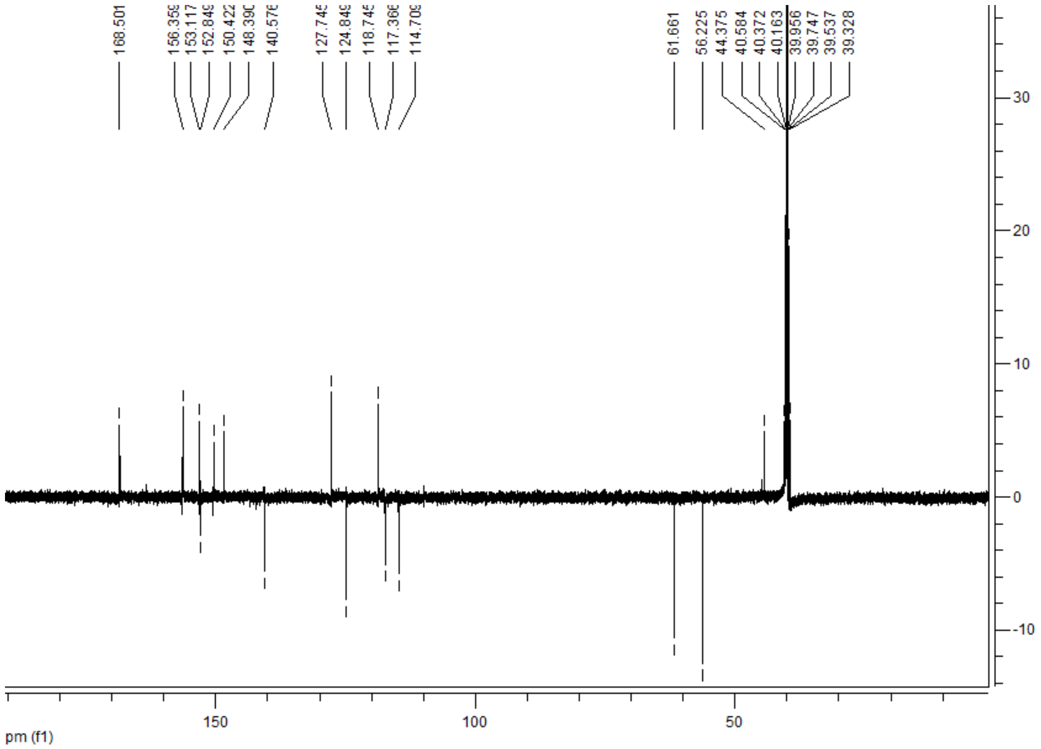


**Figure S22.** $^{13}$C-NMR spectra of compound **3j** (DMSO-$d_6$).

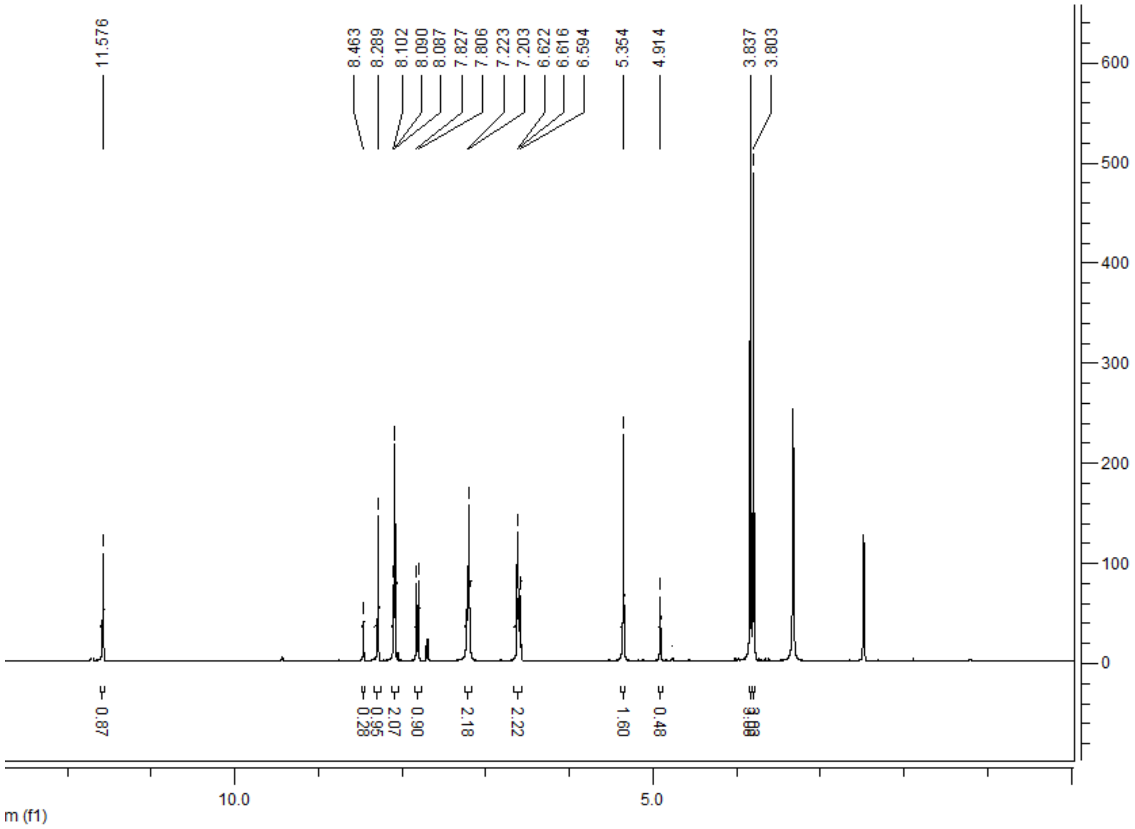


**Figure S23.** $^{1}$H-NMR spectra of compound **3k** (DMSO-$d_6$).

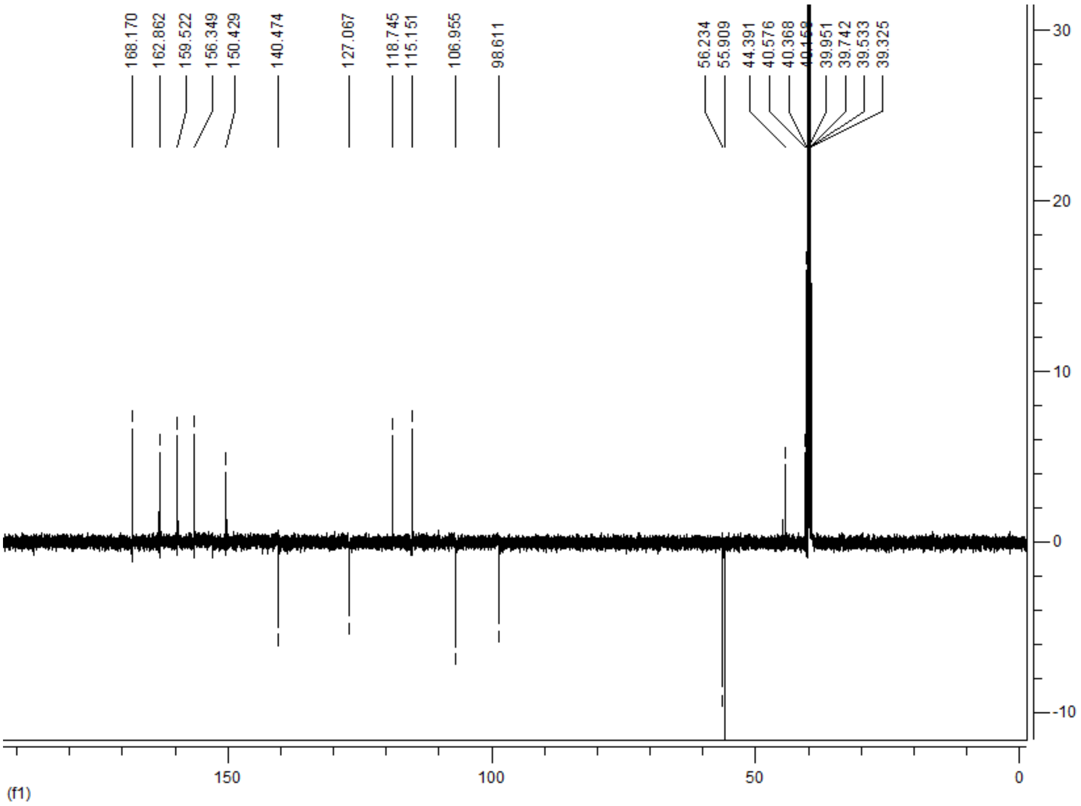


**Figure S24.** $^{13}$C-NMR spectra of compound **3k** (DMSO-*d*$_6$).

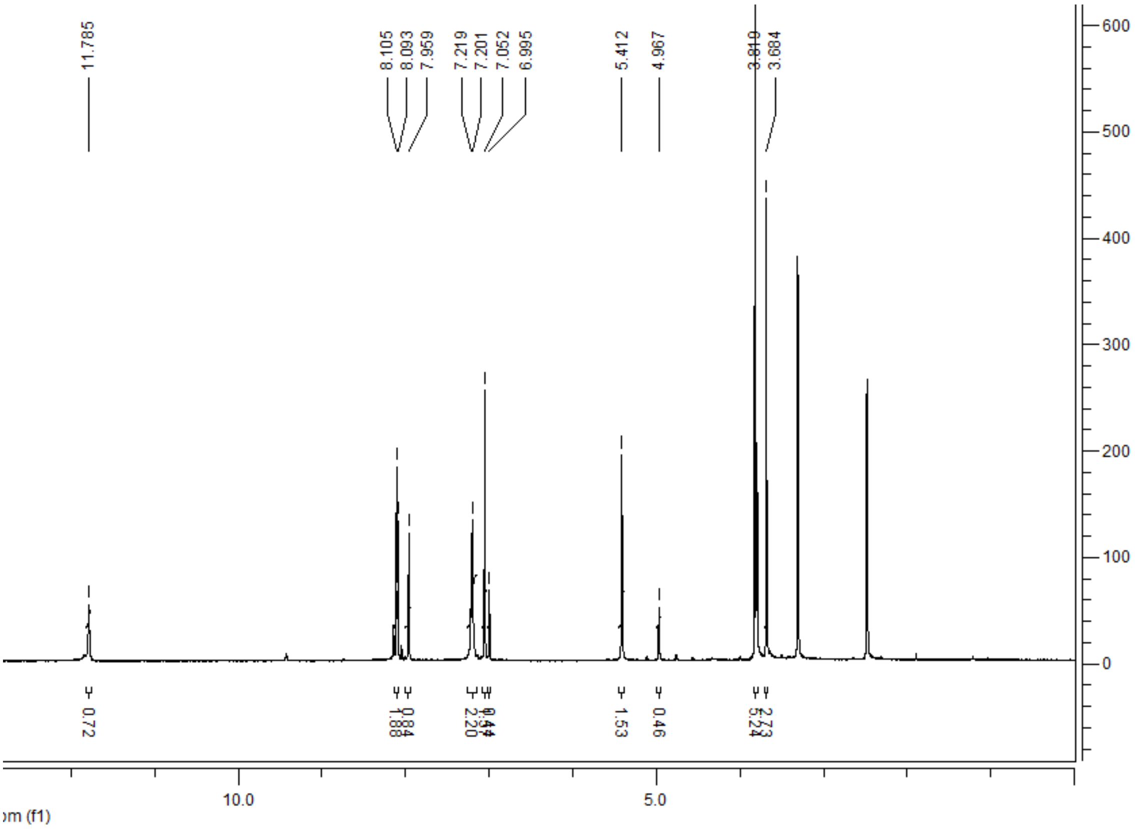


**Figure S25.** $^{1}$H-NMR spectra of compound **3l** (DMSO-*d*$_6$).

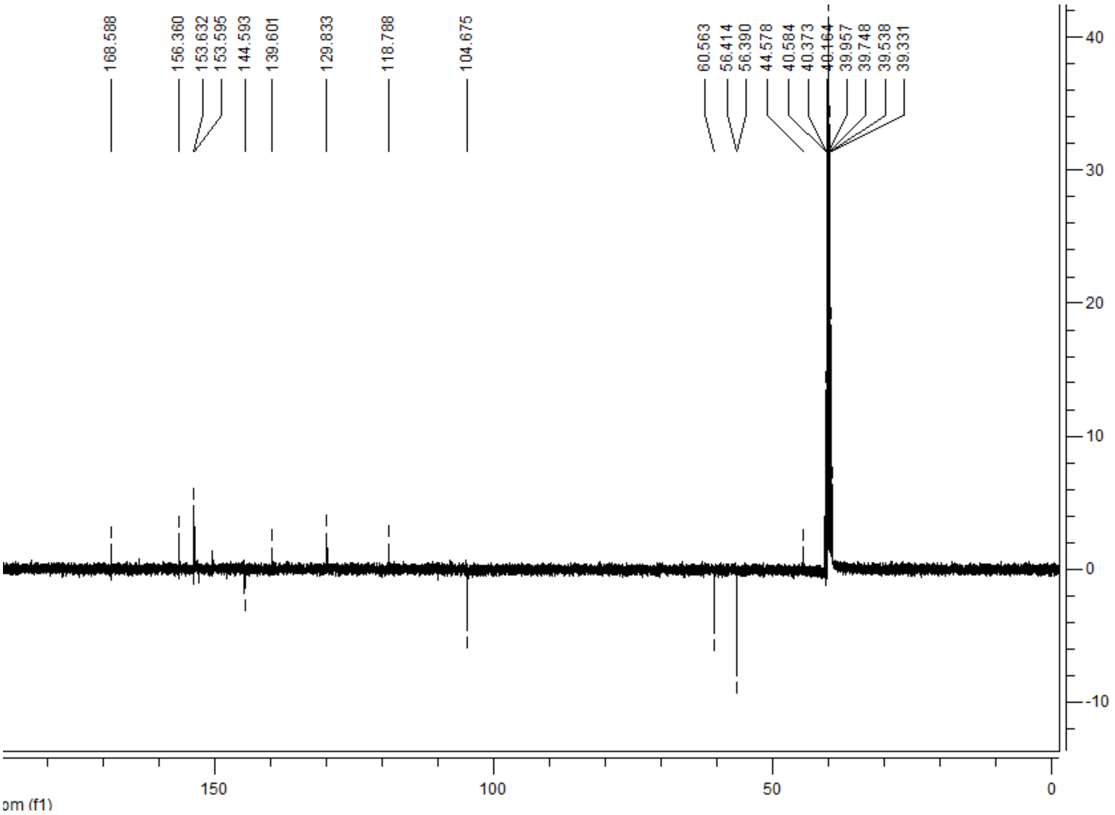


**Figure S26.** $^{13}$C-NMR spectra of compound **3l** (DMSO-$d_6$).

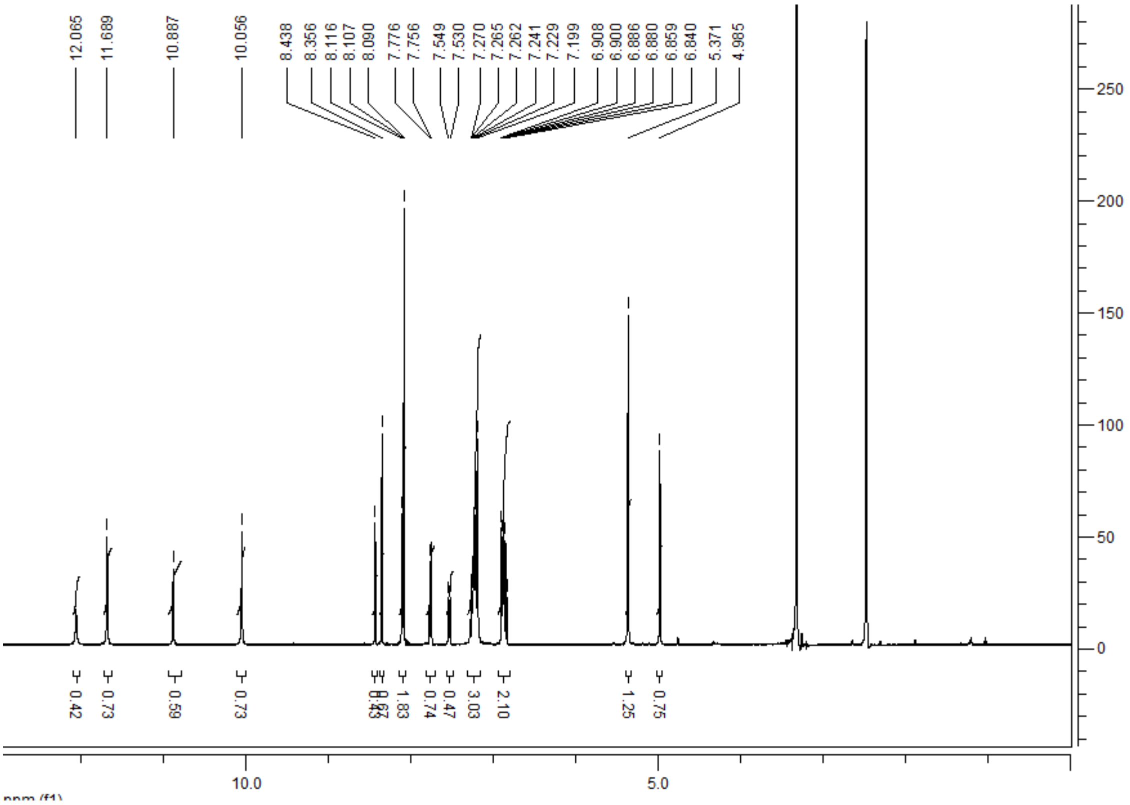


**Figure S27.** $^{1}$H-NMR spectra of compound **3m** (DMSO-$d_6$).

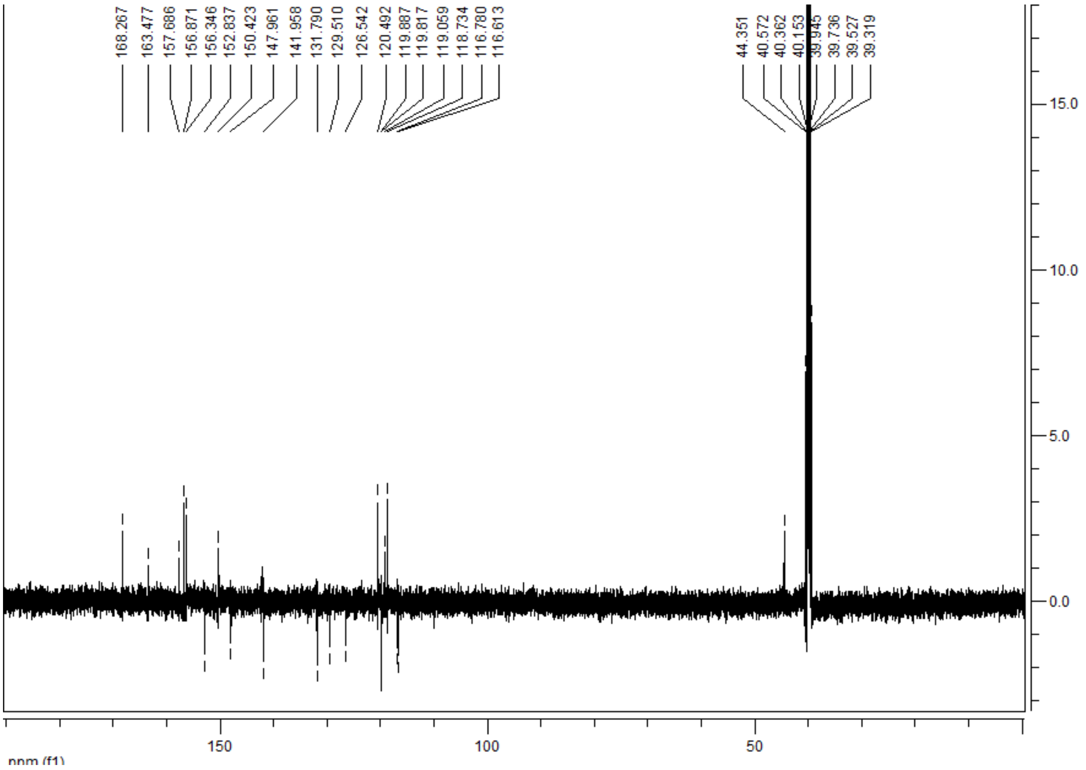


**Figure S28.** $^{13}$C-NMR spectra of compound **3m** (DMSO-*d*$_6$).

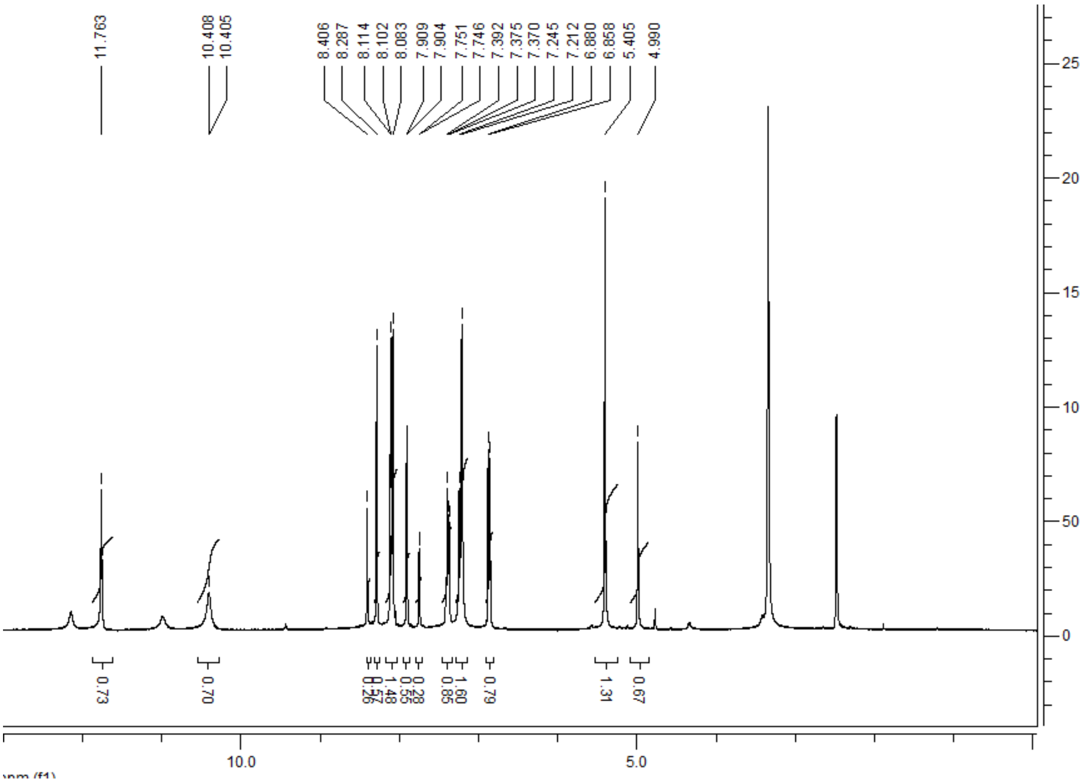


**Figure S29.** $^{1}$H-NMR spectra of compound **3n** (DMSO-*d*$_6$).

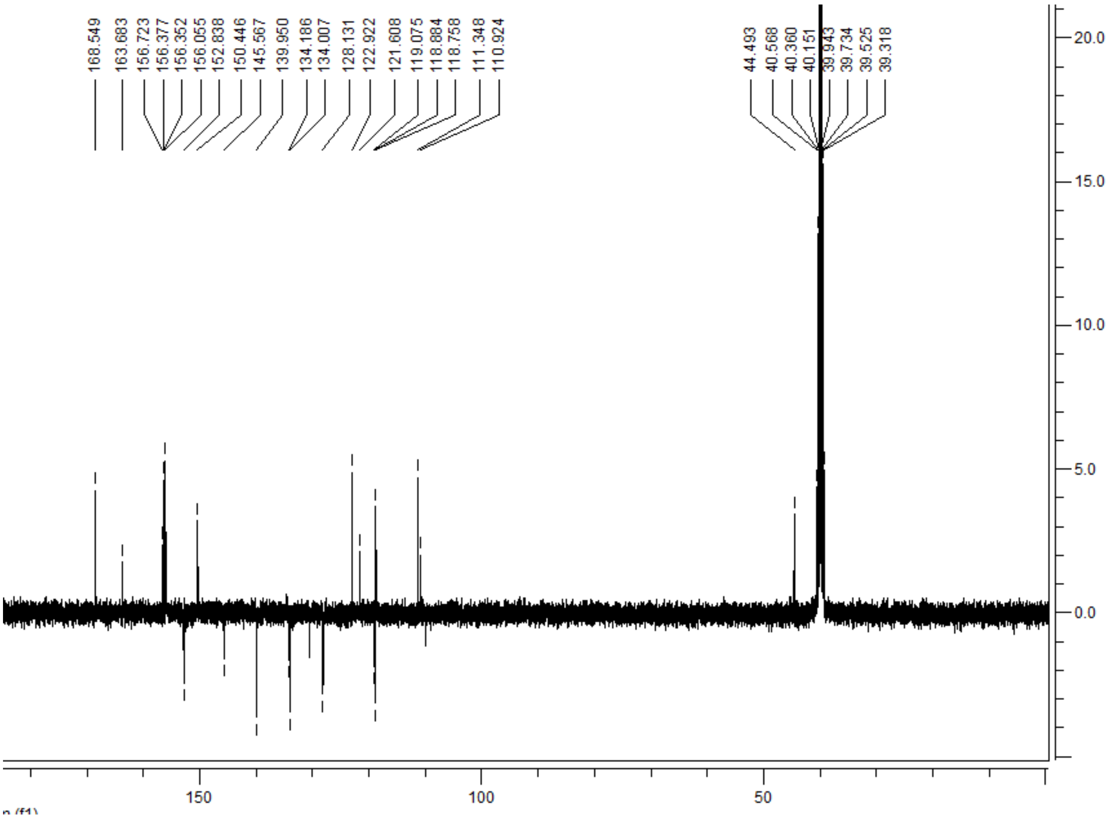


**Figure S30.** $^{13}C$-NMR spectra of compound **3n** (DMSO-$d_6$).

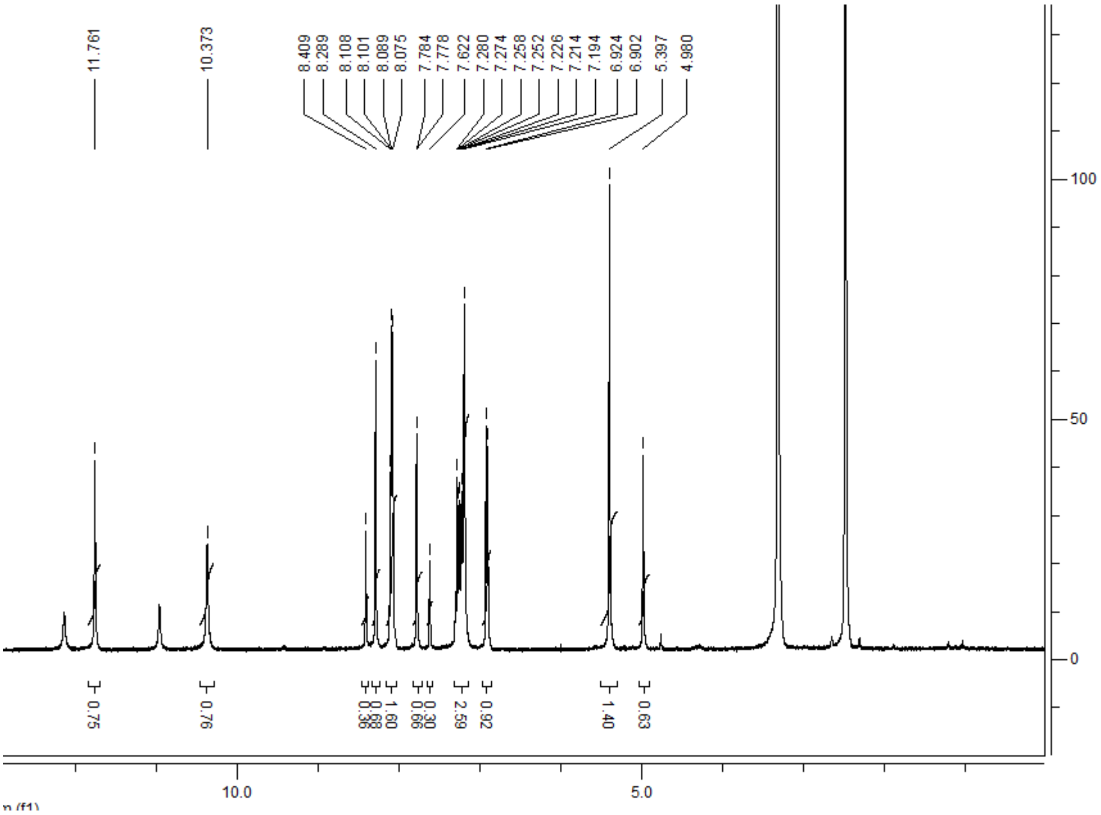


**Figure S31.** $^{1}H$-NMR spectra of compound **3o** (DMSO-$d_6$).

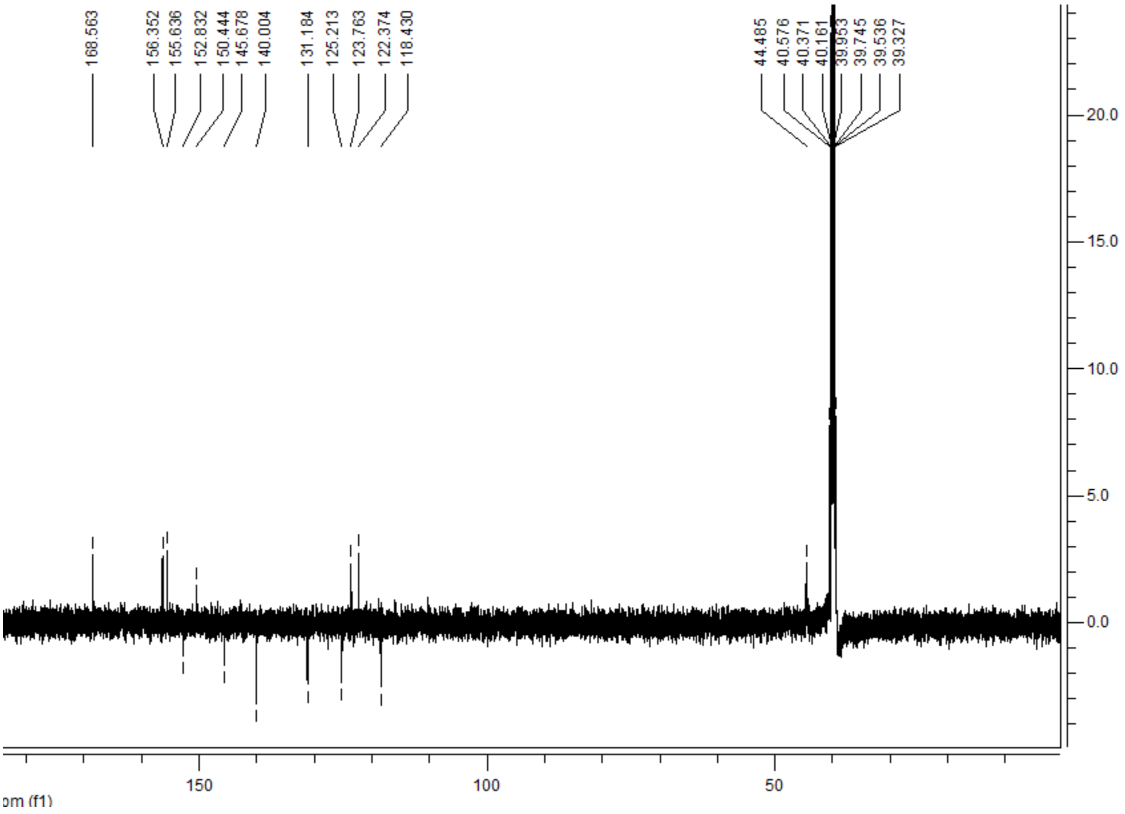


**Figure S32.** $^{13}$C-NMR spectra of compound **3o** (DMSO-$d_6$).

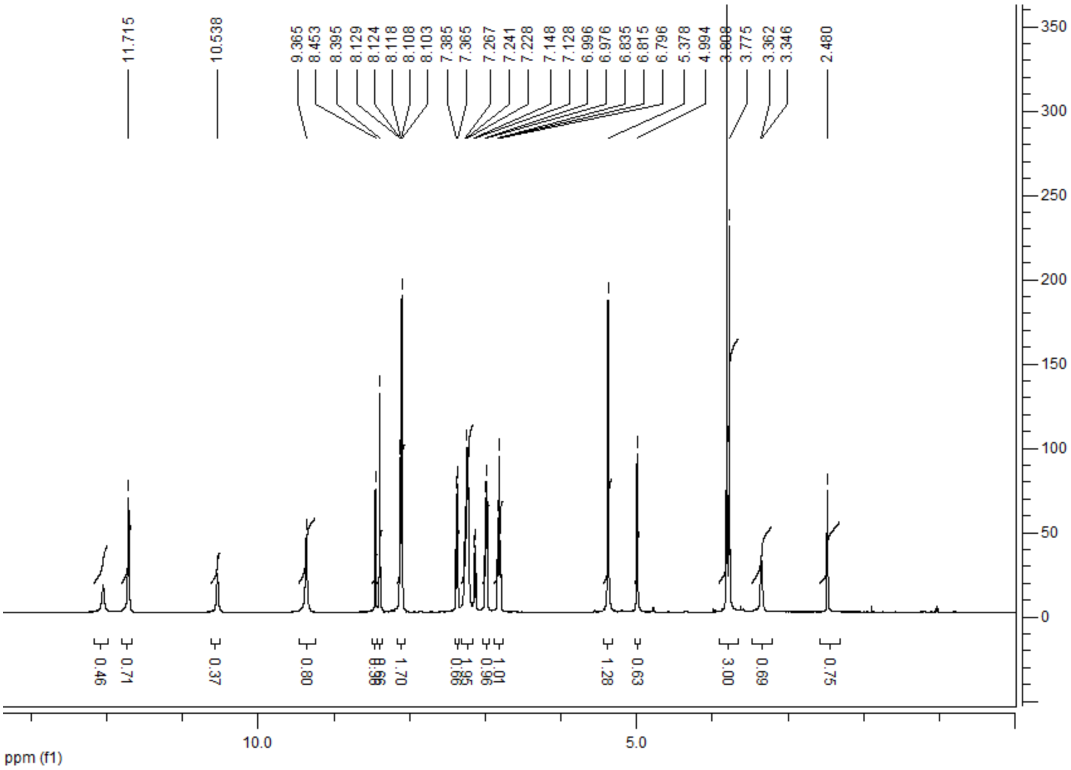


**Figure S33.** $^{1}$H-NMR spectra of compound **3p** (DMSO-$d_6$).

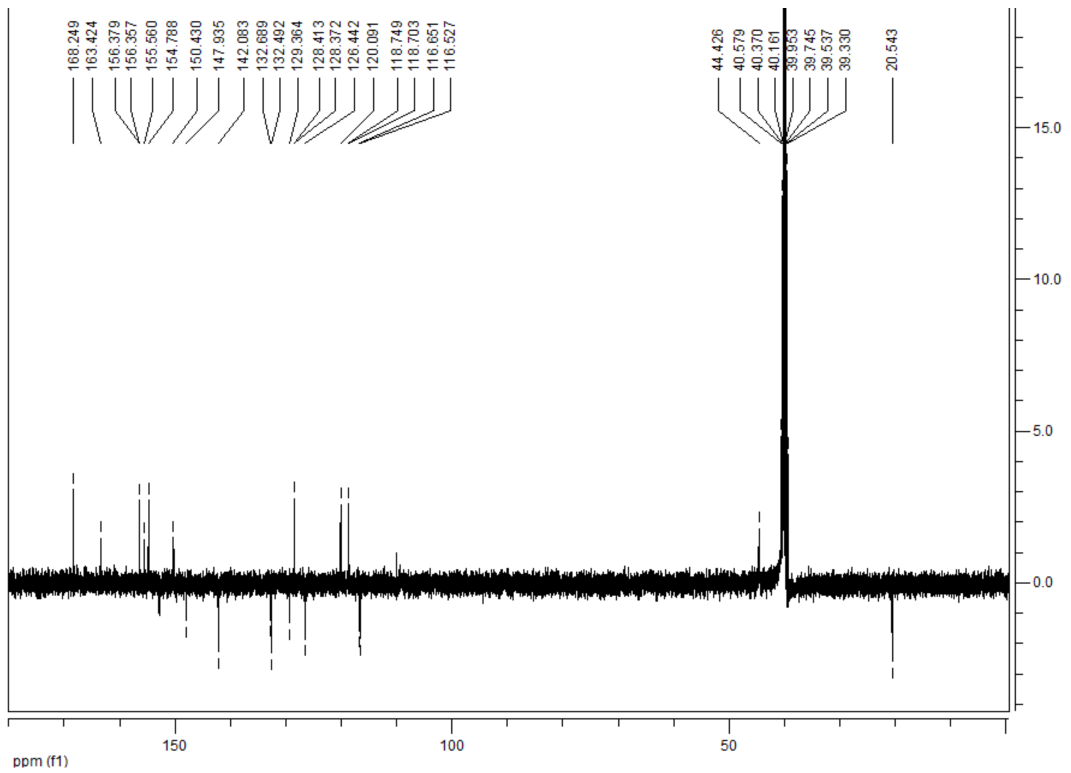

**Figure S34.** $^{13}$C-NMR spectra of compound **3p** (DMSO-*d*$_6$).

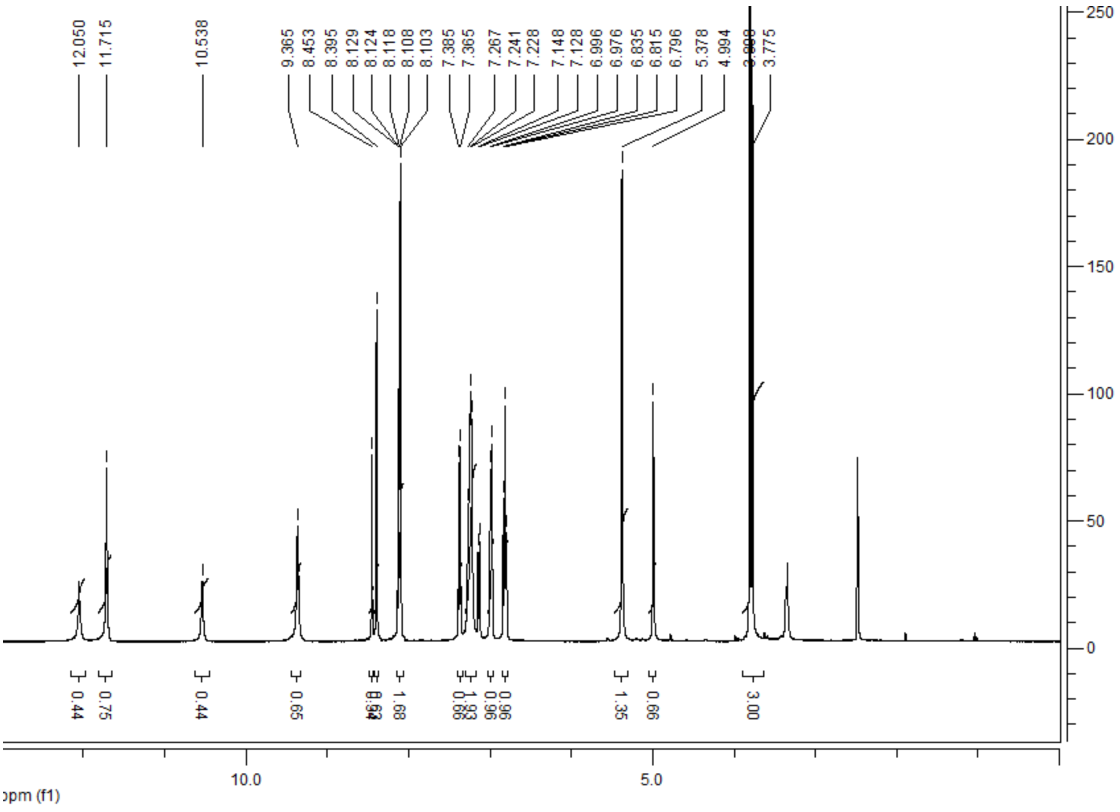

**Figure S35.** $^{1}$H-NMR spectra of compound **3q** (DMSO-*d*$_6$).

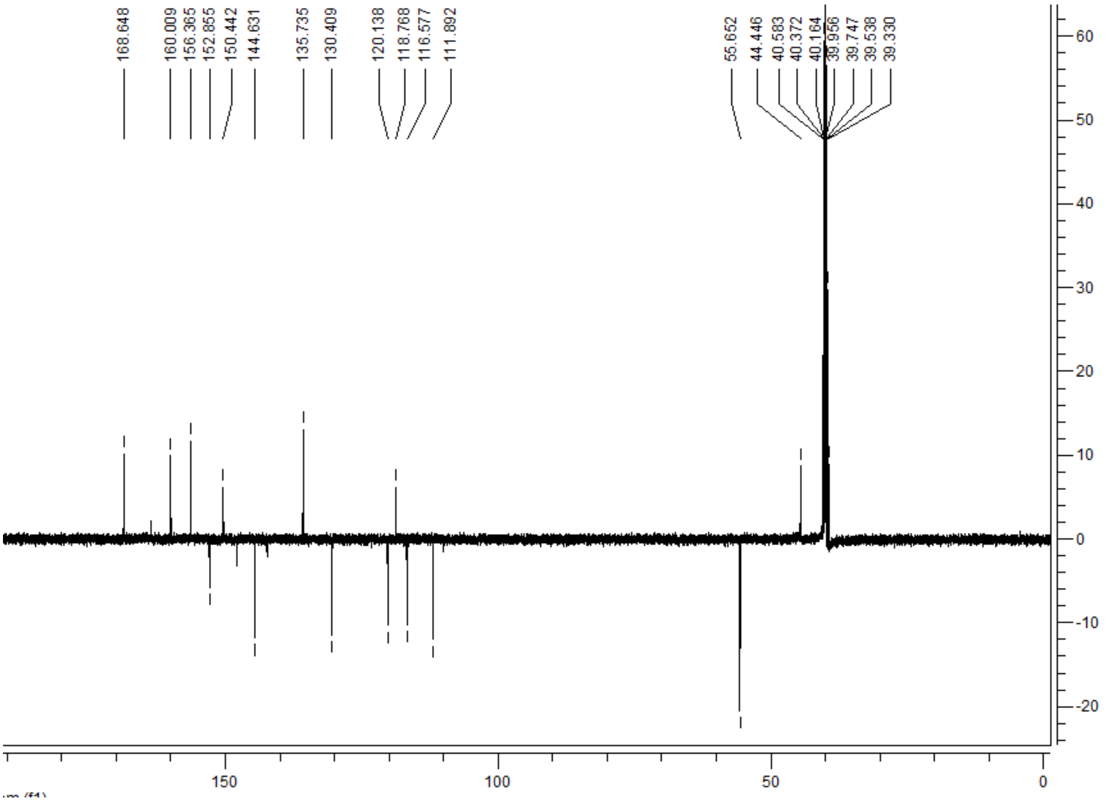


**Figure S36.** $^{13}$C-NMR spectra of compound **3q** (DMSO-*d*$_6$).

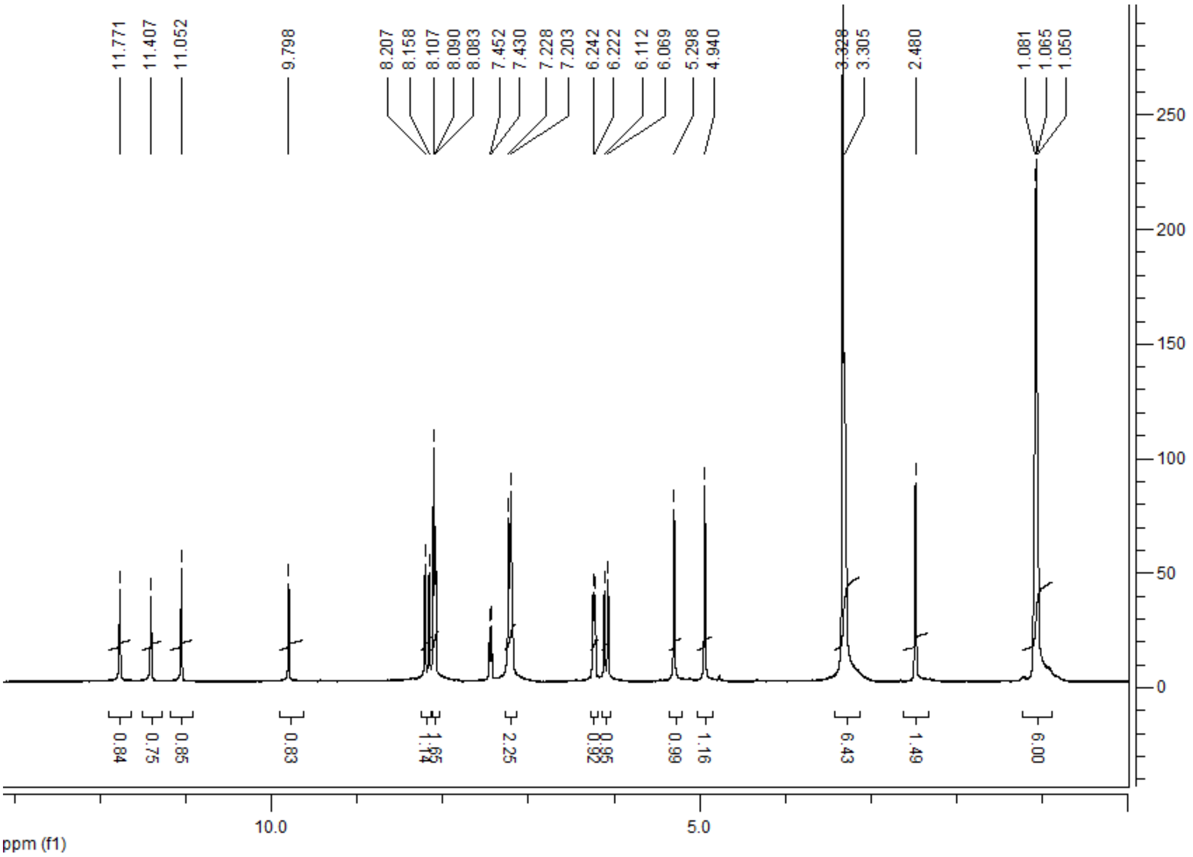


**Figure S37.** $^{1}$H-NMR spectra of compound **3r** (DMSO-*d*$_6$).

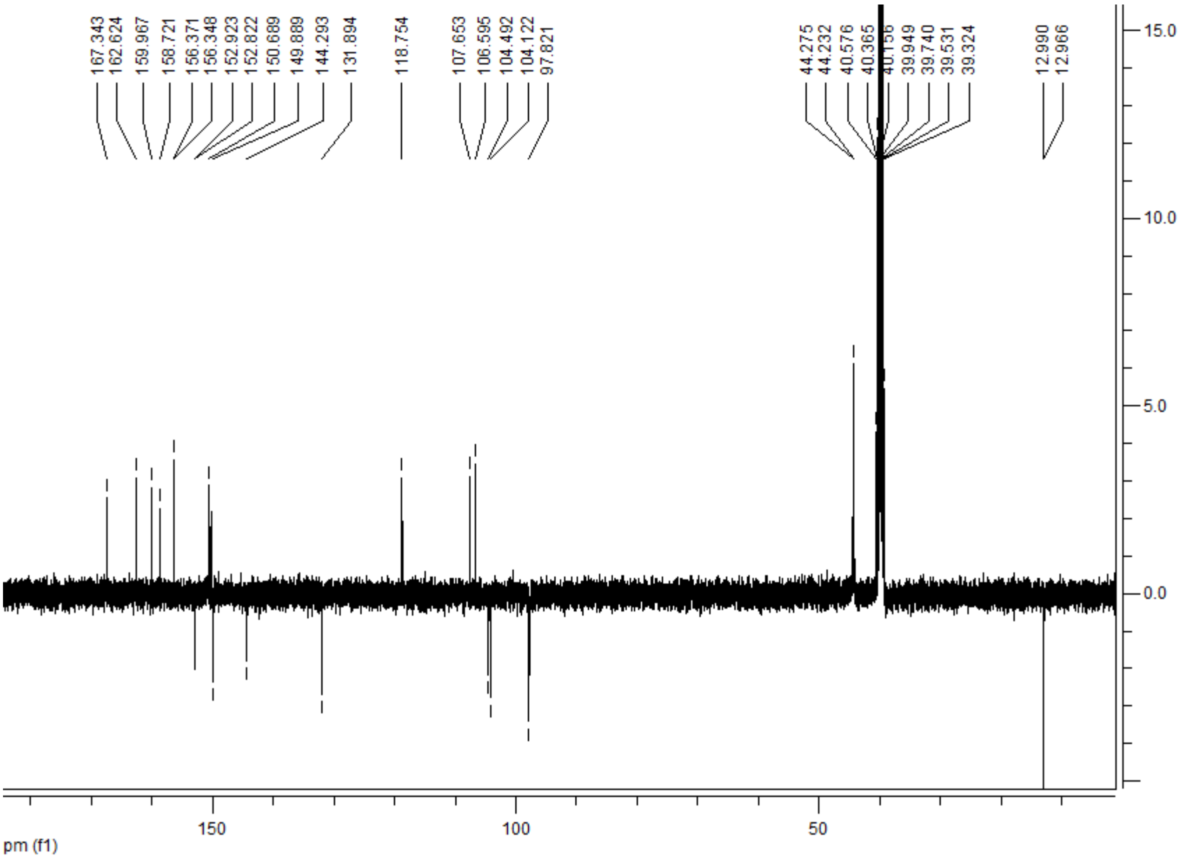


**Figure S38.** $^{13}$C-NMR spectra of compound **3r** (DMSO-$d_6$).

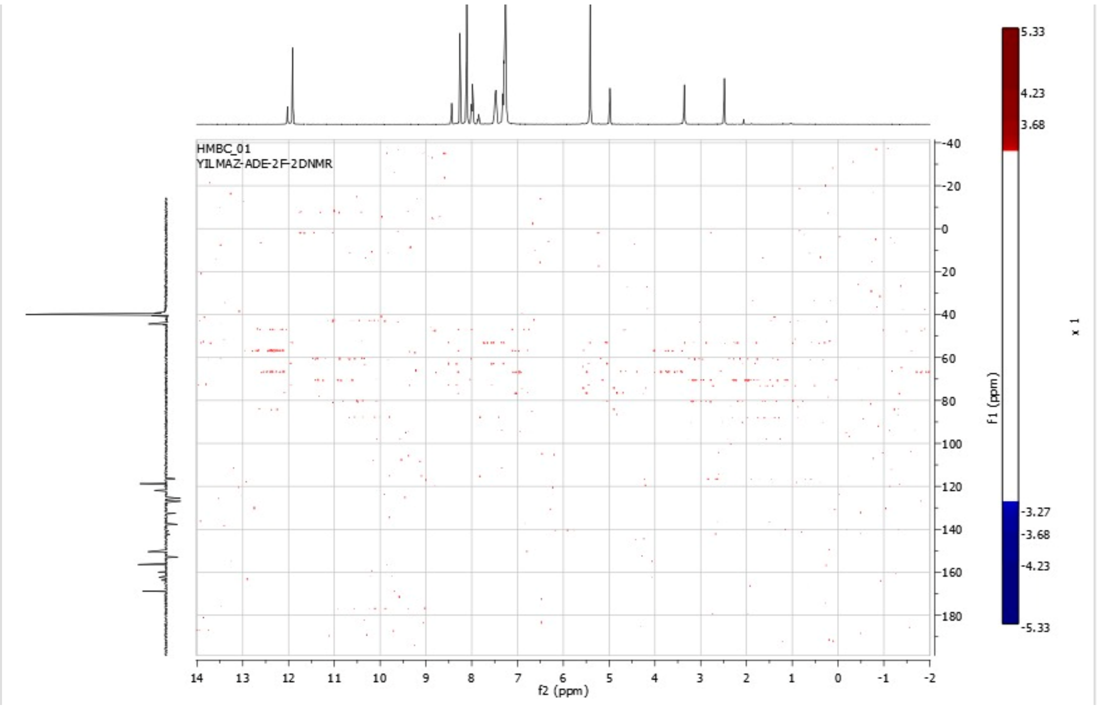


**Figure S39.** 2D-NMR (HMBC) spectra of compound **3a** (DMSO-$d_6$).
